# Deprescribing: What is the gold standard? Themes that characterized the discussions at the first Danish symposium on evidence-based deprescribing

**DOI:** 10.1016/j.rcsop.2022.100102

**Published:** 2022-01-07

**Authors:** Lykke I. Kaas Oldenburg, Dagmar Dalin, Anne Mette Drastrup, Charlotte Vermehren

**Affiliations:** a Department of Clinical Pharmacology, Copenhagen University Hospital, Bispebjerg Bakke 23, DK-2400, Denmark; b PHARMA, Faculty of Health and Medical Sciences, University of Copenhagen, Universitetsparken 2, DK-2100, Denmark

**Keywords:** Deprescribing, Polypharmacy, Multimorbidity, Guidelines, Implementation

## Abstract

The first Danish symposium on evidence-based deprescribing was held in September 2019. The symposium aimed to increase the awareness of deprescribing in general, to discuss the importance of deprescribing, and, thus, a potential consensus on key issues on a national deprescribing agenda. The invited keynote speaker, Barbara Farrell, from the Bruyére Research Institute, Ottawa, Canada, presented their thorough work on deprescribing guideline development and application. The symposium consisted of two parts: Part 1 concentrated on establishing the need for deprescribing in our society. Part 2 consisted of a panel debate that put the practical application and implementation of deprescribing in perspective to the input from the audience and the structure of the Danish healthcare system. The panelists represented key stakeholders, e.g., clinical pharmacists, general practitioners, hospital doctors, Danish Health Authority representatives, health politicians concerning deprescribing in Denmark. The event allowed 145 participants to discuss the importance of implementing deprescribing in a Danish setting. This commentary highlights and discusses the major themes that characterized the symposium: “why deprescribe?”, “deprescribing research” and a theme dedicated to “problems of concern.” The emergence of these themes formed the basis for the discussion of new strategies and a proposal for a future gold standard to succeed in deprescribing.

## 1 Introduction

Polypharmacy, which is the concomitant use of multiple medications, is of great concern in Denmark and several other countries.^1^, ^2^, ^3^ Polypharmacy is associated with an increased risk of drug-related problems, e.g., adverse drug events,^4^ drug interactions,^5^ falls,^6^ hospitalizations,^7^ and mortality.^8^ A recent Danish study has shown that polypharmacy is widely spread. More than half of citizens at the age of 75 and above receive polypharmacy,^9^ and the proportion of citizens of old age is continuously increasing.^10^

In the literature, polypharmacy has most commonly been defined as using five or more medications.^11^ However, a simple numerical count of medications has limited value in practice.^12^^,^^13^ The use of multiple medications may be clinically appropriate in contrast to inappropriate polypharmacy that may increase the risk of adverse events and poor health outcomes. Hence, a shift toward the term appropriate polypharmacy has been suggested instead of using a numerical count. Distinguishing between appropriate and inappropriate polypharmacy can be difficult. Thus, deprescribing interventions can be attempted to categorize the type of polypharmacy and to decrease the risk of inappropriate polypharmacy.^2^^,^^14^

In Denmark, the concept of deprescribing is relatively new, and there is no adequate Danish word for it. The term deprescribing first appeared in the literature in 2003,^15^ but a lack of consensus of the term was established in a systematic review from 2015.^16^ Deprescribing.org defined Deprescribing as ‘*the planned and supervised process of dose reduction or stopping of medication that might be causing harm or might no longer be providing benefit’.*^16^^,^^17^ It is important to include patients, relatives, and/or caregivers in shared decision-making and to meet their preferences.^18^^,^^19^

How to deprescribe a medication can be a challenge, and until recently, no evidence-based deprescribing guidelines have been available. Such deprescribing guidelines have been developed in Canada and Australia.^17^^,^^20^, ^21^, ^22^, ^23^ The guidelines and the method for preparing the guidelines were presented at the Bruyére Evidence-Based Deprescribing Guideline Symposium in 2018.^24^

Inspired by the Canadian initiative, the Capital Region of Denmark quickly did take action to publish Danish versions of deprescribing algorithms. The Danish versions have been approved by the association of general practitioners in the Capital Region of Denmark (KAP-H) and the Medicines Committee of the Capital Region of Denmark, whose most important task is to ensure rational pharmacotherapy inthe regional hospitals, psychiatry, and primary care. The Danish deprescribing algorithms were subsequently submitted to the Danish Health Authority to ensure that the messages were according to Danish standards and recommendations.

The first Danish symposium on evidence-based deprescribing was held at Copenhagen University Hospital Bispebjerg in September 2019 by The Department of Clinical Pharmacology, The Medicine Unit. The members of the organizing committee are reflected in the author list.

The symposium aimed to increase the awareness of deprescribing in general, to meet and discuss the challenges of deprescribing among stakeholders involved in deprescribing activities from different perspectives. It will be tried to reach a consensus on key issues on a national deprescribing agenda. Finally, the symposium aimed to present and disseminate knowledge of the Canadian deprescribing guidelines and the new Danish deprescribing algorithms among Danish health care professionals.

This commentary will highlight and discuss the main themes and problems of concern identified during the symposium; not least, the commentary will discuss requests to and a proposal for a future gold standard on how to address and succeed in deprescribing for the benefit of patients, healthcare professionals, and the health care system.

## 2 Setting

### 2.1 Target group

To achieve a broad and realistic picture of the real-world polypharmacy challenges and to be able to assess the future potential of deprescribing, we invited key stakeholders as speakers, panelists, and participants, to spread awareness of deprescribing and practical execution of deprescribing activities in Denmark. The key stakeholders were represented by researchers, general practitioners, hospital doctors, clinical pharmacists, community pharmacists, an official from the Danish Health Authority, and health politicians. They shared their expertise within different aspects of deprescribing: Rational pharmacotherapy, evidence-based medicine, polypharmacy, and shared decision making across the health care system. The one-day symposium was fully booked with 145 registrants, 11 presenters, and 9 panelists.

### 2.2 What did we do?

At the beginning of the day, the participants received learning material with supportive recommendations on rational pharmacotherapy, including the Canadian and Danish deprescribing guidelines and algorithms.^17^^,^^20^, ^21^, ^22^, ^23^ In addition, an *idea card* (Appendix A) and an introduction to slido.com app was handed over. The participants were encouraged to use the idea card to write down their ideas and reflections on implementing awareness of deprescribing in their work environment. The slido.com app collected inputs and questions from the audience for the later panel debate. The completed idea cards were collected at the end of the event and used by the organizing committee to evaluate the reflections of the audience to utilize these when working to improve the deprescribing implementation in Denmark.

The event was divided into two parts (see program of the day in Appendix B). Part 1 concentrated on setting the stage for the need for deprescribing in our society, introducing the Canadian method for developing evidence-based deprescribing guidelines, the state of the art of deprescribing research, and the political visions for national medical safety, including the importance of deprescribing. At the symposium's opening, participants were welcomed by the chief medical officer at The Copenhagen University Hospital Bispebjerg and the head of the organizing committee. Part 1 was delivered by presentations followed by discussions from different health care providers perspectives, e.g., the Head of Department of Clinical Pharmacology, Copenhagen University Hospital Bispebjerg, clinical pharmacists (deprescribing pioneers) from Canada, and the head of The Health Committee at The Danish Regions that is the interest organization for the five regions in Denmark. Part 2 consisted of the panel debate, which put the practical application and implementation of deprescribing in perspective to the attitude of the audience and the structure of the Danish healthcare system. The panel debate was held in the final hours of the symposium and included all former speakers as panelists. During parts 1 and 2, notes were collected by three notetakers from the organizing committee. Notes were listed for each presentation and discussion. After the symposium, the major themes and suggestions from the participants were identified by the principles of qualitative content analysis^25^ performed based on notetakers notes from the presentations, panel debates, idea cards, and the slido.com app. A post-meeting in the organizing committee was held to consider and discuss new strategies and actions toward improving the national and international deprescribing collaboration, the evidence-based deprescribing research, and the implementation of guidelines.

## 3 Themes that characterized the discussions

The common themes and subthemes that characterized the presentations and discussions during the symposium are seen in Table 1.

**Table 1:** Themes and subthemes identified during the symposium.

Themes	Why deprescribe?	Deprescribing research	Problems of concern
Subthemes	Why is deprescribing important?	How to increase deprescribing research?	Combining treatment and deprescribing guidelines
	The worldwide interest in medication optimization	How can deprescribing research be aligned and coordinated?	Shared decision making in order to prioritize patients' preferences
	What are the benefits of deprescribing?	What are the future priorities for deprescribing research?	Coherence in health care

### 3.1 Why deprescribe?

#### 3.1.1 Why is deprescribing important?

Initially, the key speaker's presentation gave an in-depth insight into why deprescribing is important. Through her many years of work as a clinical pharmacist with polypharmacy patients, it was her experience that she had been able to increase the quality of life of these patients by a deprescribing intervention.^26^ To achieve this, it is necessary to know when and how a drug treatment can be stopped - and the consequences of stopping a drug treatment, e.g., the occurrence of a potentially harmful effect of deprescribing a drug. The keynote presentation led us through patient cases illustrating the importance of deprescribing and the considerations associated with method development for evidence-based deprescribing.

Presenters and the audience emphasized that the population is getting older, and the use of medications increases with age. More people suffer from chronic illness, multimorbidity, and polypharmacy.^9^^,^^27^ Treatments and technologies are expensive, and patients' expectations of pharmacological treatments are rising. These facts are well known but nonetheless essential to deal with when medication-related health outcomes of the population are put on the agenda. These topics are widely debated in the international literature to find a way to tackle this major global polypharmacy challenge.^28^, ^29^, ^30^ The polypharmacy challenge was widely discussed among the participants. At the symposium, one of the messages was the importance of not hesitating to increase the appropriateness of the medications of the vulnerable patient groups by deprescribing - i.e., reduction of polypharmacy.

#### 3.1.2 The worldwide interest in medication optimization

At the symposium, the worldwide interest in deprescribing was highlighted. Important initiatives exist within the World Health Organization (WHO), The National Institute for Health and Care Excellence (NICE), and national initiatives focusing on medication safety, multimorbidity, and polypharmacy. In 2017, WHO initiated the Third Global Patient Safety Challenge: “Medication without harm.” Denmark is a part of this challenge that aims to, globally, reduce the level of severe, avoidable harm related to medications by 50% over 5 years. One of the categories that the effort focuses on is polypharmacy and how addressing polypharmacy can reduce medication with harm.^31^ NICE publishes evidence-based recommendations within different areas developed by independent committees and consulted on by stakeholders.^32^ In 2017, guidance on multimorbidity and polypharmacy was published by NICE.^32^ In addition, national initiatives and networks are established in many countries with the common interest in combating the inappropriate use of medications. Hence, deprescribing is rapidly gaining momentum.

#### 3.1.3 What are the benefits of deprescribing?

Deprescribing is proposed as an intervention to reduce the harm associated with inappropriate polypharmacy.^33^^,^^34^ It is well-documented that deprescribing is an important tool in the fight against polypharmacy among older people due to an association between polypharmacy and, e.g., frailty, falls, impaired cognition, and morbidity in older people.^34^^,^^35^ It is worth noting that adding an extra medication to an already long list of medications is likely to decrease the benefit of the medications, whereas the risk of harm increases.^33^ The most consistent risk factor for adverse drug reaction is the number of medications taken by the patient.^33^^,^^36^ Hence, it is obvious that deprescribing is closely related to medication appropriateness.^14^ However, there is limited evidence available to support deprescribing as an intervention as it is still unknown to what degree deprescribing can improve medication safety.^34^

### 3.2 Deprescribing research

#### 3.2.1 How to increase deprescribing research?

Some research groups are planning to conduct randomized placebo-controlled trials testing deprescribing of standard care medication, and some with varying study-quality have already been conducted.^37^^,^^38^ It might be valuable if this type of study could be conducted independently of the pharmaceutical industry, and more funding should be given to independent research in this area.

#### 3.2.2 Can deprescribing research be aligned and coordinated?

The level of evidence in deprescribing was of great concern for the participants. There was among the assembly a future wish for establishing a solid evidence base concerning the effect of deprescribing tested in randomized placebo-controlled trials, where this is considered ethically sound. Besides this, it is important to investigate and evaluate a potential deprescribing effect on individual patients. This may specifically refer to the individual patient's ability to metabolize a given drug, e.g., in the presence of CYP450 polymorphism. The symposium called for greater national/international deprescribing collaboration, where the execution of the critical clinical trials could be coordinated and valid data on the clinical effect of deprescribing could be obtained.

#### 3.2.3 What are the future priorities for deprescribing research?

So far, clinical intervention studies are struggling to show a clear benefit in favor of the intervention. A systematic review and meta-analysis from 2016^34^ on deprescribing and its effect on mortality showed that although nonrandomized data suggested a reduction in mortality, deprescribing was not shown to alter mortality in randomized studies. However, a significant reduction in mortality was shown in randomized studies when applying individual patient-specific interventions to deprescribe.^34^ This suggests that deprescribing needs to be individualized. Mass deprescribing such as PPIs or statins may fail to capture the individual response and, therefore, not provide the guidance clinicians feel they need. Unfortunately, it is still unclear whether deprescribing interventions lead to clinically relevant effects and whether these effects are sustained.^2^^,^^34^^,^^39^

The selection of relevant outcomes was of great concern for the participants. Outcomes in deprescribing studies often include a reduction in the number of medications and an assessment of the appropriateness of drug treatment.^29^ However, such outcomes are not necessarily clinical and patient-relevant and could be included in studies as secondary outcomes. To emphasize the importance of a clinical patient-relevant effect in deprescribing, suggested *core* outcomes were: Harm (e.g., falls), clinical outcome (e.g., mortality), healthcare utilization (e.g., hospitalizations), knowledge (e.g., patient-knowledge), medication-related outcomes (e.g., number of medications) and patient-related outcomes (e.g., quality of life). These core outcomes should be combined with preference-based deprescribing decisions for individual patients, which means that a successful outcome depends on meeting the specific patient preferences or goals.^40^^,^^41^ In order to obtain knowledge about a patient's medication preferences, shared decision-making between the patient and physician can be used.^42^^,^^43^ In addition, caregivers with an in-depth knowledge of the individual patients should be involved in deprescribing decisions.

### 3.3 Problems of concern

#### 3.3.1 Combining treatment and deprescribing guidelines

A general concern among the assembly was that treatment guidelines mainly included treatment of single conditions such as diabetes, asthma, and stroke without considering any comorbidity of the individual patient and a potential prioritization of the treatment of various concomitant diseases. Hence, very few guidelines advise on when and how to deprescribe. This was cited as one reason for the high prevalence of polypharmacy. A general practitioner among the participants justified this by saying that physicians strive to do the best for their patients. The general perception among physicians is that the best thing one can do for their patients is to offer them a treatment recommended in an evidence-based guide. It is, however, important to be aware that treatment guidelines are not necessarily appropriate for the individual patient to follow, e.g., beta-blocker in an asthmatic. Deprescribing is not yet covered by the treatment guidelines, which contributes to a lack of focus among physicians on the importance of reducing inappropriate medications. This may indicate that the general practitioners need more training in appropriate prescribing and reconsidering the continued need for a drug treatment before initiating another. The prescribers need to see deprescribing as part of good prescribing practice, including medication initiation, dose titration, changing or adding medications, or deprescribing medications.^33^ When we get there, deprescribing will become a natural part of a prescriber's tasks resulting in a more thorough implementation of deprescribing in practice.

In addition, the physicians are unsure whether they comply with the Danish Agency for Patient Safety's recommendations if they do not comply with the treatment guidelines because this is currently perceived as the right way to treat patients. Especially, the young physicians were concerned about deprescribing medications among polymedicated patients. They were concerned about the lack of evidence for deprescribing in the form of, e.g., medical flare-ups and the potential negative impact on quality of life. These concerns seemed to be a great barrier to implementing deprescribing. This is problematic, as many of the real-world patients are older patients with multimorbidity and polypharmacy with a great need to deprescribe and prioritize medications.

The evidence supporting the deprescribing guideline recommendations was questioned at the symposium, as the availability and quality of evidence are often low. However, it was emphasized that prescribing guidelines often have the same issue. It was also pointed out that guidelines rarely involve elderly, multimorbid patients, making it challenging to medicate this patient group appropriately.

Hence, combining prescribing and deprescribing guidelines in the future was a request from both the developers of the deprescribing guidelines and the health care professionals present at the symposium. In addition, the guidelines should preferably include older people and patients with comorbidities to support physicians in prioritizing medication and achieving an appropriate prescribing pattern for these patients.^44^ In clinical trials, older patients and patients with polypharmacy are poorly represented, and these patients are often more prone to adverse effects due to comorbidities and concomitant medications. Age-related physiological changes can affect the pharmacokinetics and pharmacodynamics of the drug. Therefore, poor representation of older people in clinical trials leads to inadequate evidence and knowledge regarding drug therapy among the elderly. The developers of the national prescribing guidelines should take up these issues, i.e., the Danish Health Authority.

#### 3.3.2 Shared decision making in order to prioritize patients' preferences

During the discussions, it became clear that shared decision-making should be a mandatory part of a deprescribing process. Studies indicate trends to an increased quality of life, adherence, and commitment to patients' decisions when they are part of the decision-making process.^45^^,^^46^ Patients often have a strong sense of what is valuable to them. For a shared decision, it is important for the healthcare professional to know the patient's preferences and priorities and for the patient to be informed about the benefits and harms associated with drug treatments. This should end up with a shared decision to receive the best possible treatment in agreement with both the physician and the patient.^42^^,^^43^ This requires establishing a safe relationship between patient and physician so that the patient will honestly and without reservation tell about their preferences and any potential resistance to the various drugs. At the symposium, decision tools were presented. Tools that could support both the patients and their general practitioners in the right medical decision in terms of their individual preferences, and that could guide their deprescribing process to find out which medication to stop first. These tools include, for example, information leaflets for patients on why sleeping pills should be avoided and replaced by non-pharmacological initiatives against insomnia (Appendix C and D). The tools also included national guidance for general practitioners on approaching a medication review for an elderly polypharmacy patient (Appendix E) and a national deprescribing list indicating which drugs should be deprescribed in elderly patients and how a deprescription can be performed in practice (Appendix F). However, in this area, we lack evidence that can guide us on how we practically can identify the preferences of polypharmacy patients and how to include these in the shared decision processes.

#### 3.3.3 Coherence in health care

##### 3.3.3.1 Silo medicine

Silo medicine^47^ was discussed as a possible explanation for the large extent of polypharmacy among the population in Denmark and most other countries. The concept of silo medicine occurs when information about patient care is not sufficiently exchanged between health care professionals in different health care sectors or between different care specialists, e.g., general practitioners, psychiatrists, cardiologists. For patients treated by different physicians, there may be an overlap in their treatment, resulting in overtreatment. Coordination of a polypharmacy patient's overall and disease-specific medications is complex and poses a challenge to healthcare professionals. It has generally been found that there is a lack of clarity about clinical responsibility and poor communication between health care professionals.^48^ Consequently, the health care system is so fragmented that nobody sees the full picture of the patients.

The silo mentality is believed to reduce the efficiency of overall patient care. Unfortunately, it is difficult to avoid silo medicine due to the structure of the healthcare system today, where general practitioners are supposed to see the full picture of a patient's treatment – but this is a difficult and time-consuming task that is not always possible in practice, especially, for patients with polypharmacy and multimorbidity. The organization of the health care system may have an impact on the medication management of multimorbid patients. Thus, optimizing communication between patients, general practice, hospitals, acute care facilities, and nursing homes must be well established and easily accessible. It can be imagined that a shift to a holistic healthcare system will break down silos between sectors and support higher quality collaborative care.

##### 3.3.3.2 Organizing the health care system in a way where there is a constant focus on rational prescribing and deprescribing

The organization of the health care system was only superficially discussed, although there was apparently a general agreement among the participants that the structuring of the health care system has a great influence on the increasing number of polypharmacy patients in Denmark. Structural changes connecting prescribing and deprescribing with the emphasis on encouraging more thorough medication monitoring and reevaluation, and deprescribing was suggested.

The communication between health care professionals is expected to be significantly improved by optimizing electronic health records. These could advantageously contain automatic system-generated deprescribing recommendations based on the individual patient's medication list, diagnoses, patient adherence, and redeemed medications at the pharmacy.

Other important focus areas concerning optimizing rational prescribing and encouraging deprescribing were mentioned. Participants agreed that deprescribing is about better prescribing processes, which requires targeted training of health care professionals in prescribing medication appropriately. For this training, the prescribing/deprescribing guidance tools, ‘Beers criteria for potentially inappropriate medication use in older adults’ and ‘STOP/START criteria for potentially inappropriate prescribing in older people’ published by American Geriatrics Society 2019 and 2015, respectively, can be recommended to use.^49^^,^^50^ In addition, a focus should be on making use of multidisciplinary teams in the healthcare system and unifying the different medical record systems to contain the same patient information, e.g., in general practice, hospitals, pharmacies. Interprofessional education among, e.g., pharmacists, physicians, and nurses, was highlighted for the professions to build clinical relationships and recognize the roles of other health care professions, which is supposed to lead to increased patient safe medication practice.^51^ However, the most important thing is to involve the patient in all matters concerning their medication.

At the symposium, the national health political visions for deprescribing were presented. The political visions supported the views presented at the symposium: Patients should only receive the medication needed, there should be more focus on reducing polypharmacy, attention to that medications not always are the right solution for the patients, doctors should be educated in rational pharmacotherapy, and finally, we need independent research to support clinical decision making.

## 4 Conclusion: in the future, what would the gold standard be?

Based on the themes that emerged and other outcomes from the symposium, a future gold standard was proposed to achieve a shared vision to succeed with deprescribing.

**Box 1. The future gold standard of deprescribing proposed based on outcomes from the symposium.**

The future gold standard to succeed in deprescribing

In general

● Do not hesitate to consider deprescribing medications of vulnerable patient groups.
● Ensure focus on solid research that can document the effect of deprescribing
● Consider deprescribing based on the practice of evidence-based medicine^52^

Combine treatment and deprescribing guidelines

● Define clear guidelines for initiation, monitoring, reevaluation, and deprescribing medicine
● Include considerations on prioritizing medications for multimorbid polypharmacy patients
● Encourage shared decision making and awareness of patient preferences
● Preferably base the guidelines on research results obtained by RCTs or external clinical evidence^52^

Coherence of the health care system

● Avoid silo medicine by increasing communication between sectors and specialties
● Interprofessional education of health care professionals in rational pharmacotherapy
● Engage patients through shared decision making

Contribute to evidence generation in clinical practice

● Follow up on patients who have received deprescribing interventions
● Note and document the observed deprescribing effects
● Collect these experiences to build an evidence base for clinical practice

Alt-text: Unlabelled Box

With this symposium, attention has been drawn to the importance of evidence-based deprescribing in the health care system. The first step in a hopefully future national and international collaboration concerning deprescribing between health care stakeholders has now been taken.

Until now (2021), we believe that the symposium has already created attention to – and had a certain impact on - the focus on deprescribing.

During the symposium, the Danish Health Authority encouraged participants to seek funding from the authority to develop deprescribing guidelines and has now funded the development of a national clinical guideline regarding deprescribing of- and continued treatment with inhaled steroid in patients with chronic obstructive pulmonary disease (appendix G). The guideline was published in April 2021 as the first Danish deprescribing guideline. Under the auspices of the Danish Health Authority, an initiative has also been taken to translate a national ‘deprescribing list’ into English to use this in the scientific literature and thereby contribute to international awareness of deprescribing.

In addition, it can be mentioned that the Danish deprescribing algorithms are now widely used in the Capital Region of Denmark. Several publications and interviews in press media within the area and pilot studies in two Danish regions dealing with the testing of the integration of pharmaceutical competencies in general practice regarding medication review and deprescribing among polypharmacy patients.

## Funding

Funding for the planning and holding of the symposium was provided by the Department of Clinical Pharmacology, Copenhagen University Hospital.

## Declaration of Competing Interest

None.

## Appendix A Supplementary data

Supplementary material 1 Appendix A The idea-card. Image 1

Supplementary material 2 Appendix B The program agenda. Image 2

Supplementary material 3 Appendix C Good advice if you have difficulty sleeping. Image 3

Supplementary material 4 Appendix D Patient information: Drop sleeping pills and sedatives. Read why and how. Image 4

Supplementary material 5 Appendix E Medication review in practice. Image 5

Supplementary material 6 Appendix F Deprescribing list 2021. Danish Health Authority. Image 6

Supplementary material 7 Appendix G National clinical guideline regarding deprescribing and continued treatment with inhaled steroid in patients with COPD. Image 7
