# Supplementary material for: Deprescribing: What is the gold standard? Themes that characterized the discussions at the first Danish symposium on evidence-based deprescribing
Source: Explor Res Clin Soc Pharm. 2022 Jan 7;5:100102. doi: 10.1016/j.rcsop.2022.100102 (PMC9030658; doi:10.1016/j.rcsop.2022.100102)
Supplement: Supplementary file 1 — Supplementary material 1 Appendix A The idea-card. [file mmc1.pdf]

How can you implement deprescribing in your organization?

Please write your idea(s) here:

In the end of the day, please return this slip in the box marked ”Idea Box”
