# Supplementary material for: Deprescribing: What is the gold standard? Themes that characterized the discussions at the first Danish symposium on evidence-based deprescribing
Source: Explor Res Clin Soc Pharm. 2022 Jan 7;5:100102. doi: 10.1016/j.rcsop.2022.100102 (PMC9030658; doi:10.1016/j.rcsop.2022.100102)
Supplement: Supplementary file 2 — Supplementary material 2 Appendix B The program agenda. [file mmc2.pdf]

Speakers

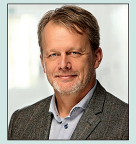

Kristian Antonsen
MD, EDIC, MHM. Specialist in Anesthesiology and Intensive Care Medicine.
Chief Medical Officer at Bispebjerg and Frederiksberg Hospital.

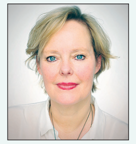

Charlotte Vermehren
PhD, MSc. Pharm. Head of Medicine Unit, Department of Clinical Pharmacy, Bispebjerg and Frederiksberg Hospital. Associate Professor at The University of Copenhagen.
Vice chair of Section for Clinical Pharmacy, Danish Pharmaceutical Society. Charlotte focuses on strengthening rational pharmacotherapy in the primary care sector - especially among GPs and across sectors of The Capital Region.

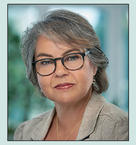

Hanne Rolighed Christensen
PhD MD, Head of Department of Clinical Pharmacology, Bispebjeg and Frederiksberg Hospital. Member of the National Council of Medicine. Vice chairman of the Drug Committee, Capital Region. Chairman of the association of the 5 Regional Drug Committee. Medical specialist in, Clinical pharmacology, Internal medicine, Pulmonary medicine.

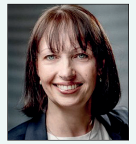

Karin Friis Bach
Regional politician from the Capital Region of Denmark since 2014. Her main political focus is on health politics. She is now the 2nd vicechair of the regional counsel and head of the health committee in the national association of Danish Regions. She is also the chairwoman of the Danish Society for Patient Safety. Karin Friis Bach holds a master of science in pharmacy and has worked in her professional life within the pharmaceutical field for more than 20 years.

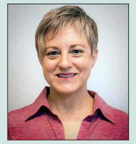

Barbara Farrell
Dr., Scientist with the Bruyère Research Institute (Ottawa), Assistant Professor with the Department of Family Medicine, University of Ottawa and Adjunct Assistant Professor with the School of Pharmacy, University of Waterloo. She is a clinical pharmacist in the Bruyère Geriatric Day Hospital and leads the Deprescribing Guidelines Research team at the Bruyère Research Institute. In 2011, she received the Canadian Pharmacist of the Year award from the Canadian Pharmacists Association and in 2018, the Exceptional Achievement in Research and Academia award from the Ontario Pharmacists Association. She is a founding member of the Canadian Deprescribing Network.

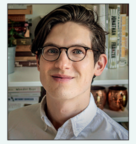

Wade Thompson
BScPhm, PharmD, MSc. Wade is a pharmacist and researcher, currently doing his PhD at the University of Southern Denmark. He has worked as a clinical pharmacist in primary care clinics and in nursing homes. His research focuses on deprescribing and shared decision-making around medication use.

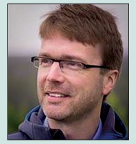

Torsten Risør
Scientist, MD, Norwegian Centre for E-health Research. Torsten is a family physician and senior researcher from Tromsø, Norway, with a particular interest in clinical decision-making in primary care: How are decisions actually made in the complex interplay between patient, health professionals, tools and other object in the clinical space, organizational pathways and structures, cultural norms and habits? How can we conceptualize that complexity in a way that allow us to interact with it in structured ways while remaining open to its uncertainty and unpredictability?

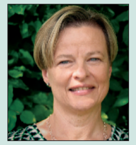

Gitte Krogh Madsen
MD. GP with a special interest in rational pharmacotherapy and deprescribing. She is employed by Region Zealand as a consultant doing medical reviews, teaching, developing local guidelines and providing feedback to fellow GPs on their prescriptions. She also teaches deprescribing to junior doctors.

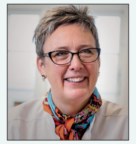

Anne Jung
Specialist in Internal Medicine, Geriatrics 2005, Chief Physician 2007. Responsible for Geriatric Ambulatory at Amager Hospital (2007-2014) and the Pain Clinic at Holbæk Hospital (2014-2016). Now she is the responsible Chief Physician and Organizational Manager of the Medical Joint Ambulatory (Medicinsk Fælles Ambulatorium), including the Unit for Interdisciplinary Investigation and Treatment. Furthermore, she teaches physicians and pharmacists in multi-disease and polypharmacy.

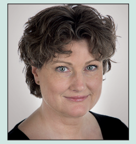

Dorthe Vilstrup Tomsen
Msc. Pharm, IA, Head of psychiatric Centre Amager. As a pharmacist, Dorthe has led the work in creating clinical solutions within the field of medication safety for the last decade. Based on the conditions at a busy ward, she has created sustainable results in both medication reconciliation, medication review, antibiotic stewardship and latest, a program for ambulatory deprescribing.

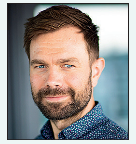

Simon Tarp
PhD in evidence-based medicine, Msc. Pharm. Evidence method expert at the Danish Health Authority. Responsible for evidence method behind the national clinical guidelines and other products with national clinical recommendations. Representing the Danish Health Authority in the national group responsible for the Danish list of deprescribing recommendations.

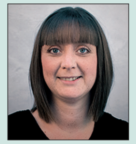

Rikke Nørgaard Hansen
Msc. Pharm. Head of research and development at Pharmakon, Danish College of Pharmacy Practice. She is a pharmacist with a master in clinical pharmacy and is involved in research projects and developing services in community pharmacy practice.

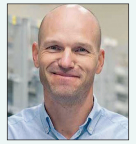

Thomas Croft Buck
Msc. Pharm. Chief pharmacist in a Drugstore for the last 3 years.
Earlier Chief pharmacist of the clinical pharmacy department in a University hospital for 9 years, initiating studies in the field of clinical pharmacy. Pharmaceutical consultant for the government for 3 years working with rational pharmacotherapy with GP's.
Working with clinical pharmacy as a hospital pharmacist for 5 years.

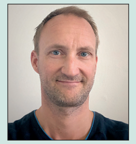

Mikkel Bring Christensen
Specialist in Clinical Pharmacology, Chief Physician (Overlæge), PhD, Associate Professor.
Research focus on clinical value of medication reviews.

DETAILED PROGRAM

Evidence-based Deprescribing in Denmark

September 23rd, 2019, 08:30-16:30

Funded by the Medicine Unit, Department of Clinical Pharmacology, Bispebjerg and Frederiksberg Hospital

Bispebjerg Hospital, Uddannelsescentret, Entrance 50, 2400 Copenhagen NV

Region Hovedstaden

Welcome

Welcome to a day focusing on why and how evidence-based deprescribing guidelines are developed. Perspectives regarding relevance as well as barriers for implementing these guidelines in Denmark will be discussed among physicians and pharmacists, researchers, politicians and the Danish Health Authority.

Use of evidence-based treatment guidelines support initiation and maintenance of pharmacological treatment. However, many receive medication that is no longer needed and deprescribing of well-intentioned medication may be relevant. This could for instance be due to unacceptable side effects or lack of effect. Deprescribing can be difficult and until recently, very few evidence-based deprescribing tools have been available.

A specific method for developing evidence-based deprescribing guidelines have now been evolved in Canada and Australia. Our keynote speaker for today, Dr. Barbara Farrell, is one of the initiators behind these guidelines, and she will introduce us to the work behind and the effects of implementation found through her research.

To make sure we address your most important questions at today's symposium, we will be using a tool that allows you to submit your questions and express your opinion. Questions will be collected and used for today's panel discussion. To join:

1. Simply take out your smartphone and open your browser
2. Go to **Slido.com** and enter the event code **#DEPRESCRIBE**
3. You can now ask questions and upvote the best ones.

Of course, also oral questions are welcome all through the day.

Also please fill in your suggestions, on the "questions-card" and hand it in the box in the hallway marked "Idea box".

We look forward to a day presenting interesting perspectives and with enriching discussions.

Welcome!
Organizing committee

Medicine Unit – about us

We work by promoting and enhancing the rational pharmacotherapy in the primary sector and between sectors in our region

Medicine Unit (Medicinfunktionen) is a part of the Department of Clinical Pharmacology at Bispebjerg and Frederiksberg Hospital in the Capital Region of Denmark. We work closely with the primary care and especially general practice.

We develop drug recommendations, methods and tools that can promote rational use of medicine to support general practitioners' knowledge and awareness of rational pharmacotherapy.

The overall goal is to enhance the interdisciplinary, intersectoral and national collaboration for a more rational and coherent treatment for the patients in the Capital Region of Denmark.

The Unit's research aims to increase quality of medicine use for example in our medication review studies and our deprescribing projects.

For more information, please visit our Danish website **www.medicinfunktionen.dk**

Organizing committee

Charlotte Vermehren,
PhD, MSc. Pharm, Head of Medicine Unit, Department of Clinical Pharmacology

Dagmar Abelone Dalin,
MSc. Pharm, Medication consultant, Medicine Unit, Department of Clinical Pharmacology

Anne Mette Drastrup,
MSc. Pharm, Medication consultant, Medicine Unit, Department of Clinical Pharmacology

Lykke Ida Kaas Oldenburg,
MSc. Pharm, PhD candidate, Medicine Unit, Department of Clinical Pharmacology

Program

**08:30**	**REGISTRATION, COFFEE AND CROISSANTS**	
09:00	**WELCOME**<br>Kristian Antonsen	**CHAIRMAN:**<br>Charlotte Vermehren
09:10	**INTRODUCTION TO THE DAY**<br>Charlotte Vermehren	
09:15	**WHY DEPRESCRIBING**<br>Hanne Rolighed Christensen	
09:35	**DEPRESCRIBING: POLITICAL VISIONS**<br>Karin Friis Bach	
09:50	**WHAT IS DEPRESCRIBING GUIDELINES?**<br>Barbara Farrell	
**10:35**	**FRUIT AND COFFEE BREAK**	
11:00	**THE METHOD**<br>Barbara Farrell	**CHAIRMAN:**<br>Gitte Krogh Madsen
11:30	**IMPLEMENTATION AND EVALUATION**<br>Barbara Farrell	
**12:15**	**LUNCH BREAK**	
13:15	**DEPRESCRING RESEARCH**<br>Wade Thompson	**CHAIRMAN:**<br>Mikkel Bring Christensen
13:45	**HEALTH CARE PROVIDERS PERSPECTIVES - IMPLEMENTATION IN DENMARK**<br>• Perspective on clinical decision making: Torsten Risør<br>• Primary Sector: Gitte Krogh Madsen<br>• Secondary Sector: Anne Jung<br>• Clinical Pharmacy: Dorthe Vilstrup Tomsen<br>• Danish Health Authority: Simon Tarp<br>• Community Pharmacy: Thomas Croft Buck and Rikke Nørgaard Hansen	
**15:15**	**CAKE AND COFFEE BREAK**	
15:30	**PANEL DISCUSSION - IMPLEMENTATION OF DEPRESCRIBING GUIDELINES IN DENMARK**<br>• Barbara Farrell<br>• Wade Thompson<br>• Torsten Risør<br>• Gitte Krogh Madsen<br>• Anne Jung<br>• Dorthe Vilstrup Tomsen<br>• Simon Tarp<br>• Thomas Croft Buck<br>• Mikkel Bring Christensen	**MODERATOR:**<br>Hanne Rolighed Christensen and Charlotte Vermehren
16:25	**CLOSING REMARKS**<br>Charlotte Vermehren	
**16:30**	**REFRESHMENTS AND NETWORKING**	
