# Supplementary material for: Deprescribing: What is the gold standard? Themes that characterized the discussions at the first Danish symposium on evidence-based deprescribing
Source: Explor Res Clin Soc Pharm. 2022 Jan 7;5:100102. doi: 10.1016/j.rcsop.2022.100102 (PMC9030658; doi:10.1016/j.rcsop.2022.100102)
Supplement: Supplementary file 3 — Supplementary material 3 Appendix C Good advice if you have difficulty sleeping. [file mmc3.pdf]

Gode råd,
hvis du har svært ved at sove ...

Prøv, hvilke søvnråd der virker bedst for dig.

Normal søvn

- Voksne har generelt behov for at sove 6-9 timer i døgnet. Det antal timer, du sover, falder med alderen.
- Det er helt normalt at vågne i løbet af natten og at have søvnbesvær i kortere perioder, fx ved stress eller bekymringer.
- Tal med din læge, hvis du har vedvarende fysiske eller psykiske problemer, der holder dig vågen.

Gode råd om mad, drikke og fysisk aktivitet

Undgå koffein og alkohol sidst på dagen

- Opkvikkende drikke som kaffe, te, energidrikke eller cola kan gøre det sværere at falde i søvn og give kortere søvn.
- Alkohol kan måske gøre det lettere at falde i søvn, men kan også give urolig søvn, mareridt og afbrudt søvn.

Undgå rygning

- Undgå at ryge - også passiv rygning. Det kan give kortere søvn og andre søvnforstyrrelser.

Gå ikke sulten eller overmæt i seng

- Undgå store mængder energi- og fedtrig mad før sengetid. Det kan afkorte søvnens længde. Du skal dog heller ikke gå sulten i seng.
- Spis en sund og varieret kost med bl.a. frugt, grøntsager og fuldkorn. Visse fødevarer fx mælk, bananer og mandler kan være med til at øge søvnlængden.

Vær fysisk aktiv

- Dyrk regelmæssig motion, og vær fysisk aktiv hver dag. Det fremmer en naturlig træthed. Nogle skal dog ikke træne for tæt på sengetid.

Gode vaner omkring sengetid

Skab faste rammer om din søvn

- Gå i seng og stå op til faste tider hver dag – også selvom du først er faldet sent i søvn. En regelmæssig døgnrytme kan hjælpe dig til en bedre søvn.
- Sov ikke om dagen, selvom du er søvnig. Søvnigheden går som regel over efter et stykke tid, specielt hvis du er fysisk aktiv.
- Undgå at arbejde og at besvare e-mails, sms osv. i soveværelset. Din hjerne skal forbinde sengen med søvn.
- Forlad soveværelset, hvis du ikke kan falde i søvn, fx efter 30 min. Foretag dig noget stilfærdigt (fx læs i en bog, hør en lydbog eller lyt til stille musik). Gå i seng igen, når du bliver søvnig.

Soveværelset bør være roligt, mørkt og ikke for varmt

- Undgå forstyrrelser i soveværelset. Læg fx mobiltelefonen udenfor soveværelset, eller slå lyd og vibration fra.
- Brug evt. maske over øjnene og ørepropper til at lukke lys og støj ude. Overvej mørklægningsgardiner.
- Luft ud. Optimal temperatur er for de fleste 18-21 grader.

Slap af inden sengetid, og undgå lys fra skærme

- Lær dig en afspændingsteknik, og brug den, før du lægger dig til at sove, eller hvis du vågner om natten. Det mindsker spændinger, der modvirker søvn. Træk vejret dybt og roligt, og lyt evt. til beroligende musik.
- Tag evt. et varmt bad.
- Undgå lys fra skærme (computer, tablet, mobiltelefon og tv) helst flere timer før sengetid. Lyset, specielt blåt lys, kan påvirke døgnrytmen. På mange skærme kan lysintensitet og farve dog indstilles (evt. via apps).

Løs ikke dagens eller morgendagens bekymringer i sengen

- Skriv problemerne ned flere timer før sengetid, angiv mulige måder at håndtere dem på, og læg problemerne til side.
- Hav evt. blok og blyant ved sengen, så du kan skrive de tanker eller bekymringer ned, der holder dig vågen, efter du er gået i seng. Beslut dig for først at gøre noget ved dem dagen efter.

Frygt ikke søvnløshed

- Undgå at bekymre dig for meget om din søvn. Vi er ret robuste over for søvnmangel, og bekymringen kan i sig selv være med til at holde dig vågen.
- Mange bliver overraskede over, hvor længe de faktisk sover. Du kan lave en søvndagbog. Skriv ned, hvornår du gik i seng, hvornår du ca. faldt i søvn, hvor mange gange du vågnede om natten, hvornår du vågnede om morgenen, og hvornår du stod op. Nogle ure og apps til mobilen kan også registrere fx søvnkvalitet og -længde.
- Læg mærke til, hvad der fremmer en god nats søvn for dig, og hvad der virker negativt.

Undgå at bruge sovemedicin. Det løser ikke dine søvnproblemer. Søvnbesvær kan oftest afhjælpes ved, at du laver om på dine vaner.

Sovemedicin

- Undgå at bruge sovemedicin. Det kan give afhængighed og ved langvarig brug forstyrre din søvn. Det kan også påvirke din hukommelse, koncentration og reaktionsevne.
- Tag derfor aldrig sovemedicin rutinemæssigt hver dag. Brug højst sovemedicin få nætter ad gangen.
- Tag ikke sovemedicin på forskud. Tag det kun, hvis du efter nogen tid ikke er faldet i søvn - og hvis ovenstående råd ikke virker.

September 2016

Primær reference: Jennum et al. Søvn og Sundhed, København: Vidensråd for Forebyggelse, 2015
