# Supplementary material for: Deprescribing: What is the gold standard? Themes that characterized the discussions at the first Danish symposium on evidence-based deprescribing
Source: Explor Res Clin Soc Pharm. 2022 Jan 7;5:100102. doi: 10.1016/j.rcsop.2022.100102 (PMC9030658; doi:10.1016/j.rcsop.2022.100102)
Supplement: Supplementary file 4 — Supplementary material 4 Appendix D Patient information: Drop sleeping pills and sedatives. Read why and how. [file mmc4.pdf]

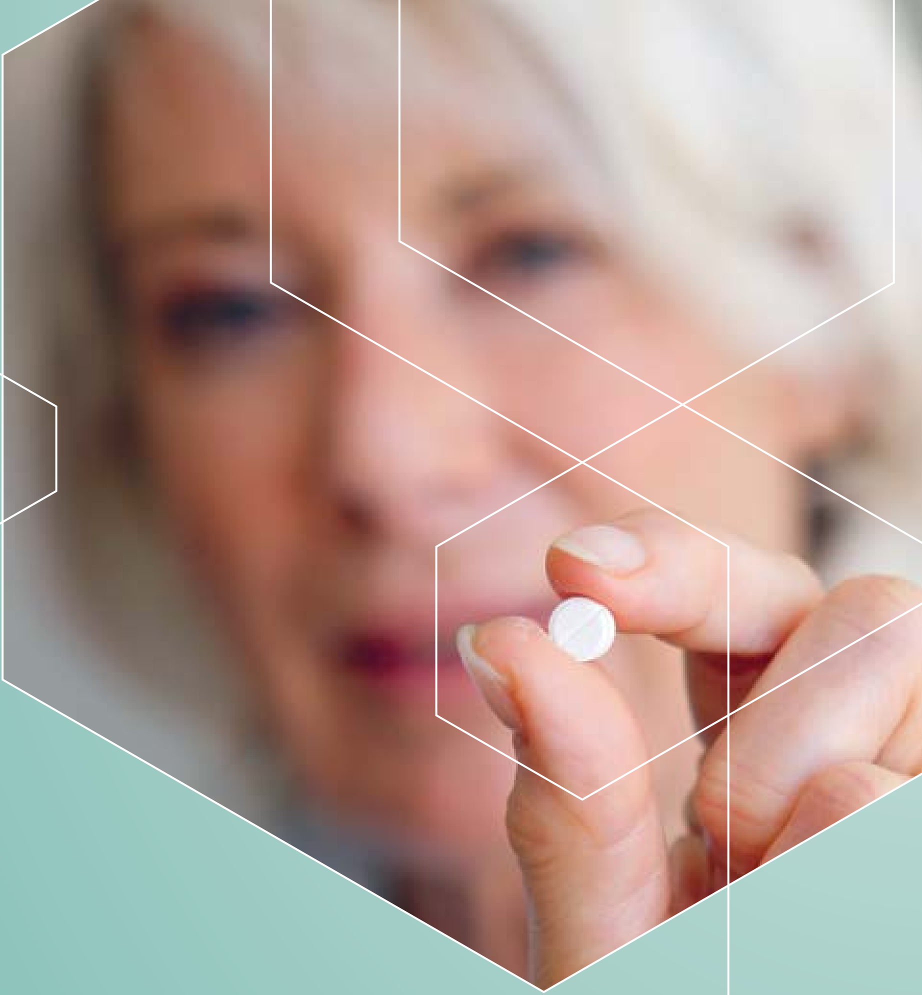

Information til patienter

Drop sovemedicin og beroligende medicin

– læs her hvorfor og hvordan

3 Gode Grunde

til at droppe sovemedicin og beroligende medicin

Medicinen er vanedannende og virkningen aftager efter kort tid

1. Sovemedicin og beroligende medicin har mange bivirkninger, som du helt kan undgå, hvis du ophører med medicinen. Bivirkningerne kan være:
   - Svimmelhed og døsighed, der kan medføre, at du falder
   - Forvirring, hukommelses- og koncentrationsbesvær
   - Svedeture, angst, uro og søvnforstyrrelser
   - Nedsat reaktionsevne, som fx kan gøre det farligt at køre bil.
2. Patienter, der er kommet ud af et længerevarende forbrug, kan ofte fortælle, at de har fået mere energi. De oplever at kunne tænke klarere og interessere sig mere for, hvad der sker omkring dem.
3. Din evne og ret til at køre bil kan bevares/genvindes, hvis du ikke tager sovemedicin og beroligende medicin.

Det tager kun få uger at blive afhængig!

Hvordan stoppes behandlingen?

Et brat ophør med medicinen kan give abstinenser som fx søvnløshed, uro og angst. Abstinenserne er ikke et tegn på, at behandlingen med sovemedicin eller beroligende medicin er nødvendig og ikke kan undværes. De er et tegn på, at kroppen igen skal vænne sig til at undvære medicinen.

Det er derfor vigtigt, at du og din læge i fællesskab lægger en plan for din udtrapning.

Varighed af udtrapning afhænger af, hvor længe du har taget medicinen, og hvilken medicin du bruger.

Har du taget medicinen i 4-6 uger, kan udtrapning ofte foregå over få dage.

Har du taget medicinen i længere tid, vil udtrapning oftest foregå meget langsomt.

De fleste kan fortsætte med den medicin, de er i behandling med, under udtrapningen. Nogle kan dog have gavn af at skifte over på en anden medicin, inden udtrapning begyndes.

Ophør med sovemedicin, som udskilles hurtig fra kroppen, giver sjældent egentlige abstinenser, men nogle uger med søvnproblemer kan forventes.

Langsom udtrapning

- Den langsomme udtrapning foregår typisk ved, at man fjerner lidt af din medicin med 1-2 ugers interval, så kroppen vænner sig til at få mindre og mindre medicin.
- Du kan undervejs opleve abstinenser, men de vil aftage med tiden. Hvis du oplever uudholdelige abstinenser, er det vigtigt, at du kontakter din læge og ikke selv justerer i mængden af din medicin.
- Et vigtigt princip i udtrapningen er, at man ikke øger mængden af medicin igen, hvis der opstår abstinenser. I stedet skal man vente med at trappe yderligere ned, således at kroppen kan nå at vænne sig til den nye dosis.

Få støtte fra dem der er nærmest

Det er vigtigt med opbakning fra venner og familie til at gennemføre udtrapningen. Din læge kan også støtte dig og give dig gode råd.

Det vigtigste er, at du vil det, er tålmodig og tror på, at det kan lade sig gøre – så kan du!

Hvad kan jeg selv gøre?

Det er individuelt, hvad der hjælper bedst. Vær bl.a. opmærksom på følgende:

Begræns alkoholindtagelse i udtrapnings-perioden. Nogle oplever, at indtagelse af alkohol forværrer deres abstinenser. Der er også risiko for, at man øger sit alkoholforbrug og kan ende med en ny afhængighed.

Hav fokus på ting, der kan aflede dine tanker, hvis du oplever abstinenser.

Det kan fx være:

- at gå, løbe, danse eller lave anden fysisk aktivitet, der passer ind i dit liv
- at tage et bad
- at være sammen med andre
- at lave afspændings- og åndedræts-øvelser eller mindfulness bl. a. ved angst eller uro.

En sund livsstil er med til at give dig energi, så du har mere overskud til udtrapningen. Spis sundt og varieret, og sørg for at få re-gelmæssige måltider. Begræns dit indtag af sukker og kaffe.

Få regelmæssig søvn. Kroppen skal genfinde en god søvnrytme. Du kan tale med din læge om at få materialet "Gode råd, hvis du har svært ved at sove".

Før evt. dagbog, så du kan følge dine frem-skridt.

Efter endt udtrapning kan der forekomme senabstinenser, som med tiden fortager sig. Det er derfor en god idé, at du fortsætter den gode livsstil også efter udtrapningen.

Du kan tale med din læge om de problemer, du oplever i forbindelse med din udtrapning.

Find yderligere råd og støtte

Benzorådgivningen giver gratis anonym rådgivning om medicinafhængighed (benzodiazepiner). Mulighed for tilmelding til støttegruppe.

Telefon 70 26 25 10
Mandag – torsdag kl. 18-20
Onsdag kl. 9-11

E-mail: benzo@sind.dk
www.benzo.dk
Find også BenzoRådgivningen på facebook

Udtrapningsplan

Præparatnavn:

	Dato	Doseringstidspunkter / Dosering				Bemærkning
Trin 1						
Trin 2						
Trin 3						
Trin 4						
Trin 5						
Trin 6						
Trin 7						
Trin 8						
Trin 9						

Noter

fx tid for lægeaftaler, spørgsmål til næste lægekonsultation mm.

Januar 2017

REGION H
Klinisk Farmakologisk Afdeling
Medicinfunktionen

KAPH
Kvalitet i Almen Praksis
i Hovedstaden
