# Supplementary material for: Deprescribing: What is the gold standard? Themes that characterized the discussions at the first Danish symposium on evidence-based deprescribing
Source: Explor Res Clin Soc Pharm. 2022 Jan 7;5:100102. doi: 10.1016/j.rcsop.2022.100102 (PMC9030658; doi:10.1016/j.rcsop.2022.100102)
Supplement: Supplementary file 5 — Supplementary material 5 Appendix E Medication review in practice. [file mmc5.pdf]

Medicingennemgang i praksis

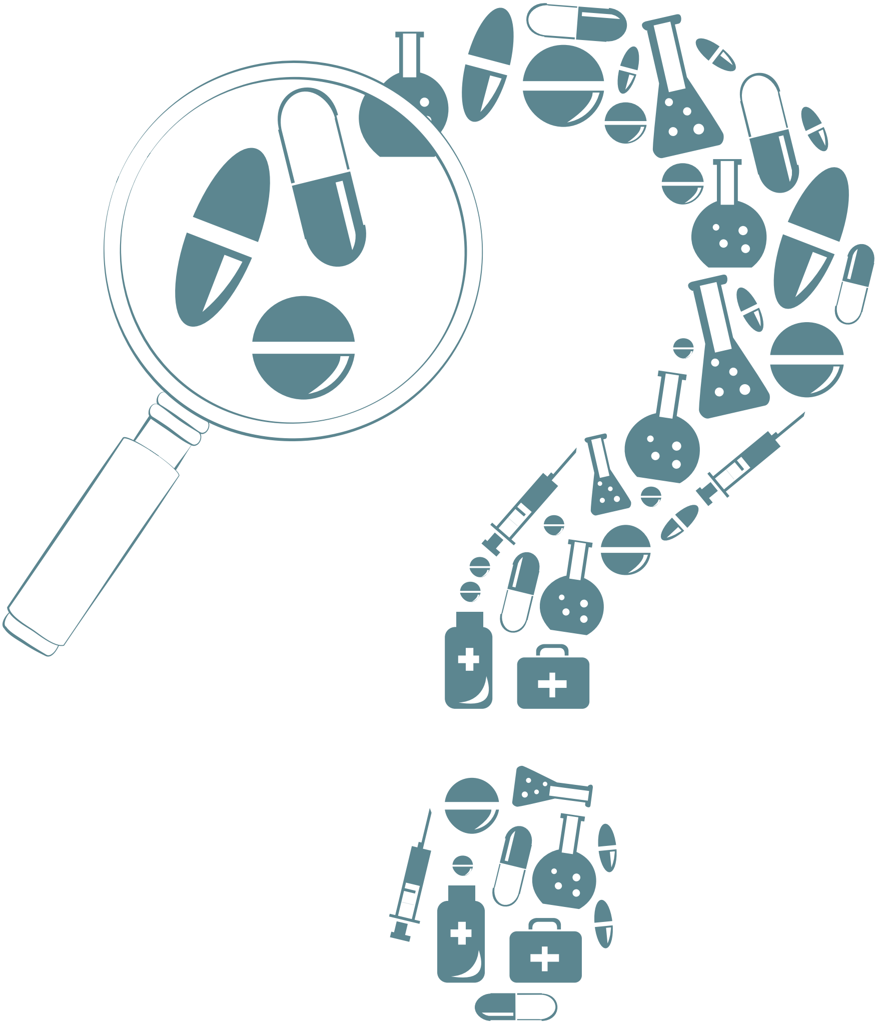

INDHOLDSFORTEGNELSE

Indledning 3

Begrebsafklaring 4

Medicingennemgang i praksis 5

Hvordan finder du patienterne? 7

Inddragelse af praksispersonalet 9

Målsætning og evaluering 9

Honorering for medicingennemgange 9

Redskaber til medicingennemgange 10

Regionale lægemiddelkonsulenter 11

Folderen er udarbejdet af regionernes lægemiddelkonsulenter og IRF i Sundhedsstyrelsen.

INDLEDNING

Der er gode grunde til at gennemgå dine patienters samlede medicin ved en struktureret medicingennemgang. Mange patienter i almen praksis lider af flere sygdomme og kan derfor blive behandlet med flere – måske mange – lægemidler. Herudover kan de have et forbrug af håndkøbs- og naturlægemidler, mineraler og kosttilskud, som lægen ikke kender til. Indikationer ændrer sig, nye kan opstå, og andre er måske ikke længere aktuelle. Kombinationen af flere lægemidler og naturlægemidler kan give anledning til interaktioner og bivirkninger.

Målet med denne folder er at opfordre og understøtte den enkelte praktiserende læge til systematisk og kritisk at gennemgå relevante patienters samlede lægemiddelbehandling.

I folderen gennemgås centrale begreber og bærende principper i en medicingennemgang samt opgaver forud for og efter en medicingennemgang. Før en medicingennemgang udføres, skal der laves en medicinanamnese, som omfatter alle former for medicin, som patienten anvender. Herefter kan en egentlig medicingennemgang udføres. Det er god skik at afslutte medicingennemgangen med en ajourføring i FMK, hvor lægen signalerer til den næste bruger, at FMK afspejler patientens aktuelle lægemiddelbehandling.

BEGREBSAFKLARING

Medicinanamnese

Ved medicinanamnesen udarbejdes en liste over en patients aktuelle lægemiddelbehandling ved at sammenholde oplysninger fra FMK, patient og evt. pårørende/plejepersonale. Medicinlisten kan også omfatte håndkøbsmedicin, vitaminer og naturlægemidler. Medicinanamnese er nødvendigt inden medicingennemgang.

Medicingennemgang

Medicingennemgang er en struktureret og kritisk gennemgang af patientens samlede lægemiddelbehandling. Formålet er at optimere lægemiddelbehandlingen for den enkelte patient. Forud for en medicingennemgang foretages en medicinanamnese.

Ajourføring

Ajourføring er en funktionalitet i FMK og signalerer til den næste bruger, at FMK afspejler patientens aktuelle lægemiddelbehandling og ikke indeholder åbenlyse fejl. I almen praksis anbefales ajourføring ved henvisninger, årskontrol, opsøgende og opfølgende hjemmebesøg samt ved ændringer i patientens medicinske behandling (ny ordination, seponering, dosisændring). Der er intet særskilt juridisk ansvar forbundet med at ajourføre FMK.

Ikke min ordination – mit ansvar?

Alment praktiserende læger har ansvar for egne ordinationer inkl. ordinationer, som lægen ændrer eller skriver recept på. For patientens øvrige ordinationer har lægen alene ansvar for interaktioner med egne ordinationer og for at sikre, at åbenlyse fejl rettes.

Kilde: Vejledning om brug af Fælles Medicinkort med fokus på dokumentation og kommunikation af medicinsk behandling og sektorovergange. Danske Regioner, 2015.

MEDICINGENNEMGANG I PRAKSIS

Ved medicingennemgang gennemgår lægen patientens aktuelle medicinliste og foretager relevante ændringer i samråd med patienten. Ændringerne kan fx være at seponere eller erstatte et præparat, som giver bivirkninger, ændre dosering eller ordinere et nyt præparat. Medicingennemgang inkluderer også, at man forholder sig til patientens compliance.

SÅDAN GØR DU

Medicingennemgang kan inddeles i tre trin:

TRIN 1
Forberedelse

- Medicinanamnese
- Overblik over diagnoser, paraklinik og sygehistorie

TRIN 2
Gennemgang

Kritisk vurdering af patientens lægemiddelbehandling
- Vurdér diagnoser
- Overvej hvilken form for behandling (fx forebyggende)
- Revurdér behandlingen
- Overvej klinisk betydende interaktioner
- Brug de 5 F'er (se s. 6)

TRIN 3
Opfølgning

Dialog med patienten om ændringer og gennemførelse af ændringer

Opdatér og ajourfør FMK

Følg op på ændringer

TRIN 1 – FORBEREDELSE

Medicinanamnese

Få overblik over hvilken medicin patienten tager, i hvilken dosering og vurdér compliance. Husk også at undersøge hvor ofte p.n. medicin anvendes. Inkludér gerne ikke-lægeordineret medicin. Plejepersonale eller pårørende kan være behjælpelige. Praksispersonalet kan med fordel foretage afstemningen med patienten og evt. sortere medicinen efter lægemiddelgruppe (ATC-gruppe).

TRIN 2 – GENNEMGANG

Vurdér diagnoser

Vurdér medicinen efter diagnoser. Er der medicin, som ikke har nogen indikation? Har patienten en behandlingskrævende diagnose, som ikke er behandlet? Er behandlingsmålet nået? Er der klinisk betydende interaktioner?

Overvej hvad der er livsvigtig, forebyggende eller symptomlindrende medicin

Vurdér om behandlingen er livsvigtig, symptomlindrende eller forebyggende. Ved forebyggende behandling hos skrøbelige, ældre er det vigtigt at tænke over forventet restlevetid - kan patienten nå at få gavn af behandlingen? (Se Seponeringslisten)

Brug de 5 F´er til at vurdere hvert enkelt lægemiddel

For hvad?
For meget?
For lidt?
Forkert?
For altid?

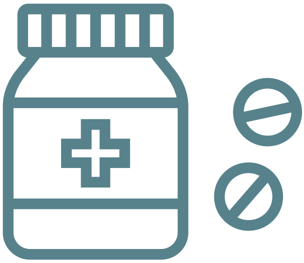

TRIN 3 – OPFØLGNING

- Prioriter ændringerne i samarbejde med patienten og udarbejd en behandlingsplan for ændringerne. Gennemfør ændringerne over flere gange
- Journaliser ændringerne
- Opdatér FMK (inkl. sletning af gamle recepter og oprettelse af nye)
- Ajourfør FMK
- Aftal opfølgning på ændringerne med patienten

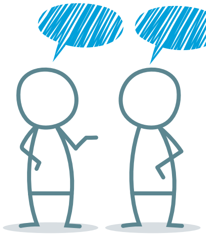

HVORDAN FINDER DU PATIENTERNE?

Der er flere tilgange til at finde patienter, der kan være kandidater til medicingennemgang. Nedenfor er en række forslag til, hvordan det kan organiseres i almen praksis.

Systematisk tilgang til medicingennemgang

Medicingennemgang kan gennemføres som en del af årskontrollen. Det sikrer en regelmæssig gennemgang af den enkelte patients brug af medicin. For patienter, som ikke kan komme i klinikken, kan medicingennemgang gennemføres i forbindelse med opsøgende/opfølgende hjemmebesøg. Koordinér evt. besøget med plejepersonalet.

Medicingennemgange kan også sættes i system ved kronologisk at gennemgå patienternes medicin hvert år efter fx fødselsdato.

Fokusområder i klinikken
Medicingennemgang kan også gennemføres hos en særlig patientgruppe, som klinikken beslutter at have fokus på. Fx patienter:

- med en bestemt diagnose
- i kommunal medicinadministration
- som er særligt sårbare
- der får dosisdispenseret medicin
- der er flyttet på plejehjem for nyligt
- i behandling med et bestemt lægemiddel eller lægemiddelgruppe
- i behandling med fx 6 eller flere receptpligtige lægemidler
- udskrevet fra sygehus fx inden for den sidste uge
- overtaget fra anden læge

Fokusområderne kan planlægges og organiseres i et årshjul.

Fremsøg patienter i lægesystemet
I de fleste lægesystemer er det muligt at trække en liste over patienter, som har en specifik diagnose, eller som er i behandling med bestemte lægemidler/lægemiddelgrupper. Det er ligeledes muligt i nogle lægesystemer at fremsøge klinikkens polyfarmacipatienter.

Vejledning til fremsøgning af polyfarmacipatienter i lægesystmet

Som tillæg til denne folder er der udarbejdet vejledninger til fremsøgning af polyfarmacipatienter i udvalgte lægesystemer. Vejledningerne findes på de regionale Lægemiddelenheders hjemmeside, se s. 11.

INDDRAGELSE AF PRAKSISPERSONALE

Praksispersonalet kan med fordel inddrages i arbejdet med medicingennemgange. Praksispersonalet kan finde relevante patienter efter kriterier besluttet i klinikken, afstemme medicinlisten i samarbejde med patienten og afklare patientens forbrug af p.n. medicin. Bed patienten medbringe sin medicin til konsultationen. Praksispersonalet kan også lave forarbejdet ved at gennemgå lægemidlerne for iøjefaldende uhensigtsmæssigheder bl.a. ved at anvende Seponeringslisten, Basislisten eller Interaktionsdatabasen.

MÅLSÆTNING OG EVALUERING

Sæt et realistisk mål første gang du beslutter at arbejde systematisk med medicingennemgang. Sæt tid af til en fælles evaluering af indsatsen i klinikken, hvor læger og praksispersonale deltager.

- Blev målet nået? Hvis ikke, hvorfor?
- Hvad skal ændres? Og hvem tager ansvaret herfor?
- Hvad skal målet være for den næste periode?
- Ny dato for evaluering.

HONORERING FOR MEDICINGENNEMGANGE

Medicingennemgang er ikke en ydelse med et særskilt honorar men kan være en naturlig del af fx årskontrol og opsøgende hjemmebesøg.

- Aftalt specifik forebyggelsesindsats (årskontrol) (ydelse 0120)
- Opsøgende hjemmebesøg for skrøbelige ældre (ydelse 0121)
- Opfølgende hjemmebesøg efter udskrivelse fra sygehus
  Den præcise aftale og ydelsesnummeret varierer fra region til region. Find din regions lokale aftale på www.plo.dk.

REDSKABER TIL MEDICINGENNEMGANG:

Der findes redskaber, som kan gøre det lettere at gennemføre medicingennemgang. Herunder er nævnt nogle af dem, og hvor de kan findes.

Seponeringslisten: www.sst.dk > Rationel Farmakoterapi > Medicingennemgang og -prioritering
Forslag til seponering af lægemidler hos voksne. Udarbejdet i samarbejde mellem regionerne og Sundhedsstyrelsen.

Basislisten: www.basislisten.dk
Regionale forslag til 1. valg af lægemidler til voksne inden for de mest almindelige indikationsområder i praksissektoren. Den kan vejlede ved start og revurdering af medicinsk behandling.

Interaktionsdatabasen: www.interaktionsdatabasen.dk
Her kan du indtaste patientens lægemidler, naturlægemidler og stærke vitaminer/mineraler. Du får et overblik over eventuelle interaktioner og alvorligheden af dem.

Liste over antikolinerge lægemidler:
www.sst.dk > Rationel Farmakoterapi > Medicingennemgang og -prioritering
Indeholder lægemidler med antikolinerge egenskaber, en graduering af den antikolinerge effekt for det enkelte lægemiddel og et forslag til et lignende lægemiddel med mindre antikolinerg effekt.

REGIONALE LÆGEMIDDELKONSULENTER

Regionerne har forskellige tilbud, der kan støtte almen praksis i arbejdet med medicingennemgang. Kontakt de regionale lægemiddelkonsulenter i din region og få mere at vide om de konkrete tilbud i din region.

Region Hovedstaden
Medicinfunktionen@regionh.dk
www.Medicinfunktionen.dk
www.kap-h.dk

Region Sjælland
Lmenheden@regionsjaelland.dk
www.regionsjaelland.dk/Sundhed/samarbejde-og-indsatser/medicin/Sider/laegemiddelenheden.aspx

Region Midtjylland
Lisero@rm.dk
www.sundhed.dk/sundhedsfaglig/information-til-praksis/midtjylland/almen-praksis/laegemidler/

Region Nordjylland
Nordkap@rn.dk
www.lme.rn.dk

Region Syddanmark
Wbh@rsyd.dk
www.sundhed.dk/sundhedsfaglig/information-til-praksis/syddanmark/almen-praksis/konsulenthjaelp-til-praksis/laegemiddelteam/

SUNDHEDSSTYRELSEN
Rationel farmakoterapi

midt
regionmidtjylland

REGION NORDJYLLAND

REGION
Region Hovedstaden

Region Syddanmark

REGION SJÆLLAND
– vi er til for dig

02305 - 10.18
