# Supplementary material for: Deprescribing: What is the gold standard? Themes that characterized the discussions at the first Danish symposium on evidence-based deprescribing
Source: Explor Res Clin Soc Pharm. 2022 Jan 7;5:100102. doi: 10.1016/j.rcsop.2022.100102 (PMC9030658; doi:10.1016/j.rcsop.2022.100102)
Supplement: Supplementary file 6 — Supplementary material 6 Appendix F Deprescribing list 2021. Danish Health Authority. [file mmc6.pdf]

SUNDHEDSSTYRELSEN
Rationel farmakoterapi

Seponeringslisten 2021

Forslag til seponering af hyppigt anvendte lægemidler hos voksne

Tag stilling til alle lægemidler ved medicingennemgang

Du kan forsøge seponering ved de fleste lægemidler. Ophør med velindiceret medicin kan være nødvendigt, hvis lægemidlet fx giver uacceptable bivirkninger, er uden effekt, eller patienten ikke ønsker medicinen. Medicingennemgang er særligt relevant hos ældre, som er mere følsomme for bivirkninger og oftere udsat for polyfarmaci. Brug nedenstående tommelfingerregler.

Folderen "Medicingennemgang i praksis" giver dig som praktiserende læge en metode til at planlægge, gennemføre og følge op på en medicingennemgang.

Læs mere på
sst.dk/da/viden/laegemidler/medicingennemgang

Tommelfingerregler

- Vurdér behandlingsvarighed ved ordination
- Tag udgangspunkt i det, der er vigtigt for patienten og det samlede sygdomsbillede (fx interaktioner og paraklinik)
- Vurdér om behandlingen er livsvigtig, symptomlindrende eller forebyggende
- Seponér medicin, hvor der ikke længere er indikation for behandlingen – ville behandlingen være relevant at starte i dag?
- Seponér forebyggende behandling, hvis den forventede effekt ikke står i rimeligt forhold til patientens forventede restlevetid[1]
- Seponér medicin, som ikke giver klinisk relevant effekt, eller hvor forholdet mellem effekt og bivirkninger er ugunstigt

Seponering er en proces

- Lav en plan og en klar aftale med patienten. Inddrag evt. pårørende og plejepersonale. En vellykket seponering er en fælles proces
- Lav kun om på én eller få ting ad gangen
- Start evt. med reduktion af dosis. Specielt psykofarmaka, andre CNS-aktive stoffer og opioider bør aftrappes. Aftrapning kan mindske seponeringssymptomer og rebound-effekt. Overvej om det er seponeringssymptomer eller opblussen i sygdom
- Følg altid op på en seponering. Hvis du må genoptage en seponeret behandling, er det ikke udtryk for fejl, men grundighed

Kildehenvisninger, høringsparter og kontaktoplysninger:
Se onlineudgave på **sst.dk/seponeringslisten**

Sundhed for alle

Symptomlindrende behandling – overvej effekt og alternativer

Lægemiddel	Hvornår kan du overveje seponering?	Hvordan kan du seponere?
**PROTONPUMPEHÆMMERE (PPI)**		
Esomeprazol<br>Lansoprazol<br>Omeprazol<br>Pantoprazol<br>Rabeprazol	**Seponér:**<br>– ved **funktionel dyspepsi** (symptomer uden organisk forklaring)<br>– ved **ukompliceret ulcus** (ingen blødning eller perforation) når patienten er symptomfri (tager op til 4 uger)<br>– ved **ophør af ulcerogene lægemidler** fx NSAID (inkl. ASA), hvor PPI alene er givet som ulcusprofylakse<br><br>PPI er bl.a. associeret med øget risiko for pneumoni ved korttidsbehandling samt tarminfektioner og frakturer ved langtidsbehandling	Seponér brat eller ved aftrapning, hvor dosis halveres hver 4. uge<br><br>Efter behandling i mere end 8 uger kan der forekomme øget syresekretion (rebound). Brug i disse tilfælde syre-neutraliserende midler p.n. i en periode
**MIDLER MOD KVALME**		
Metoclopramid AC<br>Domperidon	**Seponér:**<br>– inden for 5 dage for metoclopramid<br>– inden for 7 dage for domperidon<br><br>Der er set alvorlige bivirkninger fx ekstrapyramidale og kardielle bivirkninger	Seponér brat
**LOOP-DIURETIKA**		
Bumetanid<br>Furosemid	**Seponér:**<br>– ved ukompliceret hypertension<br>– ved perifere ødemer uden kardiel årsag<br><br>**Nedtrap til lavest mulig dosis:**<br>– ved hjerteinsufficiens. Effekten er kun symptomatisk<br><br>Diuretika er hyppig årsag til indlæggelser pga. fald, elektrolytforstyrrelser mv.	Seponér ved nedtrapning<br><br>Husk vægt- og elektrolytkontrol og hold øje med evt. recidiv af inkompensation
**MIDLER VED OVERAKTIV BLÆRESYNDROM**		
Fx:<br>**Solifenacin** AC<br>**Tolterodin** AC<br>**Fesoterodin** (Toviaz) AC<br>**Trospiumchlorid** AC<br>**Mirabegron** (Betmiga)	**Seponér:**<br>– efter 1-2 måneders behandling ved manglende effekt<br>– ved permanent kateter<br><br>**Overvej seponering:**<br>– ved langvarig behandling<br><br>Midlerne har beskeden effekt, mange bivirkninger og interaktioner	Seponér brat
**NSAID**		
Fx:<br>**Ibuprofen**<br>**Naproxen**<br>**Diclofenac**<br>**Celecoxib**	**Seponér:**<br>– ved kroniske smerter uden inflammatorisk komponent<br>– ved svært nedsat nyre- eller leverfunktion<br>– ved svær hjerteinsufficiens og/eller svær iskæmisk hjertesygdom<br>– ved blødningstendens fx ved AK-behandling<br><br>**Overvej seponering:**<br>– ved hjertekarsygdom eller høj risiko for det<br>– ved høj risiko for ulcuskomplikation<br>– ved samtidig behandling med andre lægemidler, som øger blødnings-risikoen (fx trombocythæmmere, systemisk glukokortikoid eller SSRI)<br>– ved samtidig behandling med NSAID + diuretika + ACE-hæmmere/ AT-II-antagonister pga. risiko for nyresvigt (triple whammy)	Seponér brat
**MUSKELAFSLAPPENDE MIDLER**		
**Chlorzoxazon**<br>(fx Klorzoxazon "DAK") △<br>**Baklofen** △ AC<br>**Tizanidin** △ AC	**Seponér:**<br>– ved akut, uspecifikt lændehold<br><br>**Overvej seponering:**<br>– ved øvrige tilstande<br><br>Midlerne har ringe effekt og mange CNS-relaterede bivirkninger som fx svimmelhed og træthed	Chlorzoxazon: seponér brat<br>Baklofen: aftrap over 1-2 uger<br>Tizanidin: aftrap efter længere tids brug

Symptomlindrende behandling – overvej effekt og alternativer

Lægemiddel	Hvornår kan du overveje seponering?	Hvordan kan du seponere?
**OPIOIDER**		
Fx:<br>**Tramadol** △<br>**Kodein** △<br>**Morfin** △<br>**Oxycodon** (fx OxyContin) △<br>**Fentanyl** △<br>**Buprenorphin** △	**Seponér:**<br>– **fast dosering af hurtigtvirkende opioider**<br>Anvend i stedet depotmorfin, der giver bedre døgndækning, færre gennembrudssmerter og mindre euforiserende effekt<br>– **smerteplastre hos patienter, som kan tage tabletter**<br>Plastre er forbundet med større variation i biotilgængelighed og flere utilsigtede hændelser. Overvej fortsat indikation for opioid særligt ved lavdosisplastre. Ved behov for opioid, omlæg til depotmorfin<br>– **kodein**<br>Den smertestillende effekt skyldes, at kodein omdannes til morfin. Ca. 10% af befolkningen kan ikke omdanne kodein til morfin.<br>25 mg kodein ≈ 2,5 mg morfin<br><br>**Overvej seponering:**<br>– **ved kroniske, non-maligne smerter**<br>Pga. sparsom evidens for effekt og bivirkninger. Ved behov for opioid begræns dosis til max 100 mg morfinækvivalenter per døgn<br>– **af tramadol.**<br>Tramadol kan give serotonerge bivirkninger, som forstærkes ved samtidig brug af serotonerge lægemidler (fx antidepressiva og triptaner). Ca. 10% af befolkningen kan ikke omdanne tramadol til den aktive metabolit.<br>50 mg tramadol ≈ 10 mg morfin	Seponering af opioider bør ske efter individuel plan<br><br>Efter kortvarig behandling (< 6 uger): reducér døgndosis med 10-20% hver 3-5 dag<br><br>Efter langvarig behandling: reducér døgndosis med 5-20% med 1-2 ugers mellemrum<br><br>Giv ikke oral opioid inden for ca. 18 timer efter fjernelse af fentanyl-plastre og ca. 24 timer efter buprenorphin-plastre<br><br>Klip ikke depotplastre over
**PARACETAMOL VED KRONISKE NON-MALIGNE SMERTER**		
Paracetamol	**Overvej seponering:**<br>Effekt ved langtidsbehandling (> 6 uger) er ikke undersøgt, men nogle kan have gavn af behandlingen	Seponér brat
**BENZODIAZEPINER**		
Fx:<br>**Diazepam** △<br>**Oxazepam** △<br>**Alprazolam** △<br>**Nitrazepam** △<br>**Triazolam** (Halcion) △<br>**Chlordiazepoxid** (fx Risolid) △	**Seponér:**<br>– **ved angst og uro inden for 4 uger**<br>Behandling bør ikke overstige 4 uger pga. risiko for afhængighed. Effekten aftager efter 3-4 mdr., mens nogle af bivirkningerne kan bestå<br>– **ved søvnbesvær inden for 2 uger**<br>Den søvnfremkaldende virkning aftager hurtigt, mens bivirkningerne består (fx dagsedation, svimmelhed med øget faldtendens og kognitiv svækkelse)	Efter kortvarig behandling (almindeligvis 1-2 uger og højst 4 uger): seponér over få dage<br><br>Efter langvarig behandling: reducér dosis med ca. 10-20% hver anden uge. Ved seponeringssymptomer: forlæng tiden mellem dosisreduktionerne, eller foretag langsommere dosisreduktion. Kan især være nødvendigt sidst i forløbet
**BENZODIAZEPINLIGNENDE STOFFER VED SØVNBESVÆR**		
Fx:<br>**Zopiclon** △<br>**Zolpidem** △	**Seponér:**<br>– inden for 2 uger<br><br>Den søvnfremkaldende virkning aftager hurtigt, mens bivirkningerne består (fx dagsedation, svimmelhed med øget faldtendens og kognitiv svækkelse)	Efter kortvarig behandling (almindeligvis 1-2 uger og højst 4 uger): seponér over få dage<br><br>Efter langvarig behandling: reducér dosis med ca. 10-20% hver anden uge. Ved seponeringssymptomer: forlæng tiden mellem dosisreduktionerne, eller foretag langsommere dosisreduktion. Kan især være nødvendigt sidst i forløbet
**SEDERENDE ANTIHISTAMINER**		
**Promethazin** △ AC<br>**Cyclizin** (fx Marzine) △ AC	**Seponér:**<br>– **ved søvnløshed**<br>Effekten er lille til moderat, og der udvikles hurtigt tolerance. Risiko for dagsedation. Der er rapporteret tilfælde af misbrug<br><br>Ved brug af sederende antihistaminer bør der sædvanligvis udstedes kørselsforbud i 24 timer efter indtagelsen	Seponér brat

Symptomlindrende behandling – overvej effekt og alternativer

Lægemiddel	Hvornår kan du overveje seponering?	Hvordan kan du seponere?
**MIDLER MOD DEPRESSION**		
Fx:<br>**Sertralin**<br>**Citalopram**<br>**Duloxetin**<br>**Venlafaxin**<br>**Mirtazapin** AC<br>**Nortriptylin** (Noritren) AC	**Overvej seponering:**<br>– **ved depression efter ½-1 års symptomfrihed hos patienter, som kun har haft én depressiv episode**<br>Hvis patienten har haft en eller flere depressioner tidligere, eller hvis andre risikofaktorer for tilbagefald er til stede, anbefales det at fortsætte behandlingen i minimum 2 år efter opnået remission.<br>Fortsat behov for behandling revurderes årligt<br>– **ved demens uden kendt affektiv sygdom efter ½-års behandling**	Seponér ved langsom aftrapning over én til flere måneder efter en individuel plan. Reducer dosis med fx 25-50% med 1-2 ugers mellemrum
**ANTIPSYKOTIKA TIL PERSONER MED DEMENS UDEN SAMTIDIG PSYKISK LIDELSE**		
1.generation fx:<br>**Haloperidol** (fx Serenase) AC<br><br>2. generation fx:<br>**Aripiprazol** AC<br>**Olanzapin** AC<br>**Quetiapin** AC<br>**Risperidon** AC	**Seponér:**<br>– **al behandling med 1. generations antipsykotika**<br>Risikoen for ekstrapyramidale bivirkninger og QT-forlængelse er lavere for 2. generations antipsykotika<br>– **langvarig behandling med 2. generations antipsykotika (>3 mdr.)**<br>Behandling med antipsykotika kan føre til øget dødelighed hos personer med demens<br><br>Revurder jævnligt fortsat indikation for behandlingen, også selvom behandlingen er startet på hospital eller hos speciallæge i psykiatri. I tilfælde, hvor langvarig behandling er indiceret, sker behandlingen i samarbejde med speciallæge i psykiatri	Seponér ved aftrapning<br><br>Lavdosisbehandling kan seponeres brat
**MIDLER MOD DEMENS**		
**Donepezil**<br>**Galantamin**<br>**Memantin**<br>**Rivastigmin**	**Seponér:**<br>– **ved meget svær demens** (fx sengeliggende uden sprog)<br><br>**Overvej seponering:**<br>Overvej løbende om der er effekt af behandlingen. Genoptag behandlingen i samråd med pårørende/plejepersonale ved markant forværring inden for 2-4 uger efter seponering.<br><br>OBS! Behandlingen optitreres til slutdosis iht. produktresume	Seponér brat
**KININ**		
Kinin	**Seponér:**<br>– **ved hjertesvigt**<br>Kinin er associeret med øget risiko for død hos patienter med hjertesvigt<br>– **ved Restless Legs Syndrome**<br>Effekten er usikker, og der er risiko for konfusion samt interaktioner<br><br>**Overvej seponering:**<br>– **ved natlige lægkramper**<br>Der er modstridende og ringe evidens for reduktion af krampernes antal og varighed og moderat evidens for reduktion af krampernes intensitet	Seponér brat

Symboler

Seponeringslisten er ikke udtømmende for lægemidler med disse egenskaber. Der kan være stor forskel mellem lægemidlerne i graden af påvirkning inden for symboltyper. Brug symbolerne til at give indblik i patientens samlede belastning med henholdsvis trafikfarlige og antikolinerge lægemidler.

△ **Trafikfarlig**
Kilde: Produktresuméer

AC **Antikolinerg virkning**
Centrale: svimmelhed, faldtendens, hukommelsesbesvær, konfusion.
Perifere: mundtørhed, urinretention, kvalme, obstipation, uskarpt syn.
Kilde: Månedsbladet Rationel Farmakoterapi 11, 2017 Antikolinerge lægemidler; IRFs liste over antikolinerge lægemidler

Forebyggende behandling – overvej bivirkninger og restlevetid[1]

Lægemiddel	Hvornår kan du overveje seponering?	Hvordan kan du seponere?
**TROMBOCYTHÆMMERE**		
Acetylsalicylsyre (ASA)	**Seponér:**<br>– ved ingen manifest kardiovaskulær sygdom<br>– ved diabetes og *lav* risiko for kardiovaskulær sygdom<br><br>ASA anbefales ikke som primær profylakse, da effekten ikke opvejer blødningsrisikoen<br><br>**Overvej seponering:**<br>– ved diabetes og *høj* risiko for kardiovaskulær sygdom<br><br>Enterocoating af ASA (fx Hjertealbyl) mindsker ikke risikoen for GI-bivirkninger	Seponér brat
**Trombocythæmmer i kombination med enten antikoagulerende behandling eller anden trombocythæmmer**<br><br>Fx:<br>**ASA og warfarin**<br>**ASA og DOAK (NOAK)** (fx Xarelto)<br>**ASA og ADP-hæmmer** (fx Plavix)	**Overvej seponering:**<br>– af enten trombocythæmmer eller antikoagulerende behandling, hvis der ikke er lagt en plan for det<br><br>Livslang kombinationsbehandling er meget sjældent indiceret. Efter akut kardio-vaskulær sygdom (fx akut koronart syndrom eller visse typer apopleksi) gives det hos udvalgte patienter i en fast defineret periode, ofte 3-12 måneder. Derefter skal der ofte fortsættes med kun én type trombocythæmmer *eller* antikoagulerende behandling. Ved tvivlsspørgsmål bør man konferere med relevant specialist	Seponér brat
**STATINER**		
**Atorvastatin**<br>**Fluvastatin**<br>**Lovastatin**<br>**Pravastatin**<br>**Rosuvastatin**<br>**Simvastatin**	**Seponér:**<br>– **ved lav risiko for at dø af kardiovaskulær sygdom inden for 10 år**[2], med mindre der er markant forhøjelse af én enkelt risikofaktor, kardiovaskulær sygdom, diabetes, kronisk nyresygdom eller familiær hyperkolesterolæmi<br>– **ved kort forventet restlevetid**[1]	Seponér brat
**FOREBYGGELSE MED ANTIBIOTIKA VED GENTAGNE URINVEJSINFEKTIONER**		
**Pivmecillinam** (fx Selexid)<br>**Sulfamethizol**<br>**Trimethoprim** (Trimopan)<br>**Nitrofurantoin**	**Seponér:**<br>Antibiotisk behandling til forebyggelse af gentagne UVI frarådes generelt pga. manglende evidens for effekt og risiko for resistensudvikling<br><br>Ved langtidsbehandling med nitrofurantoin kan der udvikles lungefibrose	Seponér brat
**ALENDRONAT VED KNOGLESKØRHED**		
**Alendronat**	**Overvej seponering:**<br>– **efter minimum 5 år, hvis** patienten aldrig har haft lavenergifraktur i columna eller hofte, og T-score (i hoften) efter behandlingen er > -2,5, og at der ikke har været øvrige lavenergifrakturer (ekskl. columna og hofte) i perioden. Derudover skal evt. udløsende årsager være korrigeret (fx længerevarende prednisolonbehandling)<br><br>Hos patienter, der før eller efter opstart af behandling har haft en lavenergi-fraktur i columna eller hofte, kan det være nødvendigt at fortsætte behandling i mere end 10 år, muligvis livslangt<br><br>Der foreslås kontrol af BMD (knoglevævets mineraltæthed) 1-2 år efter behandlingsophør. Ved faldende BMD, som er større end måleusikkerheden på 5 %, kan det overvejes at genoptage behandlingen	Seponér brat
**BISFOSFONATER VED SYSTEMISK GLUKOKORTIKOIDBEHANDLING**		
**Alendronat**<br>**Risedronat** (fx Optinate Septimum)	**Seponér:**<br>– **6 - 12 måneder efter ophør af systemisk glukokortikoidbehandling, hvis** T-score er > -2,5, og patienten ikke har haft osteoporotiske brud	Seponér brat
**INHALATIONSSTEROID (ICS) VED KOL UDEN KOMPONENT AF ASTMA**		
**ICS** (fx Pulmicort, Flixotide)<br>**ICS + LABA** (fx Bufomix, Symbicort, Seretide)<br>**ICS + LABA + LAMA** AC<br>(fx Trimbow)	**Overvej seponering:**<br>– ved stabil KOL (uden eksacerbationer eller indlæggelser på grund af KOL gennem mindst et år)<br>– ved behandling med ICS+LABA gennem længere tid, og hvor indikationen i udgangspunktet ikke har været helt klar<br><br>Hvis patienten har en komponent af astma, er ICS dog indiceret	Seponer ved nedtrapning. Halvér ICS og afvent effekt i 3 mdr.<br><br>Hvis FEV>50% (indledningsvist), og ingen forværring kan ICS seponeres helt efter yderligere 3 mdr.<br><br>Hvis FEV1<50% (indledningsvist), eller ved forværring bør yderligere nedtrapning ikke foretages<br><br>Man kan dog efter klinisk skøn – uafhængig af FEV1 – vælge at aftrappe patienterne helt af ICS

Noter

[1] Den statistiske, gennemsnitlige restlevetid for henholdsvis en 75-, 85- og 95-årig: Mand: 11, 6 og 2 år; Kvinde: 13, 7 og 3 år. Tal fra Danmarks Statistik 2018/2019 (statistikbanken.dk/hisb8).

[2] Brug fx den europæiske SCORE-tabel for lavrisikolande fra: Dansk Cardiologisk Selskab, NBV, kap. 34: Forebyggelse af hjertesygdom.

Listen er udarbejdet af IRF i Sundhedsstyrelsen i samarbejde med en arbejdsgruppe med repræsentanter fra Region Nordjylland, Region Midtjylland, Region Syddanmark, Region Sjælland, Region Hovedstaden, Dansk Selskab for Geriatri, Dansk Selskab for Klinisk Farmakologi og Dansk Selskab for Almen Medicin

Udgivet november 2020

REGION NORDJYLLAND – i gode hænder

midt regionmidtjylland

Region Syddanmark

REGION SJÆLLAND – vi er til for dig

REGION Region Hovedstaden

DSG Dansk Selskab for Geriatri

DSKF
