# Supplementary material for: Deprescribing: What is the gold standard? Themes that characterized the discussions at the first Danish symposium on evidence-based deprescribing
Source: Explor Res Clin Soc Pharm. 2022 Jan 7;5:100102. doi: 10.1016/j.rcsop.2022.100102 (PMC9030658; doi:10.1016/j.rcsop.2022.100102)
Supplement: Supplementary file 7 — Supplementary material 7 Appendix G National clinical guideline regarding deprescribing and continued treatment with inhaled steroid in patients with COPD. [file mmc7.pdf]

National klinisk retningslinje omhandlende seponering af og fortsat behandling med inhalationssteroid til patienter med kronisk obstruktiv lungesygdom

REGION H
Bispebjerg og Frederiksberg Hospital

DANSK LUNGEMEDICINSK SELSKAB
DANISH SOCIETY OF RESPIRATORY MEDICINE

Kontaktperson

Dansk Lungemedicinsk Selskab

dls.dlsoffice@gmail.com
42 67 05 50
www.lungemedicin.dk

Sponsorer / Finansiering

Denne nationale kliniske retningslinje er udarbejdet med midler bevilliget under Finansloven 2017 til udarbejdelse af nationale kliniske retningslinjer i perioden 2017-2020.

Ansvarsfraskrivelse

Denne nationale kliniske retningslinje skal betragtes som vejledende og fritager ikke sundhedspersoner for individuelt ansvar for at træffe korrekte beslutninger vedrørende den individuelle patient, i samarbejde med og under hensyntagen til denne. De nationale kliniske retningslinjer er ikke juridisk bindende, og det vil altid være det faglige skøn i den konkrete kliniske situation, der er afgørende for beslutningen om passende og korrekt sundhedsfaglig ydelse. Der er ingen garanti for et succesfuldt behandlingsresultat, selvom sundhedspersoner følger anbefalingerne. I visse tilfælde vil anden behandling end den anbefalede være at foretrække, fordi den passer bedre til patientens situation.

Version
Version 1.0 udgivet d. 15. marts 2021

Indhold

Centrale budskaber ... 4

1 - Læsevejledning ... 7

2 - Indledning ... 9

3 - Patienter med KOL i behandling med ICS i forhold til EOS niveauer ... 10

3.1 - Patienter med KOL i behandling med ICS og med EOS under 150 celler/ µL ... 10

3.2 - Patienter med KOL i behandling med ICS og med EOS mellem 150 og 300 celler/µL ... 20

3.3 - Patienter med KOL i behandling med ICS og med EOS over 300 celler/µL ... 27

4 - Patienter med KOL i behandling med ICS og uden moderat til svær exacerbation det seneste år ... 34

5 - Patienter med KOL i behandling med ICS og med hyppige, tilbagevendende pneumonier ... 41

Bilag 1: Implementering ... 46

Bilag 2: Monitorering ... 47

Bilag 3: Opdatering og videre forskning ... 48

Bilag 4: Beskrivelse af anvendt metode ... 49

Bilag 5: PICO ... 50

Bilag 6: Beskrivelse af anbefalingernes styrke og implikationer ... 56

Bilag 7: Evidensvurderinger ... 58

Bilag 8: Arbejdsgruppen og referencegruppen ... 59

Bilag 9: Begreber og forkortelser ... 60

Bilag 10: Søgebeskrivelse ... 64

Referencer ... 66

Centrale budskaber

3.1 - Patienter med KOL i behandling med ICS og med EOS under 150 celler/ µL

Svag anbefaling mod

Fortsæt kun behandling med inhalationssteroid (ICS) til patienter med kroniske obstruktiv lungesygdom (KOL) og blod-eosinofilocytter (EOS) under 150 celler/µL efter nøje overvejelser.

Patienter med KOL og EOS under 150 celler/µL forventes kun at have sparsom effekt af fortsat behandling med ICS.

Studierne viser en reduktion af risiko for moderat til svær exacerbation på 12 % (konfidensinterval 6-17 %). Hvis patienten fx har en moderat til svær exacerbation om året, vil dette svare til én exacerbation mindre i løbet af 10 år. Patienten vil stadig rammes af de 9 andre exacerbationer.

Arbejdsgruppen anbefaler på baggrund af klinisk erfaring at seponere ICS gradvist i samarbejde med patienten. Fx reducér til halv dosis og fortsæt i 6-8 uger efterfulgt af seponering. Kan patienten ikke undvære ICS, anbefaler arbejdsgruppen, at behandlingen genoptages i lavest mulige effektive dosis. Se i øvrigt DLS' KOL vejledning.

3.2 - Patienter med KOL i behandling med ICS og med EOS mellem 150 og 300 celler/µL

Svag anbefaling

Overvej at fortsætte behandling med inhalationssteroid (ICS) til patienter med kronisk obstruktiv lungesygdom (KOL) og blod-eosinofilocytter (EOS) mellem 150-300 celler/µL.

Patienter med KOL og EOS mellem 150-300 celler/µL forventes at have nogen effekt af fortsat behandling med ICS. Dette er særlig udtalt hos patienter med hyppige moderate til svære exacerbationer og/eller lav lungefunktion, da der er vist effekt på disse outcomes.

Studierne viser en reduktion af risiko for moderat til svær exacerbation på 20 % (konfidensinterval 6-31 %). Hvis patienten fx har én moderat til svær exacerbation om året vil dette svare til to exacerbationer mindre i løbet af 10 år. Patienten vil stadig rammes af de 8 andre exacerbationer.

Studierne viser en forbedring i lungefunktion (FEV1) på 50 mL (konfidensinterval 30-70 mL). Denne forbedring vil umiddelbart betyde mest for patienter med svært nedsat lungefunktion.

Hvis det ønskes at forsøge seponering, anbefaler arbejdsgruppen på baggrund af klinisk erfaring at seponere ICS gradvist i samarbejde med patienten. Fx reducér til halv dosis og fortsæt i 6-8 uger efterfulgt af seponering. Kan patienten ikke undvære ICS, anbefaler arbejdsgruppen, at behandlingen genoptages i lavest mulige effektive dosis. Se i øvrigt DLS' KOL vejledning.

3.3 - Patienter med KOL i behandling med ICS og med EOS over 300 celler/µL

Svag anbefaling

Overvej at fortsætte behandling med inhalationssteroid (ICS) til patienter med kronisk obstruktiv lungesygdom (KOL) og blod-eosinofilocytter (EOS) over 300 celler/µL.

Patienter med KOL og EOS over 300 celler/µL forventes at have effekt af fortsat behandling med ICS. Effekt er vist på forbedring af livskvalitet, lungefunktion og nedsat risiko for moderat til svær exacerbation.

Studierne viser en reduktion af risiko for moderat til svær exacerbation på 43 % (konfidensinterval 34-51 %). Hvis patienten fx har en moderat til svær exacerbation om året vil dette svare til 4 exacerbation mindre i løbet af 10 år. Patienten vil stadig rammes af de 6 andre exacerbationer.

Studierne viser en forbedring i lungefunktion (FEV1) på 60 mL (konfidensinterval 40-90 mL). Denne forbedring vil forventeligt betyde mere for patienter med svært nedsat lungefunktion, og mindre for patienter med let nedsat lungefunktion.

Studierne viser en klinisk relevant forbedring i livskvalitet på ca. 4 point (konfidensinterval 1-7 point) på SGRQ-skalaen fra 0-100.

Arbejdsgruppen anbefaler på baggrund af klinisk erfaring at reducere dosis af ICS til mindst mulige effektive dosis. Fx reducér og fortsæt med halv dosis. Se i øvrigt DLS' KOL vejledning.

4 - Patienter med KOL i behandling med ICS og uden moderat til svær exacerbation det seneste år

Svag anbefaling

Overvej at fortsætte behandling med inhalations steorid (ICS) til patienter med kronisk obstruktiv lungesygdom (KOL) der har været uden moderat til svær exacerbation det sidste år.

Patienter med KOL uden moderat til svær exacerbation det seneste år forventes af have effekt af fortsat behandling med ICS. Effekten er vist ved en mulig nedsat risiko for moderat til svær exacerbation og mindre frafald i studierne.

Arbejdsgruppen anbefaler på baggrund af klinisk erfaring at reducere dosis af ICS til mindst mulige effektive dosis. Fx reducér og fortsæt med halv dosis. Se i øvrigt DLS' KOL vejledning.

5 - Patienter med KOL i behandling med ICS og med hyppige, tilbagevendende pneumonier

Svag anbefaling mod

Fortsæt kun behandlingen med inhalationssteroid (ICS) til patienter med kronisk obstruktiv lungesygdom (KOL) og hyppige, tilbagevendende pneumonier, efter nøje overvejelser.

Patienter med KOL og hyppige, tilbagevendende pneumonier forventes kun at have sparsom effekt af fortsat behandling med ICS. Det er arbejdsgruppens vurdering, at risikoen for pneumoni må opvejes mod risiko for exacerbationer hos den enkelte.

Ingen studier omfattede risiko for moderat til svær exacerbation, frafald eller bivirkninger for denne gruppe patienter.

Et enkelt studie viser en øget risiko for pneumoni på 146 % (konfidensinterval 47-312 %).

Arbejdsgruppen anbefaler på baggrund af klinisk erfaring at seponere ICS gradvist i samarbejde med patienten. Fx reducér til halv dosis og fortsæt i 6-8 uger efterfulgt af seponering. Kan patienten ikke undvære ICS, anbefaler arbejdsgruppen, at behandlingen genoptages i lavest mulige effektive dosis. Se i øvrigt DLS' KOL vejledning.

1 - Læsevejledning

Retningslinjen er bygget op i to lag:

1. Lag - Anbefalingen

Stærk anbefaling for (Grøn)
Der gives en stærk anbefaling for, når der er evidens af høj kvalitet, der viser, at de samlede fordele ved interventionen er klart større end ulemperne. Det betyder, at alle, eller næsten alle, patienter vil ønske den anbefalede intervention

Stærk anbefaling imod (Rød)
Der gives en stærk anbefaling imod, når der er evidens af høj kvalitet, der viser, at de samlede ulemper ved interventionen er klart større end fordelene. Der anvendes også en stærk anbefaling imod, når gennemgangen af evidensen viser, at en intervention med stor sikkerhed er nytteløs.

Svag/betinget anbefaling for (Gul)
Der gives en svag/betinget anbefaling for interventionen, når det vurderes, at fordelene ved interventionen er større end ulemperne, eller den tilgængelige evidens ikke kan udelukke en væsentlig fordel ved interventionen, samtidig med at det vurderes, at skadevirkningerne er få eller fraværende. Denne anbefaling anvendes også, når det vurderes, at patienters præferencer varierer.

Svag/betinget anbefaling imod (Orange)
Der gives en svag/betinget anbefaling imod interventionen, når det vurderes, at ulemperne ved interventionen er større end fordelene, men hvor dette ikke er underbygget af stærk evidens. Denne anbefaling anvendes også, hvor der er stærk evidens for både gavnlige og skadelige virkninger, men hvor balancen mellem dem er vanskelige at afgøre. Ligeledes anvendes den også, når det vurderes, at patientens præferencer varierer.

God praksis (Grå)
God praksis anvendes, når der ikke foreligger relevant evidens, og bygger således på faglig konsensus blandt medlemmerne af arbejdsgruppen, der har udarbejdet den kliniske retningslinje. Anbefalingen kan være enten for eller imod interventionen. Da der er tale om faglig konsensus, er denne type anbefaling svagere end de evidensbaserede anbefalinger, uanset om de evidensbaseret er stærke eller svage.

Se bilag "Beskrivelse af anbefalingernes styrke og implikationer" for mere information

2. Lag – Grundlaget for anbefalingen

Klik på anbefalingen, hvis du vil vide mere om grundlaget for anbefalingen

Evidensprofilen: De samlede effektestimater samt referencer til studierne.

Sammenfatning: Overblik over samt kort gennemgang af den tilgrundliggende evidens

Kvaliteten af evidensen:

- Høj: Vi er meget sikre på, at den sande effekt ligger tæt på den estimerede effekt
- Moderat: Vi er moderat sikre på den estimerede effekt. Den sande effekt ligger sandsynligvis tæt på denne, men der er en mulighed for, at den er væsentligt anderledes
- Lav: Vi har begrænset tiltro til den estimerede effekt. Den sande effekt kan være væsentligt anderledes end den estimerede effekt
- Meget lav: Vi har meget ringe tiltro til den estimerede effekt. Den sande effekt vil sandsynligvis være væsentligt anderledes end den estimerede effekt.

Nøgleinformation: Kort beskrivelse af gavnlige og skadelige virkninger, kvaliteten af evidensen og overvejelser om patientpræferencer.

Rationale: Beskrivelse af hvorledes de ovenstående elementer blev vægtet i forhold til hinanden og resulterede i den aktuelle anbefalings retning og styrke.

Praktiske oplysninger: Praktisk information vedrørende behandlingen og oplysninger om eventuelle særlige patientovervejelser.

Adaption: Såfremt anbefalingen er adapteret fra en anden retningslinje, findes her en beskrivelse af eventuelle ændringer.

Diskussion: Hvis du er logget ind som bruger, kan du her komme med kommentarer til specifikke anbefalinger.

Referencer: Referenceliste for anbefalingen.

Den anvendte graduering af evidensens kvalitet og anbefalingsstyrke baseres på GRADE (Grading of Recommendations Assessment, Development and Evaluation).

For en hurtig og informativ introduktion til GRADE anbefales følgende artikel G.Goldet, J.Howick. Understanding GRADE: an introduction. Journal of Evidence-Based Medicine 6 (2013) 50-54. Se også: http://www.gradeworkinggroup.org.

Desuden henvises der til Sundhedsstyrelsens metodehåndbog for en overordnet introduktion til metoden bag udarbejdelsen af de Nationale Kliniske Retningslinjer.

2 - Indledning

Formål

Formålet med de nationale kliniske retningslinjer er at understøtte en evidensbaseret indsats af ensartet høj kvalitet på tværs af landet, medvirke til hensigtsmæssige patientforløb og vidensdeling på tværs af sektorer og faggrupper samt prioritering i sundhedsvæsenet. En national klinisk retningslinje indeholder alene konkrete handlingsanvisninger indenfor udvalgte, velafgrænsede kliniske problemstillinger (dvs. hvad der skal gøres og hvem er det relevant for). Den har ikke som primært formål at afklare visitation og organisering af indsatsen (hvem der skal tilbyde indsatsen) eller samfundsøkonomiske konsekvenser (hvad er den afledte effekt på ressourcerne og er disse til stede). Disse typer af problemstillinger kan eksempelvis håndteres i en visitationsretningslinje, et pakkeforløb, et forløbsprogram, et referenceprogram eller en medicinsk teknologivurdering (MTV).

Anbefalingerne til den farmakologisk grundbehandling af patienter med kronisk obstruktiv lungesygdom (KOL) fra Global Initiative for Chronic Obstructive Lung Disease (GOLD) er langtidsvirkende antikolinergikum (LAMA) og/eller langtidsvirkende beta-2-agonister (LABA) *[1]*. Derudover kan tillæg af inhalationssteroid (ICS) være relevant. Behandling med ICS bør overvejende startes ved astmatisk komponent og/eller som respons på gentagende exacerbationer og/eller vedvarende symptomer. Mange patienter med svær KOL er i behandling med alle tre stoffer (LABA, LAMA og ICS) *[1]*. Studier har vist, at behandling med ICS kan være forbundet med bivirkninger *[2]*. Og European Medicines Agency (EMA) fik i 2016 indsat pneumoni i alle ICS produktresumeer som en normal bivirkning (1-10 %) *[5]*. I Danmark er der ca. 150.000 patienter diagnosticeret med KOL og det estimeres af ca. 30-70 % af dem er i behandling med ICS*[3][4][6][7]*. Derfor er det relevant at se på, om fortsat behandling med ICS kan begrænses og forbeholdes en mindre gruppe af patienter med KOL.

Formål med denne retningslinje er at vejlede sundhedspersonale om hvilke patienter med KOL, der vil have glæde af fortsat behandling med inhalationssteroid (ICS) og reducere brugen af ICS blandt patienter som ikke har glæde af denne behandling. Dette vurderes ud fra mulige fordele såvel som ulemper af fortsat behandling med ICS fundet i den forhåndenværende litteratur.

Afgrænsning af patientgruppe

Denne kliniske retningslinje omhandler patienter med KOL på 40 år eller derover, som ikke er nuværende astmapatienter, og som har været i behandling med ICS i mindst 3 måneder,

Målgruppe/brugere

Målgruppen for denne nationale kliniske retningslinje er helt overvejende almen praksis, herunder også sygeplejersker og andet sundhedspersonale ansat i almen praksis, samt lungemedicinere og lungesygeplejersker. Sekundært henvender den sig til eventuelle andre læger, sygeplejersker, farmaceuter og ander personale, der varetager kontrol, behandling og rådgivning af patienter med KOL i behandling med ICS.

Patienter med KOL og pårørende kan ligeledes orientere sig i retningslinjen

Emneafgrænsning

Den nationale kliniske retningslinje indeholder handlingsanvisninger for udvalgte og velafgrænsede kliniske problemstillinger ('punktnedslag i patientforløbet'). Disse problemstillinger er prioriteret af den faglige arbejdsgruppe som de områder, hvor det er vigtigst at få afklaret evidensen. Denne nationale kliniske retningslinjer beskæftiger sig således med udvalgte dele af behandlingsindsatsen.

Fokus for denne retningslinje er hvilke af de nuværende patienter med KOL i behandling med ICS, som vurderes at have gavn af henholdsvisat fortsætte og at ophøre denne behandling. Arbejdsgruppen har valgt at undersøge dette på baggrund af patienternes blod-eosinofilocytter, exacerbationshistorie og pneumonihistorie.

Patientperspektivet

Patienternes værdier og præferencer er vigtige i fortsat behandling såvel som ophør med behandling. I denne retningslinje er patientperspektivet repræsenteret ved at inddrage Lungeforeningen, som har udpeget deres formand til at sidde med i arbejdsgruppen. Se medlemmerne af arbejds- og referencegruppen i bilag 8. Lungeforeningen og Danske Patienter vil desuden blive inddraget i høringsfasen.

3 - Patienter med KOL i behandling med ICS i forhold til EOS niveauer

Baggrund for PICO 1-3

Inhalationssteroid (ICS) har antiinflammatorisk virkning, som formodes at være virkningsmekanismen ved behandling af patienter med kronisk obstruktiv lungesygdom (KOL) *[8]*. Studier har vist, at niveauet af eosinofilocytter i sputum kan korreleres til graden af inflammation i lungerne *[9]* og at niveauet af eosinofilocytter i sputum hos patienter med KOL kan være prædiktiv for effekten af systemisk steroid såvel som inhalationssteroid *[8][10][11][12][13]*. Målinger af eosinofilocytter i sputum er en omstændelig proces, der ikke vil være rutinemæssig mulig de fleste steder - herunder i almen praksis. Derfor bruges eosinofilocytter i blod (EOS) i denne retningslinje og i de fleste studier. EOS måles gennem en blodprøve og er derfor en let tilgængelig og billig måde at måle inflammation på.

Ved exacerbationer som følge af KOL har studier vist, at patienter med EOS over 2 % responderer bedre på behandling med systemisk steroid, end patienter med EOS under 2 % *[14][12]*. I Danmark, og de fleste andre steder, anvendes det absolutte antal EOS (celler/µL), i stedet for det relative EOS. Når der bestilles leukocytter med differentialtælling er det således det absolutte antal EOS, der oplyses fra laboratoriet. Global Initiative for Chronic Obstructive Lung Disease (GOLD) har siden 2019 *[15]* anbefalet at bruge absolut EOS til at vurdere, om patienter med svær KOL og hyppige exacerbationer skal opstartes i behandling med ICS.

Studier viser, at EOS kan påvirkes af forskellige faktorer, som f.eks. samtidig medicin, infektion, exacerbation og tobak *[18][19][20][21]* . Derfor kan det være relevant at overveje hvornår EOS måles/er målt. Endvidere ser det ud til, at der hos raske, såvel som hos patienter med KOL, kan være variation i EOS livet igennem *[18][19][22]* .

For patienter med KOL, som er i behandling med ICS, virker EOS derfor som en mulig relevant biomarkør at inddrage i beslutningsprocessen om behandling med ICS skal fortsættes eller seponeres. Dog er der endnu ikke konsensus om grænseværdier. Et studie peger på at patienter med en EOS på over 300 celler/uL har effekt af behanding med ICS, hvorimod patienter med EOS på under 100 celler/uL har beskeden effekt *[16]*. Et dansk studie på en repræsentativ befolkningsgruppe fra København (i forhold til køn og alder) fandt, at den gennemsnitlige EOS var 180 celler/µL for patienter med KOL *[17]*. Arbejdsgruppen har derfor valgt at undersøge effekten af forsat behandling af ICS til patienter med KOL og med EOS på hhv. under 150, mellem 150-300 og over 300 celler/µL.

3.1 - Patienter med KOL i behandling med ICS og med EOS under 150 celler/ µL

PICO 1

Bør patienter med kronisk obstruktiv lungesygdom (KOL) og blod-eosinofilocytter (EOS) under 150 celler/ µL fortsat behandles med inhalationssteroid (ICS)?

Svag anbefaling mod

Fortsæt kun behandling med inhalationssteroid (ICS) til patienter med kroniske obstruktiv lungesygdom (KOL) og blod-eosinofilocytter (EOS) under 150 celler/µL efter nøje overvejelser.

Patienter med KOL og EOS under 150 celler/µL forventes kun at have sparsom effekt af fortsat behandling med ICS.

Studierne viser en reduktion af risiko for moderat til svær exacerbation på 12 % (konfidensinterval 6-17 %). Hvis patienten fx har en moderat til svær exacerbation om året, vil dette svare til én exacerbation mindre i løbet af 10 år. Patienten vil stadig rammes af de 9 andre exacerbationer.

Arbejdsgruppen anbefaler på baggrund af klinisk erfaring at seponere ICS gradvist i samarbejde med patienten. Fx reducér til halv dosis og fortsæt i 6-8 uger efterfulgt af seponering. Kan patienten ikke undvære ICS, anbefaler arbejdsgruppen, at behandlingen genoptages i lavest mulige effektive dosis. Se i øvrigt DLS' KOL vejledning.

Nøgleinformationer

Gavnlige og skadelige virkninger — Lille netto gevinst eller små forskelle mellem alternativerne

Fortsat behandling med ICS nedsætter sandsynligvis risiko for *moderat til svær exacerbation* (rate ratio: 0,88 (95 % CI :0,83 - 0,94)). Resultatet vurderes ikke klinisk relevant. Arbejdsgruppen har forud for litteraturgennemgangen sat mindste kliniske relevante forskel til 25 %. Tiltroen til estimatet er moderat pga. risiko for bias i studierne.

Fortsat behandling med ICS nedsætter muligvis *frafald af alle årsager* i studierne (risk ratio: 0,78 (95 % CI: 0,73 - 0,84)). Tiltroen til estimatet er lav pga. risiko for bias og manglende overførbarhed. Ingen studier opgjorde *frafald af alle årsager* ud fra EOS og estimatet gælder for alle deltagere i studierne uanset EOS.

Fortsat behandling med ICS påvirker sandsynligvis ikke *antal patienter, der får alvorlige bivirkninger* (risk ratio: 0,96 (95 % CI: 0,9 - 1,03)). Tiltroen til estimatet er moderat pga. risiko for bias.

Fortsat behandling med ICS påvirker muligvis ikke *lungefunktionen* (FEV1 mean difference: 0,02 L (95 % CI: 0 - 0,05 L)). Tiltroen til estimatet er lavt pga. risiko for bias og alvorlige inkonsistente resultater. Der er ikke fundet studier, som opgjorde *antal patienter med en væsentlig forbedring i lungefunktion* og det er arbejdsgruppens vurdering at fortsat behandling med ICS har ingen eller ubetydelig påvirkning af dette outcome.

Fortsat behandling med ICS øger sandsynligvis risiko for *pneumoni* (risk ratio: 1,39 (95 % CI: 1,19 - 1,63)). Tiltroen til estimatet er moderat pga. risiko for bias.

Det er usikkert om fortsat behandling med ICS påvirker *dyspnø* (TDI-skala mean difference: 0,11 (95 % CI: -0,58 - 0,37) og *livskvalitet* (SGRQ-skala mean difference: 1,2 (95 % CI: -2,87 - 0,46)). Resultaterne vurderes ikke klinisk relevante, da mindste klinisk relevante forskel er sat til hhv. 1 *[71]* og 4 *[70]*. Tiltroen til estimaterne er meget lavt pga. risiko for bias, upræcist effektestimat og for *livskvalitet* også inkonsistente resultater.

Kvaliteten af evidensen — Lav

Der er moderat tiltro til evidensen for det kritiske outcome *moderate og svære exacerbationer*. Tiltroen til evidensen er nedgraderet pga. alvorlig risiko for bias som følge af utilstrækkeligt skjult randomisering, selektiv rapportering af outcome (næsten alle studier har subgruppeanalyseret på EOS som en post-hoc analyse), samt gennemgående indblanding fra medicinalindustrien i forsøgsdesign og rapportering af resultater.

Der er ligeledes moderat tiltro til evidensen for det kritiske outcome *alvorlige bivirkninger*. Tiltroen til evidensen er nedgraderet til moderat pga. alvorlig risiko for bias som følge af utilstrækkeligt skjult randomisering samt gennemgående indblanding fra medicinalindustrien i forsøgsdesign og rapportering af resultater. *Alvorlige bivirkninger* er ikke opgjort i EOS subgrupper, men her vurderes det, at EOS ikke har indflydelse.

Der er lav tiltro til evidensen for det kritiske outcome *frafald af alle årsager*. Tiltroen til evidensen er nedgraderet pga. alvorlig risiko for bias og alvorlig manglende overførbarhed. Risiko for bias skyldes utilstrækkeligt skjult randomisering, samt gennemgående indblanding fra medicinalindustrien i forsøgsdesign og rapportering af resultater. *Frafald af alle årsager* er ikke opgjort i EOS subgrupper, hvilket resulterer i manglende overførbarhed mellem den undersøgte population og målpopulationen for PICO. Særligt ved frafald vurderes det, at EOS har indflydelse. Frafald pga. manglende effekt, vil forventes at være større ved høj EOS og mindre ved lav EOS.

For de vigtige outcomes er kvaliteten moderat til meget lav (se evidensprofilen for information på de enkelte outcomes). Tiltroen til evidensen er nedgraderet pga. alvorlig risiko for bias, alvorlige inkonsistente resultater med uforklarlig varians, få studier og brede konfidensintervaller. Risiko for bias skyldes utilstrækkeligt skjult randomisering, selektiv rapportering af outcome (næsten alle studier har subgruppeanalyseret på EOS som en post-hoc analyse), samt risiko for bias pga. gennemgående indblanding i forsøgsdesign og rapportering af resultater fra medicinalindustrien.

Samlet set er kvaliteten af evidensen lav.

Patientpræferencer

Ingen betydelig variation forventet

Det er arbejdsgruppens vurdering, at de fleste patienter ud fra en samlet vurdering af fordele og ulemper i samråd med læge vil være interesserede i at ophøre med ICS behandling.

Rationale

Der blev i formuleringen af anbefalingen lagt vægt på at fortsat behandling med ICS har en begrænset effekt på risiko for moderat til svær exacerbation, og at der ikke er flere patienter med alvorlige bivirkninger ved fortsat behandling med ICS. Dette sammenholdes med, at seponering af ICS ikke forværrer dyspnø, lungefunktion eller livskvalitet og at seponering af ICS mindsker risiko for pneumoni. Der forventes ingen betydelig variation i patientpræferencer.

Fokuseret Spørgsmål

Population: Patienter på 40 år eller derover med KOL, som har EOS under 150 celler/µL, ikke er nuværende astmapatienter og som har været i behandling med ICS mindst 3 måneder inden og/eller under forsøget.

Intervention: Behandling med ICS dagligt i mindst 3 måneder (gælder både nyopstartet eller fortsat behandling med ICS) - enten som monoterapi eller i tilgift til behovsmedicin eller anden inhalationsbehandling (ikke ICS).

Sammenligning: Ingen ICS-behandling (men kan have fået ICS-behandlingen forud for forsøgsopstart). Patienter kan være i behandling med behovsmedicin eller få anden inhalationsbehandling (ikke ICS).

Sammenfatning

Denne sammenfatning omhandler PICO 1-3

Litteratursøgning

Der blev identificeret ét systematisk review *[23]* med lidt andre inklusionskriterier end PICO 1-3. Herfra kunne 8 studier (både inkluderede og ekskluderede i reviewet) *[16][24][25][26][27][28][29][30]* inkluderes i PICO 1-3. Der blev fortaget en primærlitteratursøgning fra 2010 til 2020 juni, hvor yderligere 2 studier blev identificeret, hhv. ét publiceret studie *[31]* og ét konferenceabstract *[32]* (relevant data ikke publiceret andet sted). I den efterfølgende litteraturgennemgangen af de øvrige PICO 4 og 5 blev et nypubliceret primærstudie *[33]* (publiceret i juli 2020) identificeret.

Evidensgrundlaget for PICO 1 er 11 randomiserede forsøg *[16][24][25][26][27][28][29][30][31][32][33]*.

Evidensgrundlavet for PICO 2 og 3 er 6 randomiserede forsøg *[16][24][28][29][30][32]*.

Flow charts findes her.

Gennemgang af evidensen

De 11 inkluderede studier var randomiserede kontrollerede forsøg. Interventionerne bestod af ICS i kombinationer med LABA eller både LABA og LAMA og sammenligningsgrupper var hhv. enten LABA alene eller i kombination med LAMA (se tabel). Varigheden af interventionerne varierede mellem 24 til 52 uger. På tværs af studierne var patienterne primært mænd (60-80 %), med en aldersspredning på 63 til 65,3 år, body mass index (BMI) mellem 25,11 til 27,98 kg/m2, mellem 40-70 % var nuværende rygere og patienternes sværhedsgrad af KOL bedømt ud fra lungefunktionsnedsættelse målt ved FEV1(GOLD-klassifikationer) var tilsammen: 0 % mild, 32 % moderat, 52 % svær og 16 % meget svær. To studier *[27][28]* havde ikke opgivet sværhedsgraden af lungefunktionsnedsættelsen.

Tabel 1: Oversigt over inkluderede studier i PICO 1-3

Studie	ICS inden RUN-IN	RUN-IN	Intervention(er)	Sammenligning
**Bafadhel 2018** *[16]*	63 %	2 uger. Fortsæt ICS under.	**ICS+LABA x 2 dgl.**<br>Budesonide 160µg+formoterol 4,5µg eller budesonide 80µg+formoterol 4,5µg	**LABA x 2 dgl.**<br>Formoterol 4,5µg
**ETHOS** *[33]*	80 %	1-4 uger. Fortsæt ICS under.	**ICS+LABA+LAMA x 2 x 2 dgl.**<br>Budesonide 160µg + formoterol 4,8µg + glycopyrrolate 9µg<br>eller<br>budesonide 80µg + formoterol 4,8µg + glycopyrrolate 9µg	**LABA+LAMA x 2 x 2 dgl.**<br>Formoterol 4,8µg+ glycopyrrolate 9µg
**FORWARD** *[28]*	ukendt	2 uger LABA.	**ICS+LABA x 2 x 2 dgl.**<br>Beclometason 100µg + formoterol 6µg	**LABA x 2**<br>Formoterol 12µg
**IMPACT** *[30]*	71 %	2 uger, fortsæt vanlig behandling.	**ICS+LAMA+LABA x 1 dgl.**<br>Fluticason 100µg + umeclidinium 65,5µg + Vilanterol 25µg	**LAMA+LABA x 1 dgl.**<br>Umeclidinium 62,5µg + vilanterol 25µg
**INSTEAD** *[32]*	100 %	Ingen	**ICS + LABA x 2 dgl.**<br>Fluticason 500µg + salmeterol 50µg	**LABA x 1**<br>Vilanterol µg
**KRONOS** *[25]*	72 %	Ingen	**ICS+LAMA+LAMA x 2**<br>budesonide 320µg + glycopyrrolate 18µg + formoterol 9,6µg	**LABA+LAMA x2**<br>glycopyrrolate 18µg + formoterol 9,4µg
**Pascoe 2015** *[27]*	71 %	4 uger ICS+LABA.	**ICS+LABA x 1 dgl.**<br>Fluticaton 50+vilanterol 25<br>eller<br>Fluticaton 100+vilanterol 25<br>eller<br>Fluticaton 200+vilanterol 25	**LABA x 1 dgl.**<br>Vilanterol 25µg
**SOPHOS** *[31]*	76 %	Ingen	**ICS+LABA x 2 dgl.**<br>Budesonide 320µg + formoterol 10µg<br>eller<br>Budesonide 160µg + formoterol 10µg	**LABA x 2 dgl.**<br>Formoterol 10µg
**SUNSET** *[24]*	100 %	1 mdr. ICS+LABA+LAMA	**ICS+LABA x 2 dgl. +LAMA x 1 dgl.**<br>Fluticason propionate 500µg + samleratol 50µg + tiotropium 18µg	**LABA+LAMA x 1 dgl.**<br>Indacaterol 110µg + glycopyrronium 50µg
**TRIBUTE** *[26]*	65 %	2 uger LABA+LAMA	**ICS+LABA+LAMA x 2 x 2 dgl.**<br>Budesonid 87µg + formoterol 5µg + glycopyrronium 9µg	**LABA+LAMA** x 1 dgl.<br>Indacaterol 85µg + glycopyrronium 43µg
**WISDOM** *[29]*	70 %	6 uger ICS+LABA+LAMA	**ICS+LABA x 2 dgl. +LAMA x 1 dgl.**<br>Fluticason propionate 500µg + salmeterol 50µg + tiotropium 18µg	**LABA x 2 dgl. +LAMA x 1 dgl. (stepwise reduction of ICS)**<br>Salmeterol 50µg + tiotropium 18µg

<u>Brug af ICS inden inklusion og randomisering</u>
Få studier overholdt kravet om 3 måneders behandling med ICS til alle patienter inden randomisering. Derfor valgte arbejdsgruppen at medtage studier, hvor over 50 % af inkluderede patienter var i behandling med ICS inden studiestart og, hvor en eventuel ICS fri run-in perioder var maks. 2 uger.

To studier overholdt kravet om 3 måneders behandling med ICS til alle patienter inden randomisering (INSTEAD og SUNSET). Se **tabel 1** for yderligere information om forudgående ICS behandling og run-in periode. I sensitivitetsanalyser, hvor vi undersøgte om effektestimatet i INSTEAD og SUNSET adskilte sig fra de øvrige studier, fandt vi ingen forskel i test for subgrupperne (INSTEAD+SUNSET mod andre: exacerbationer p=0,41; lungefunktion p=0,66; serious adverse events p=0,30; dropout p=0,98).

Grænser for blod-eosinofilocytter

Arbejdsgruppen valgte at inkludere studier som havde andre EOS grænser end 150, og 300. Af de 11 studier, vi brugte til PICO 1, havde tre en anden EOS afgrænsning end <150 celler/mikroL, hhv. FORWARD (<181,6), TRIBUTE (<200) og IMPACT (<90 og 90-140). Af de 6 studier vi brugte i PICO 2 og 3 havde 2 en anden EOS afgrænsning end 150 og 300: FORWARD (181,6-279,8) og IMPACT (140-200 og 200-310). Studierne har målt EOS ved baseline, hvor patienterne har været stabile i mindst 4 uger.

Pneumoni/exacerbation

Alle definitioner af exacerbation og pneumoni fra studierne er inkluderet.

Resultater

Fortsat behandling med ICS nedsætter sandsynligvis risiko for det kritiske outcome *moderat til svær exacerbation* uanset EOS. Der er d og usikkert om resultatet for d e enkelte PICO er klinisk relevant. Arbejd sgruppen har forud for litteraturgennemgangen sat mindste kliniske relevante forskel til 25 %. For patienter med EOS under 150 celler/µL (PICO 1) er reduktionen 12 % (rate ratio: 0,88; 95 % CI: 0,83 - 0,94). For patienter med EOS mellem 150-300 celler/µL (PICO 2) er reduktionen 20 % (rate ratio: 0,80; 95 %CI: 0,69 - 0,94). For patienter med EOS over 300 celler/µL (PICO 3) er der tale om en væsentlig reduktion på 43 % (rate ratio: 0.57; 95 %CI: 0,49 – 0,66). Test af forskelle i subgrupper med hhv. EOS under 150 celler/µL, mellem 150-300 celler/µL og over 300 celler/µL, viser at der er væsentlig forskel på reduktionen af *risiko for moderat til svær exacerbation* på de tre grupper (p<0,00001). Arbejdsgruppen vurderer, at det ikke er en fordel, i forhold til *risiko for moderat til svære exacerbationer*, at forsætte med ICS for patienter med EOS under 150 celler/µL, men at det kan være for patienter med EOS over 150 celler/µL. Der er moderat tiltro til evidensen, idet der blev nedgraderet pga. alvorlig risiko for bias som følge af utilstrækkeligt skjult randomisering, selektiv rapportering af outcome (næsten alle stud ier har subgruppeanalyseret på EOS som en post-hoc analyse), samt gennemgående indblanding fra medicinalindustrien i forsøgsdesign og rapportering af resultater. For EOS mellem 150-300 celler/µ er der ikke blevet nedgraderet for inkonsistens, selvom I^2 er 63%. Det skyldes, at alle studier favoriserede behandlingsgruppen i mindre eller større grad, og at de inkonsistente resultater dermed var mellem studier der viste moderat til store eller meget store effekter. Den observerede heterogenitet forventes derfor ikke at have en væsentlig indvirkning på de vurderingsparametre der ligger til grund for anbefalingen.

Fortsat behandling med ICS nedsætter muligvis det kritiske outcome *frafald af alle årsager* i studierne (risk ratio: 0,78; 95 % CI: 0,73-0,84). Frafald af alle årsager er ikke opgjort i EOS subgrupper, hvilket resulterer i manglende overførbarhed mellem populationen outcome er opgjort på og population PICO-spørgsmålet omhandler. Særligt ved frafald vurderes det, at EOS har indflydelse. Frafald pga. manglende effekt, vil forventes at være større ved høj EOS og lavere ved lav EOS. Tiltroen til estimatet er lav pga. alvorlig risiko for bias og alvorlig manglende overførbarhed. Risiko for bias skyldes utilstrækkeligt skjult randomisering, samt gennemgående indblanding fra medicinalindustrien i forsøgsdesign og rapportering af resultater.

Fortsat behandling med ICS påvirker sandsynligvis ikke det kritiske outcome *antal patienter, der får alvorlige bivirkninger* (risk ratio: 0,96; 95 % CI: 0,9 - 1,03). Alvorlige bivirkninger er ikke opgjort i EOS subgrupper, men her vurderes det, at EOS ikke har indflydelse. Tiltroen til evidensen er nedgraderet til moderat pga. alvorlig risiko for bias som følge af utilstrækkeligt skjult randomisering samt gennemgående indblanding fra medicinalindustrien i forsøgsdesign og rapportering af resultater.

Fortsat behandling med ICS øger sandsynligvis risikoen for det vigtige outcome *pneumoni* opgjort uafhængigt af EOS subgrupper (risk ratio: 1,39; 95 % CI: 1,19 - 1,63). Tiltroen til estimatet er moderat pga. alvorlig risiko for bias. Risiko for bias skyldes utilstrækkeligt skjult randomisering, samt gennemgående indblanding fra medicinalindustrien i forsøgsdesign og rapportering af resultater.

Fortsat behandling med ICS for patienter med EOS under 150 celler/µL påvirker sandsynligvis ikke *lungefunktion*, som er et vigtigt outcome (FEV1 mean difference: 0,02 L; 95 % CI: 0,00 - 0,05 L). For patienter med EOS hhv. mellem 150-300 celler/µL (PICO 2) og over 300 celler/µL (PICO 3) forbedre forsat behandling med ICS *lungefunktion* med hhv. 0,05 L(FEV1 mean difference; 95 % CI: 0,03 - 0,07 L) og 0,06 L (FEV1 mean difference; 95 % CI: 0,04 - 0,09 L). Test af forskelle i subgrupper med hhv. EOS under 150 celler/ µL, mellem 150-300 celler/µL og over 300 celler/µL, viser at der er en forskel på hvordan fortsat behandling med ICS påvirker *lungefunktion* på tværs af subgrupperne (p=0,04). Arbejdsgruppen vurderer at der er en større fordel, i forhold til *lungefunktion*, ved forsat behandling med ICS hos patienter med EOS over 150 celler/µL (PICO 2 og 3) end hos patienter med EOS under 150 celler/µL (PICO 1). Tiltroen til estimatet er moderat pga.

alvorlig risiko for bias, idet der var utilstrækkeligt skjult randomisering, samt gennemgående indblanding fra medicinalindustrien i forsøgsdesign og rapportering af resultater. Der er ikke fundet studier, som opgjorde antal patienter med en væsentlig forbedring i lungefunktion og det er arbejdsgruppens vurdering at fortsat behandling med ICS ikke har betydelig påvirkning af dette outcome.

Det er usikker på om fortsat behandling med ICS påvirker det vigtige outcome *dyspnø* på tværs af EOS. Ingen af resultaterne er klinisk relevante (mindste klinisk relevante forskel er sat til 1 *[71]*). Patienter med EOS på hhv. under 150 celler/µL (PICO 1), 150-300 celler/µL (PICO 2) og over 300 celler/µL (PICO 3) havde en ændring på hhv. 0,11 (TDI-skala mean difference; 95 % CI: -0,58 - 0,37), 0,13 (TDI-skala mean difference; 95 % CI: -0,29 - 0,55) og 0,3 (TDI-skala mean difference; 95 % CI: -0,37 - 0,97). Test af forskelle i subgrupper med hhv. EOS under 150 celler/ µL, mellem 150 og 300 celler/µL og over 300 celler/µL viser, at resultatet for *dyspnø* forventes at være det samme uanset EOS niveau (p=0,59). Tiltroen til estimaterne er meget lavt pga. alvorlig risiko for bias, idet der var utilstrækkeligt skjult randomisering, samt gennemgående indblanding fra medicinalindustrien i forsøgsdesign og rapportering af resultater og pga. meget alvorligt upræcist effektestimat, da der kun er data fra ét studie og brede konfidensintervaller.

For det vigtige outcome *livskvalitet* er det usikkert om der er fordel af fortsat behandling med ICS for patienter med EOS under 150 celler/µL (SGRQ-skala mean difference: 1,20; 95 % CI: -2,87 - 0,46). Der er ikke betydelig forbedring af livskvalitet ved fortsat behandling med ICS for patienter med EOS mellem 150 og 300 celler/µL SGRQ-skala mean difference: 1,80; 95 % CI: 3,01 - 0,58), mens det muligvis medfører en forbedring af livskvalitet for patienter med EOS over 300 celler/µL (SGRQ-skala mean difference: 3,98; 95 % CI: 6,96 - 1,01). Mindste klinisk relevante forskel er sat til 4 *[70]*. På baggrund af en test af forskelle i subgrupper inddelt i hhv. EOS under 150 celler/ µL, mellem 150 og 300 celler/µL og over 300 celler/µL, kan det ikke afvises at effekten er ens på tværs af EOS niveau (p=0,28). Tiltroen til effektestimatet er lav for PICO 2 og 3 pga. alvorlig risiko for bias, idet der var utilstrækkeligt skjult randomisering, samt gennemgående indblanding fra medicinalindustrien i forsøgsdesign og rapportering af resultater, og pga. alvorlig upræcist effektestimat. For PICO 1 er tiltroen meget lav pga. yderligere alvorlig inkonsistente resultater.

**Outcome**<br>Tidsramme	**Resultater og målinger**	**EffX¦ºX´ºi ª Jter**<br>Seponering	ICS	**Tiltr- ºiꞵ X´ºi ª Jterne**<br>(at de afspejler den sande effekt i populationen)	**Sammendrag**
**Alvorlige bivirkninger, alle EOS (serious adverse events, number of patients, all EOS)**<br>Længst mulige follow-up (min. 3 mdr. fra intervention start)<br><br>9 Kritisk	Relative risiko 0.96<br>(CI 95% 0.9 - 1.03)<br>Baseret på data fra 29,654 patienter i 11 studier. [1]<br>(Randomiserede studier)<br>Opfølgningstid: 12-52 uger.	**178**<br>per 1.000	**171**<br>per 1.000	**Moderat**<br>på grund af alvorlig risiko for bias [2]	Fortsat behandling med ICS påvirker sandsynligvis ikke antallet af patienter som oplever alvorlige bivirkninger.
		Forskel: **7 færre** per 1.000<br>18 færre - 5 flere			
**Frafald af alle årsager, alle EOS (All-cause drop-out, all EOS)**<br>Længst mulige follow-up (min. 3 mdr. fra intervention start)<br><br>9 Kritisk	Relative risiko 0.78<br>(CI 95% 0.73 - 0.84)<br>Baseret på data fra 29,723 patienter i 11 studier. [3]<br>Opfølgningstid: 12-52 uger.	**233**<br>per 1.000	**182**<br>per 1.000	**Lav**<br>på grund af alvorlig risiko for bias, på grund af alvorlig manglende overførbarhed [4]	Fortsat behandling med ICS nedsætter muligvis frafald af alle årsager
		Forskel: **51 færre** per 1.000<br>63 færre - 37 færre			

**Outcome** Tidsramme	**Resultater og målinger**	**EffX¦ºX´ºi ª Jter** Seponering / ICS	**Tiltr- ºiȣ X´ºi ª Jterne** (at de afspejler den sande effekt i populationen)	**Sammendrag**
**Antal patienter med en væsentlig forbedring i lungefunktion (FEV1) (number of patients with improvement in lung function)** Længst mulige follow-up (min. 3 mdr. fra intervention start) 6 Vigtig	Baseret på data fra patienter i 0 studier. [5]			Vi fandt ingen studier der opgjorde antal patienter med forbedring i lungefunktion. Det er arbejdsgruppens kliniske vurdering at fortsat behandling med ICS muligvis har ingen eller ubetydelig forskel.
**Pneumoni, antal patienter, alle EOS (Pneumonia, number of patients, all EOS)** Længst mulige follow-up (min. 3 mdr. fra intervention start) 6 Vigtig	Relative risiko 1.39 (CI 95% 1.19 - 1.63) Baseret på data fra 29,654 patienter i 11 studier. [6] (Randomiserede studier) Opfølgningstid: 12-52 uger.	Seponering: **32** per 1.000; ICS: **44** per 1.000. Forskel: **12 flere** per 1.000 6 flere - 20 flere	**Moderat** på grund af alvorlig risiko for bias [7]	Fortsat behandling med ICS øger sandsynligvis risikoen for pneumoni
**Pneumoni rate (pneumonia rate)** Længst mulige follow-up (min. 3 mdr. fra intervention start) 6 Vigtig	Lavere bedre Baseret på data fra: 2,983 patienter i 2 studier. [8] (Randomiserede studier) Opfølgningstid: 12-52 uger.	Forskel: **MD 0.08 højere** (CI 95% 0.06 højere - 0.1 højere)	**Moderat** på grund af alvorlig risiko for bias [9]	Fortsat behandling med ICS øger sandsynligvis risikoen for pneumoni
**Dyspnø (Dyspnea)** Længst mulige follow-up (min. 3 mdr. fra intervention start) 6 Vigtig	Målt med: TDI Skala: -9-9 Højere bedre Baseret på data fra: 2,480 patienter i 1 studier. [10] (Randomiserede studier) Opfølgningstid: 52 uger.	Forskel: **MD 0.11 lavere** (CI 95% 0.58 lavere - 0.37 højere)	**Meget lav** på grund af alvorlig risiko for bias, på grund af meget alvorlig upræcist effektestimat [11]	Vi er usikre på, om fortsat behandling med ICS forbedrer dyspnø

**Outcome** Tidsramme	**Resultater og målinger**	**EffX¦ºX´ºiª Jter** Seponering	ICS	**Tiltr- ºiꝗ X´ºiª Jterne** (at de afspejler den sande effekt i populationen)	**Sammendrag**
**Lungefunktion, (lung function)** Længst mulige follow-up (min. 3 mdr. fra intervention start) 6 Vigtig	Målt med: FEV1 Højere bedre Baseret på data fra: 5,568 patienter i 6 studier. [12] (Randomiserede studier) Opfølgningstid: 12-52 uger.	0.01 (gennemsnit)	0.03 (gennemsnit)	**Lav** på grund af alvorlig risiko for bias, på grund af alvorlig inkonsistente resultater [13]	Fortsat behandling med ICS påvirker muligvis ikke lungefunktion
		Forskel: MD 0.02 højere (CI 95% 0 lavere - 0.05 højere)			
**Livskvalitet (quality of life)** Længst mulige follow-up (min. 3 mdr. fra intervention start) 6 Vigtig	Målt med: SGRQ Skala: 0-100 Lavere bedre Baseret på data fra: 2,983 patienter i 2 studier. [14] (Randomiserede studier) Opfølgningstid: 48-52 uger.	46.8 (gennemsnit)	45.6 (gennemsnit)	**Meget lav** på grund af alvorlig risiko for bias, på grund af alvorlig inkonsistente resultater, på grund af alvorlig upræcist effektestimat [15]	Vi er usikre på, om fortsat behandling med ICS forbedrer livskvalitet
		Forskel: MD 1.2 lavere (CI 95% 2.87 lavere - 0.46 højere)			
**Moderat til svær exacerbation (exacerbations)** [16] Længst mulige follow-up (min. 3 mdr. fra intervention start) 9 Kritisk	Baseret på data fra: patienter i 11 studier. Opfølgningstid: 12-52 uger.	Rate Ratio: 0.88 (CI% 95% 0.83 - 0.94)		**Moderat** på grund af alvorlig risiko for bias [17]	Fortsat behandling med ICS påvirker sandsynligvis ikke antallet af patienter som oplever alvorlige bivirkninger.

1. Systematisk oversigtsartikel [34] med inkluderede studier: [26], [25], [16], [33], [32], [31], [30], [29], [28], [27], [24]. **Baselinerisiko/komparator:** Kontrolarm i reference brugt til interventionen.
2. **Risiko for bias: Alvorligt.** Utilstrækkeligt skjult randomisering, risk of bias på grund af gennemgående indblanding i forsøgsdesign og rapportering af resultater fra medicinalindustrien. **Inkonsistente resultater: Ingen betydelig. Manglende overførbarhed: Ingen betydelig.** Ikke alle patienter havde ICS inden randomisering, men størstedelen havde og der ser ikke ud til at være en stor forskel mellem de studier hvor alle fik ICS inden intervention og dem hvor de fleste fik ICS inden

intervention. Dette outcome er ikke opgjort på baggrund af blodeosinofiltallet, men for hele populationen i interventions- og comparisongruppen.. **Upræcist effektestimat: Ingen betydelig. Publikationsbias: Ingen betydelig.**

3. Systematisk oversigtsartikel [34] med inkluderede studier: [33], [30], [26], [25], [31], [24], [29], [32], [28], [27], [16]. **Baselinerisiko/komparator:** Kontrolarm i reference brugt til interventionen.

4. **Risiko for bias: Alvorligt.** Utilstrækkeligt skjult randomisering og risk of bias på grund af gennemgående indblanding i forsøgsdesign og rapportering af resultater fra medicinalindustrien.. **Inkonsistente resultater: Ingen betydelig.** Den statistiske heterogenicitet er høj (56%), men da de alle favoriserer ICS er der ikke trukket ned.. **Manglende overførbarhed: Alvorligt.** Forskelle mellem målpopulation og studiepopulation, da det ikke er subgruppeanalyseret på EOS. **Upræcist effektestimat: Ingen betydelig. Publikationsbias: Ingen betydelig.**

5. Systematisk oversigtsartikel [34] . **Baselinerisiko/komparator:** Kontrolarm i reference brugt til interventionen.

6. Systematisk oversigtsartikel [34] med inkluderede studier: [30], [16], [24], [25], [26], [27], [29], [28], [33], [32], [31]. **Baselinerisiko/komparator:** Kontrolarm i reference brugt til interventionen.

7. **Risiko for bias: Alvorligt.** Utilstrækkeligt skjult randomisering, risk of bias på grund af gennemgående indblanding i forsøgsdesign og rapportering af resultater fra medicinalindustrien.. **Inkonsistente resultater: Ingen betydelig. Manglende overførbarhed: Ingen betydelig.** Ikke alle patienter havde ICS inden randomisering, men størstedelen havde og der ser ikke ud til at være en stor forskel mellem de studier hvor alle fik ICS inden intervention og dem hvor de fleste fik ICS inden intervention. Dette outcome er ikke opgjort på baggrund af blodeosinofiltallet, men for hele populationen i interventions- og comparisongruppen. 2 studier opgjorde det for patienter med et blodeosinofiltal under 150 og her var samme tendens om end tydeligere. Studierne er lavet på forskellige typer af ICS, som muligvis har forskellig risiko for at give pneumoni.. **Upræcist effektestimat: Ingen betydelig. Publikationsbias: Ingen betydelig.**

8. Systematisk oversigtsartikel [34] med inkluderede studier: [28], [30]. **Baselinerisiko/komparator:** Kontrolarm i reference brugt til interventionen.

9. **Risiko for bias: Alvorligt.** Utilstrækkeligt skjult randomisering, Selektiv rapportering af outcome og risk of bias på grund af gennemgående indblanding i forsøgsdesign og rapportering af resultater fra medicinalindustrien.. **Inkonsistente resultater: Ingen betydelig.** Den statistiske heterogenicitet er høj, men da de alle favoriserer seponering er der ikke trukket ned.. **Manglende overførbarhed: Ingen betydelig. Upræcist effektestimat: Ingen betydelig. Publikationsbias: Ingen betydelig.**

10. Systematisk oversigtsartikel [34] med inkluderede studier: [30]. **Baselinerisiko/komparator:** Kontrolarm i reference brugt til interventionen.

11. **Risiko for bias: Alvorligt.** Utilstrækkeligt skjult randomisering, Selektiv rapportering af outcome, risk of bias på grund af gennemgående indblanding i forsøgsdesign og rapportering af resultater fra medicinalindustrien.. **Inkonsistente resultater: Ingen betydelig. Manglende overførbarhed: Ingen betydelig.** kun 71 % af patienterne fik ICS inden randomisering.. **Upræcist effektestimat: Meget alvorligt.** Kun data fra ét studie, Brede konfidensintervaller. **Publikationsbias: Ingen betydelig.**

12. Systematisk oversigtsartikel [34] med inkluderede studier: [24], [31], [30], [29], [28], [25]. **Baselinerisiko/komparator:** Kontrolarm i reference brugt til interventionen.

13. **Risiko for bias: Alvorligt.** Utilstrækkeligt skjult randomisering, Selektiv rapportering af outcome, risk of bias på grund af gennemgående indblanding i forsøgsdesign og rapportering af resultater fra medicinalindustrien.. **Inkonsistente resultater: Alvorligt.** Den statistiske heterogenicitet er høj. **Manglende overførbarhed: Ingen betydelig.** Ikke alle patienter havde ICS inden randomisering, men størstedelen havde og der ser ikke ud til at være en stor forskel mellem de studier hvor alle fik ICS inden intervention og dem hvor de fleste fik ICS inden intervention.. **Upræcist effektestimat: Ingen betydelig. Publikationsbias: Ingen betydelig.**

14. Systematisk oversigtsartikel [34] med inkluderede studier: [28], [30]. **Baselinerisiko/komparator:** Kontrolarm i reference brugt til interventionen.

15. **Risiko for bias: Alvorligt.** Utilstrækkeligt skjult randomisering, Selektiv rapportering af outcome, risk of bias på grund af gennemgående indblanding i forsøgsdesign og rapportering af resultater fra medicinalindustrien.. **Inkonsistente resultater: Alvorligt.** Den statistiske heterogenicitet er høj. **Manglende overførbarhed: Ingen betydelig.** Ikke alle patienter havde ICS inden randomisering, men størstedelen havde.. **Upræcist effektestimat: Alvorligt.** Brede konfidensintervaller. **Publikationsbias: Ingen betydelig.**

16. Baseret på data fra 11 studier. Opfølgningstid: 12-52 uger.

17. **Risiko for bias: Alvorligt.** Utilstrækkeligt skjult randomisering, Selektiv rapportering af outcome, risk of bias på grund af gennemgående indblanding i forsøgsdesign og rapportering af resultater fra medicinalindustrien.. **Inkonsistente resultater: Ingen betydelig. Manglende overførbarhed: Ingen betydelig.** Ikke alle patienter havde ICS inden randomisering, men størstedelen havde og der ser ikke ud til at være en stor forskel mellem de studier hvor alle fik ICS inden intervention og dem hvor de fleste fik ICS inden intervention.. **Upræcist effektestimat: Ingen betydelig. Publikationsbias: Ingen betydelig.**

Referencer

16. Bafadhel M., Peterson S., De Blas MA, Calverley PM, Rennard SI, Richter K., et al. : Predictors of exacerbation risk and response to budesonide in patients with chronic obstructive pulmonary disease: a post-hoc analysis of three randomised trials. The Lancet.Respiratory medicine 2018;6(2):117-126 Journal

24. Chapman KR, Hurst JR, Frent S-M, Larbig M, Fogel R, Guerin T, et al. : Long-Term Triple Therapy De-escalation to Indacaterol/Glycopyrronium in Patients with Chronic Obstructive Pulmonary Disease (SUNSET): A Randomized, Double-Blind, Triple-Dummy Clinical Trial. American Journal of Respiratory & Critical Care Medicine 2018;198(3):329-339

25. Ferguson GT, Rabe KF, Martinez FJ, Fabbri LM, Wang C., Ichinose M., et al. : Triple therapy with budesonide/glycopyrrolate/formoterol fumarate with co-suspension delivery technology versus dual therapies in chronic obstructive pulmonary disease (KRONOS): a double-blind, parallel-group, multicentre, phase 3 randomised controlled trial. The Lancet.Respiratory medicine 2018;6(10):747-758 Journal

26. Papi A., Vestbo J., Fabbri L., Corradi M., Prunier H., Cohuet G., et al. : Extrafine inhaled triple therapy versus dual bronchodilator therapy in chronic obstructive pulmonary disease (TRIBUTE): a double-blind, parallel group, randomised controlled trial. Lancet (London, England) 2018;391(10125):1076-1084 Journal

27. Pascoe S., Locantore N., Dransfield MT, Barnes NC, Pavord ID : Blood eosinophil counts, exacerbations, and response to the addition of inhaled fluticasone furoate to vilanterol in patients with chronic obstructive pulmonary disease: a secondary analysis of data from two parallel randomised controlled trials. The Lancet.Respiratory medicine 2015;3(6):435-442 Journal

28. Siddiqui SH, Guasconi A., Vestbo J., Jones P., Agusti A., Paggiaro P., et al. : Blood Eosinophils: A Biomarker of Response to Extrafine Beclomethasone/Formoterol in Chronic Obstructive Pulmonary Disease. American journal of respiratory and critical care medicine 2015;192(4):523-525 Journal

29. Watz H., Tetzlaff K., Wouters EF, Kirsten A., Magnussen H., Rodriguez-Roisin R., et al. : Blood eosinophil count and exacerbations in severe chronic obstructive pulmonary disease after withdrawal of inhaled corticosteroids: a post-hoc analysis of the WISDOM trial. The Lancet.Respiratory medicine 2016;4(5):390-398 Journal

30. Pascoe S, Barnes N, Brusselle G, Compton C, Criner GJ, Dransfield MT, et al. : Blood eosinophils and treatment response with triple and dual combination therapy in chronic obstructive pulmonary disease: analysis of the IMPACT trial. The Lancet. Respiratory medicine 2019;7(9):745-756 Pubmed Journal

31. Hanania NA, Papi A, Anzueto A, Martinez FJ, Rossman KA, Cappelletti CS, et al. : Efficacy and safety of two doses of budesonide/formoterol fumarate metered dose inhaler in COPD. Erj Open Research 2020;6(2):

32. Goyal P., Rossi A., Bader G., Pablo A. : Comparison of effect of indacaterol with salmeterol/fluticasone fixed dose combination on COPD exacerbations based on baseline blood eosinophil counts: post-hoc analysis from the instead study. Respirology (Carlton, Vic.) 2015;20 43 Journal Link

33. Rabe KF, Martinez FJ, Ferguson GT, Wang C., Singh D., Wedzicha JA, et al. : Triple Inhaled Therapy at Two Glucocorticoid Doses in Moderate-to-Very-Severe COPD. The New England journal of medicine 2020;383(1):35-48 Journal

34. NKR Pulje ICS til KOL patienter PICO 1-3 (EOS-grupper).

35. Metaanalyse for PICO 1-3. Link

3.2 - Patienter med KOL i behandling med ICS og med EOS mellem 150 og 300 celler/µL

PICO 2

Bør patienter med kronisk obstruktiv lungesygdom (KOL) og blod-eosinofilocytter (EOS) mellem 150 og 300 celler/µL fortsat behandles med inhalationssteroid (ICS)?

Svag anbefaling

Overvej at fortsætte behandling med inhalationssteroid (ICS) til patienter med kronisk obstruktiv lungesygdom (KOL) og blod-eosinofilocytter (EOS) mellem 150-300 celler/µL.

Patienter med KOL og EOS mellem 150-300 celler/µL forventes at have nogen effekt af fortsat behandling med ICS. Dette er særlig udtalt hos patienter med hyppige moderate til svære exacerbationer og/eller lav lungefunktion, da der er vist effekt på disse outcomes.

Studierne viser en reduktion af risiko for moderat til svær exacerbation på 20 % (konfidensinterval 6-31 %). Hvis patienten fx har én moderat til svær exacerbation om året vil dette svare til to exacerbationer mindre i løbet af 10 år. Patienten vil stadig rammes af de 8 andre exacerbationer.

Studierne viser en forbedring i lungefunktion (FEV1) på 50 mL (konfidensinterval 30-70 mL). Denne forbedring vil umiddelbart betyde mest for patienter med svært nedsat lungefunktion.

Hvis det ønskes at forsøge seponering, anbefaler arbejdsgruppen på baggrund af klinisk erfaring at seponere ICS gradvist i samarbejde med patienten. Fx reducér til halv dosis og fortsæt i 6-8 uger efterfulgt af seponering. Kan patienten ikke undvære ICS, anbefaler arbejdsgruppen, at behandlingen genoptages i lavest mulige effektive dosis. Se i øvrigt DLS' KOL vejledning.

Nøgleinformationer

Gavnlige og skadelige virkninger Lille netto gevinst eller små forskelle mellem alternativerne

Fortsat behandling med ICS nedsætter sandsynligvis risiko for *moderat til svær exacerbation* (rate ratio: 0,80 (95 % CI: 0,69 - 0,94)). Der er usikkert om resultatet er klinisk relevant. Arbejdsgruppen har forud for litteraturgennemgangen sat mindste kliniske relevante forskel til 25 %. Tiltroen til estimatet er moderat pga. risiko for bias.

Fortsat behandling med ICS nedsætter muligvis *frafald af alle årsager* i studierne (risk ratio: 0,78 (95 % CI: 0,73 - 0,84)). Tiltroen til estimatet er lav pga. risiko for bias og manglende overførbarhed. Ingen studier opgjorde *frafald af alle årsager* ud fra EOS og estimatet gælder for alle deltagere i studierne uanset EOS.

Fortsat behandling med ICS påvirker sandsynligvis ikke *antal patienter, der får alvorlige bivirkninger* (risk ratio: 0,96 (95 % CI: 0,9 - 1,03)). Tiltroen til estimatet er moderat pga. risiko for bias.

Fortsat behandling med ICS medfører sandsynligvis forbedring af *lungefunktionen* (FEV1 mean difference: 0,05 L (95 % CI: 0,03 - 0,07 L)). Tiltroen til estimatet er moderat pga. risiko for bias. Der er ikke fundet studier, som opgjorde *antal patienter med en væsentlig forbedring i lungefunktion* og det er arbejdsgruppens vurdering at fortsat behandling med ICS øger *antal patienter med en væsentlig forbedring i lungefunktion*.

Fortsat behandling med ICS øger sandsynligvis risiko for *pneumoni* (risk ratio: 1,39 (95 % CI: 1,19 - 1,63)). Tiltroen til estimatet er moderat pga. risiko for bias.

Fortsat behandling med ICS forbedrer muligvis *livskvalitet* (SGRQ-skala mean difference: 1,80 (95 % CI: 3,01 - 0,58)). Resultatet er ikke klinisk relevant, da mindste klinisk relevante forskel er sat til 4 *[70]*. Tiltroen til estimatet er lav pga. risiko for bias og upræcise effektestimater.

Vi er usikre på om fortsat behandling med ICS forbedre *dyspnø* (TDI-skala mean difference: 0,13 (95 % CI: -0,29 - 0,55)). Resultatet er ikke klinisk relevant, da mindste klinisk relevante forskel er sat til 1 *[71]*. Tiltroen til estimatet er meget lav pga. risiko for bias, og upræcist effektestimat.

Kvaliteten af evidensen

Lav

Der er moderat tiltro til evidensen for det kritiske outcome *moderate og svære exacerbationer*. Tiltroen til evidensen er nedgraderet pga. alvorlig risiko for bias som følge af utilstrækkeligt skjult randomisering, selektiv rapportering af outcome (næsten alle studier har subgruppeanalyseret på EOS som en post-hoc analyse), samt gennemgående indblanding fra medicinalindustrien i forsøgsdesign og rapportering af resultater.

Der er ligeledes moderat tiltro til evidensen for det kritiske outcome *alvorlige bivirkninger*. Tiltroen til evidensen er nedgraderet fra høj til moderat pga. alvorlig risiko for bias som følge af utilstrækkeligt skjult randomisering samt gennemgående indblanding fra medicinalindustrien i forsøgsdesign og rapportering af resultater. Alvorlige bivirkninger er ikke opgjort i EOS subgrupper, men her vurderes det, at EOS ikke har indflydelse.

Der er lav tiltro til evidensen for det kritiske outcome *frafald af alle årsager*. Tiltroen til evidensen er nedgraderet pga. alvorlig risiko for bias og alvorlig manglende overførbarhed. Risiko for bias skyldes utilstrækkeligt skjult randomisering, samt gennemgående indblanding fra medicinalindustrien i forsøgsdesign og rapportering af resultater. *Frafald af alle årsager* er ikke opgjort i EOS subgrupper, hvilket resulterer i manglende overførbarhed mellem den undersøgte population og målpopulationen for PICO. Særligt ved frafald vurderes det, at EOS har indflydelse. Frafald pga. manglende effekt, vil forventes at være større ved høj EOS og lavere ved lav EOS.

For de vigtige outcomes er kvaliteten moderat til meget lav (se evidensprofilen for information på de enkelte outcomes). Tiltroen til evidensen er nedgraderet pga. alvorlig risiko for bias, alvorlige inkonsistente resultater med uforklarlig varians, få studier og brede konfidensintervaller. Risiko for bias skyldes utilstrækkeligt skjult randomisering, selektiv rapportering af outcome (næsten alle studier har subgruppeanalyseret på EOS som en post-hoc analyse), samt risiko for bias pga. gennemgående indblanding i forsøgsdesign og rapportering af resultater fra medicinalindustrien.

Samlet set er kvaliteten af evidensen lav.

Patientpræferencer

Betydelig variation er forventet eller usikker

Det er arbejdsgruppens vurdering, at visse patienter ud fra en samlet vurdering af fordele og ulemper i samråd med læge vil være interesserede i at ophøre med ICS behandling, mens andre vil ønske at fortsætte behandlingen.

Rationale

Der blev i formuleringen af anbefalingen lagt vægt på at fortsat behandling med ICS har en effekt på risiko for moderat til svær exacerbation og lungefunktion og at der ikke er flere patienter med alvorlige bivirkninger ved fortsat behandling med ICS. Dette sammenholdes med at seponering af ICS ikke forværre dyspnø eller livskvalitet samt at seponering af ICS mindsker risiko for pneumoni. Der forventes betydelig variation i patientpræferencer.

Fokuseret Spørgsmål

Population: Patienter på 40 år eller derover med KOL, som har EOS mellem 150 og 300 celler/µL, ikke er nuværende astmapatienter og som har været i behandling med ICS mindst 3 måneder inden og/eller under forsøget.

Intervention: Behandling med ICS dagligt i mindst 3 måneder (gælder både nyopstartet eller fortsat behandling med ICS) – enten som monoterapi eller i tilgift til behovsmedicin eller anden inhalationsbehandling (ikke ICS).

Sammenligning: Ingen ICS-behandling (men kan have fået ICS-behandlingen forud for forsøgsopstart). Patienter kan være i behandling med behovsmedicin eller få anden inhalationsbehandling (ikke ICS).

Sammenfatning

Se sammenfatningen under PICO 1

**Outcome** Tidsramme	**Resultater og målinger**	**Effektestimater** Seponering	ICS	**Tiltro til estimaterne** (at de afspejler den sande effekt i populationen)	**Sammendrag**
**Alvorlige bivirkninger, antal patienter, alle EOS (serious adverse events, number of patients, all EOS)** Længst mulige follow-up (min. 3 mdr. fra intervention start)<br>9 Kritisk	Relative risiko 0.96 (CI 95% 0.9 - 1.03) Baseret på data fra 29,654 patienter i 11 studier. [1] Opfølgningstid: 12-52 uger.	**178** per 1.000	**171** per 1.000<br>Forskel: **7 færre** per 1.000 (CI 95% 18 færre - 5 flere)	**Moderat** på grund af alvorlig risiko for bias [2]	Fortsat behandling med ICS påvirker sandsynligvis ikke antallet af patienter som oplever alvorlige bivirkninger
**Frafald af alle årsager, alle EOS (All-cause drop-out, all EOS)** Længst mulige follow-up (min. 3 mdr. fra intervention start)<br>9 Kritisk	Relative risiko 0.78 (CI 95% 0.73 - 0.84) Baseret på data fra 29,723 patienter i 11 studier. [3] (Randomiserede studier) Opfølgningstid: 12-52 uger.	**233** per 1.000	**182** per 1.000<br>Forskel: **51 færre** per 1.000 (CI 95% 63 færre - 37 færre)	**Lav** på grund af alvorlig risiko for bias, på grund af alvorlig manglende overførbarhed [4]	Fortsat behandling med ICS nedsætter muligvis frafald af alle årsager
**Antal patienter med en væsentlig forbedring i lungefunktion (FEV1) (number of patients with improvement in lung function)**<br>6 Vigtig	Baseret på data fra patienter i 0 studier.				Vi fandt ingen studier der opgjorde antal patienter med forbedring i lungefunktion. Det er arbejdsgruppens kliniske vurdering at fortsat behandling med ICS sandsynligvis øger antallet af patienter med en væsentlig forbedring i lungefunktion.

**Outcome** Tidsramme	**Resultater og målinger**	**Effektestimater** Seponering	ICS	**Tiltro til estimaterne** (at de afspejler den sande effekt i populationen)	**Sammendrag**
**Pneumoni, antal patienter, alle EOS (Pneumonia, number of patients, all EOS)** Længst mulige follow-up (min. 3 mdr. fra intervention start)<br>6 Vigtig	Relative risiko 1.39 (CI 95% 1.19 - 1.63) Baseret på data fra 29,654 patienter i 11 studier. [5] (Randomiserede studier) Opfølgningstid: 12-52 uger.	32 per 1.000	44 per 1.000	**Moderat** på grund af alvorlig risiko for bias [6]	Fortsat behandling med ICS øger sandsynligvis risikoen for pneumoni
		Forskel: **12 flere** per 1.000 (CI 95% 6 flere - 20 flere)			
**Pneumoni rate (pneumonia rate)** Længst mulige follow-up (min. 3 mdr. fra intervention start)<br>6 Vigtig	Lavere bedre Baseret på data fra: 2,718 patienter i 2 studier. [7] (Randomiserede studier) Opfølgningstid: 48-52 uger.	Forskel: **MD 0.07 højere** (CI 95% 0.04 højere - 0.1 højere)		**Moderat** på grund af alvorlig risiko for bias [8]	Fortsat behandling med ICS øger sandsynligvis risikoen for pneumoni
**Lungefunktion (lung function)** Længst mulige follow-up (min. 3 mdr. fra intervention start)<br>6 Vigtig	Målt med: FEV1 Højere bedre Baseret på data fra: 3,116 patienter i 3 studier. [9] (Randomiserede studier) Opfølgningstid: 26-52 uger.	0.03 (gennemsnit)	0.08 (gennemsnit)	**Moderat** på grund af alvorlig risiko for bias [10]	Fortsat behandling med ICS medfører sandsynligvis forbedring af lungefunktion
		Forskel: **MD 0.05 højere** (CI 95% 0.03 højere - 0.07 højere)			
**Dyspnø (Dyspnea)** Længst mulige follow-up (min. 3 mdr. fra intervention start)<br>6 Vigtig	Målt med: TDI Skala: -9-9 Højere bedre Baseret på data fra: 7,389 patienter i 1 studier. [11] Opfølgningstid: 52 uger.	Forskel: **MD 0.13 højere** (CI 95% 0.29 lavere - 0.55 højere)		**Meget lav** på grund af alvorlig risiko for bias, på grund af meget alvorlig upræcist effektestimat [12]	Vi er usikre på, om fortsat behandling med ICS forbedrer dyspnø
**Livskvalitet (quality of life)** Længst mulige follow-up (min. 3 mdr. fra intervention start)<br>6 Vigtig	Målt med: SGRQ Skala: 0-100 Lavere bedre Baseret på data fra: 2,718 patienter i 2 studier. [13] Opfølgningstid: 48-52 uger.	Forskel: **MD 1.8 lavere** (CI 95% 3.01 lavere - 0.58 lavere)		**Lav** på grund af alvorlig risiko for bias, på grund af alvorlig upræcist effektestimat [14]	Fortsat behandling med ICS medfører muligvis ingen betydelig forbedring i livskvalitet

**Outcome** Tidsramme	**Resultater og målinger**	**Effektestimater** Seponering ICS	**Tiltro til estimaterne** (at de afspejler den sande effekt i populationen)	**Sammendrag**
**Moderat til svær exacerbation (exacerbat ons)** 15<br>Længst mulige follow-up (min. 3 mdr. fra intervention start)<br>9 Kritisk	Baseret på data fra: patienter i 6 studier. Opfølgningstid: 26-52 uger.	Rate ratio: 0.80 (CI 95%: 0.69 - 0.94)	**Moderat** på grund af alvorlig risiko for bias [16]	Fortsat behandling med ICS nedsætter sandsynligvis risikoen for exacerbationer Intervention

1. Systematisk oversigtsartikel [36] med inkluderede studier: [25], [33], [30], [31], [24], [29], [32], [27], [26], [16], [28]. **Baselinerisiko/komparator:** Kontrolarm i reference brugt til interventionen.
2. **Risiko for bias: Alvorligt.** Utilstrækkeligt skjult randomisering, risk of bias på grund af gennemgående indblanding i forsøgsdesign og rapportering af resultater fra medicinalindustrien., Utilstrækkeligt skjult randomisering. **Inkonsistente resultater: Ingen betydelig. Manglende overførbarhed: Ingen betydelig.** Ikke alle patienter havde ICS inden randomisering, men størstedelen havde og der ser ikke ud til at være en stor forskel mellem de studier hvor alle fik ICS inden intervention og dem hvor de fleste fik ICS inden intervention. Dette outcome er ikke opgjort på baggrund af blodeosinofiltallet, men for hele populationen i interventions- og comparisongruppen.. **Upræcist effektestimat: Ingen betydelig. Publikationsbias: Ingen betydelig.**
3. Systematisk oversigtsartikel [36] med inkluderede studier: [29], [28], [27], [24], [26], [25], [16], [33], [32], [31], [30]. **Baselinerisiko/komparator:** Kontrolarm i reference brugt til interventionen.
4. **Risiko for bias: Alvorligt.** Utilstrækkeligt skjult randomisering, risk of bias på grund af gennemgående indblanding i forsøgsdesign og rapportering af resultater fra medicinalindustrien.. **Inkonsistente resultater: Ingen betydelig.** Den statistiske heterogenicitet er høj (56%), men da de alle favoriserer ICS er der ikke trukket ned.. **Manglende overførbarhed: Alvorligt.** Forskelle mellem målpopulation og studiepopulation, da det ikke er subgruppeanalyseret på EOS. **Upræcist effektestimat: Ingen betydelig. Publikationsbias: Ingen betydelig.**
5. Systematisk oversigtsartikel [36] med inkluderede studier: [29], [26], [30], [16], [32], [25], [24], [28], [27], [31], [33]. **Baselinerisiko/komparator:** Kontrolarm i reference brugt til interventionen.
6. **Risiko for bias: Alvorligt.** Utilstrækkeligt skjult randomisering, risk of bias på grund af gennemgående indblanding i forsøgsdesign og rapportering af resultater fra medicinalindustrien.. **Inkonsistente resultater: Ingen betydelig. Manglende overførbarhed: Ingen betydelig.** Ikke alle patienter havde ICS inden randomisering, men størstedelen havde og der ser ikke ud til at være en stor forskel mellem de studier hvor alle fik ICS inden intervention og dem hvor de fleste fik ICS inden intervention. Dette outcome er ikke opgjort på baggrund af blodeosinofiltallet, men for hele populationen i interventions- og comparisongruppen. 2 studier opgjorde det for patienter med et blodeosinofiltal under 150 og her var samme tendens om end tydeligere. Studierne er lavet på forskellige typer af ICS, som muligvis har forskellig risiko for at give pneumoni.. **Upræcist effektestimat: Ingen betydelig. Publikationsbias: Ingen betydelig.**
7. Systematisk oversigtsartikel [36] med inkluderede studier: [30], [28]. **Baselinerisiko/komparator:** Kontrolarm i reference brugt til interventionen.

8. **Risiko for bias: Alvorligt.** Utilstrækkeligt skjult randomisering, Selektiv rapportering af outcome, risk of bias på grund af gennemgående indblanding i forsøgsdesign og rapportering af resultater fra medicinalindustrien.. **Inkonsistente resultater: Ingen betydelig. Manglende overførbarhed: Ingen betydelig. Upræcist effektestimat: Ingen betydelig. Publikationsbias: Ingen betydelig.**
9. Systematisk oversigtsartikel [36] med inkluderede studier: [30], [28], [24]. **Baselinerisiko/komparator:** Kontrolarm i reference brugt til interventionen.
10. **Risiko for bias: Alvorligt.** Utilstrækkeligt skjult randomisering, Selektiv rapportering af outcome, risk of bias på grund af gennemgående indblanding i forsøgsdesign og rapportering af resultater fra medicinalindustrien.. **Inkonsistente resultater: Ingen betydelig. Manglende overførbarhed: Ingen betydelig. Upræcist effektestimat: Ingen betydelig. Publikationsbias: Ingen betydelig.**
11. Systematisk oversigtsartikel [36] med inkluderede studier: [30]. **Baselinerisiko/komparator:** Kontrolarm i reference brugt til interventionen.
12. **Risiko for bias: Alvorligt.** Utilstrækkeligt skjult randomisering, Selektiv rapportering af outcome, risk of bias på grund af gennemgående indblanding i forsøgsdesign og rapportering af resultater fra medicinalindustrien.. **Inkonsistente resultater: Ingen betydelig. Manglende overførbarhed: Ingen betydelig. Upræcist effektestimat: Meget alvorligt.** Kun data fra ét studie, Brede konfidensintervaller. **Publikationsbias: Ingen betydelig.**
13. Systematisk oversigtsartikel [36] med inkluderede studier: [28], [30]. **Baselinerisiko/komparator:** Kontrolarm i reference brugt til interventionen.
14. **Risiko for bias: Alvorligt.** Utilstrækkeligt skjult randomisering, Selektiv rapportering af outcome, risk of bias på grund af gennemgående indblanding i forsøgsdesign og rapportering af resultater fra medicinalindustrien.. **Inkonsistente resultater: Ingen betydelig. Manglende overførbarhed: Ingen betydelig. Upræcist effektestimat: Alvorligt.** Brede konfidensintervaller. **Publikationsbias: Ingen betydelig.**
15. Baseret på data fra 6 studier. Opfølgningstid: 26-52 uger.
16. **Risiko for bias: Alvorligt.** Utilstrækkeligt skjult randomisering, Selektiv rapportering af outcome, risk of bias på grund af gennemgående indblanding i forsøgsdesign og rapportering af resultater fra medicinalindustrien.. **Inkonsistente resultater: Ingen betydelig.** Den statistiske heterogenicitet er høj, men da de alle favoriserer ICS er der ikke trukket ned.. **Manglende overførbarhed: Ingen betydelig. Upræcist effektestimat: Ingen betydelig. Publikationsbias: Ingen betydelig.**

Referencer

2. Price D., Yawn B., Brusselle G., Rossi A. : Risk-to-benefit ratio of inhaled corticosteroids in patients with COPD. Primary care respiratory journal : journal of the General Practice Airways Group 2013;22(1):92-100 Journal

16. Bafadhel M., Peterson S., De Blas MA, Calverley PM, Rennard SI, Richter K., et al. : Predictors of exacerbation risk and response to budesonide in patients with chronic obstructive pulmonary disease: a post-hoc analysis of three randomised trials. The Lancet.Respiratory medicine 2018;6(2):117-126 Journal

24. Chapman KR, Hurst JR, Frent S-M, Larbig M, Fogel R, Guerin T, et al. : Long-Term Triple Therapy De-escalation to Indacaterol/Glycopyrronium in Patients with Chronic Obstructive Pulmonary Disease (SUNSET): A Randomized, Double-Blind, Triple-Dummy Clinical Trial. American Journal of Respiratory & Critical Care Medicine 2018;198(3):329-339

25. Ferguson GT, Rabe KF, Martinez FJ, Fabbri LM, Wang C., Ichinose M., et al. : Triple therapy with budesonide/ glycopyrrolate/formoterol fumarate with co-suspension delivery technology versus dual therapies in chronic obstructive pulmonary disease (KRONOS): a double-blind, parallel-group, multicentre, phase 3 randomised controlled trial. The Lancet.Respiratory medicine 2018;6(10):747-758 Journal

26. Papi A., Vestbo J., Fabbri L., Corradi M., Prunier H., Cohuet G., et al. : Extrafine inhaled triple therapy versus dual bronchodilator therapy in chronic obstructive pulmonary disease (TRIBUTE): a double-blind, parallel group, randomised controlled trial. Lancet (London, England) 2018;391(10125):1076-1084 Journal

27. Pascoe S., Locantore N., Dransfield MT, Barnes NC, Pavord ID : Blood eosinophil counts, exacerbations, and response to the addition of inhaled fluticasone furoate to vilanterol in patients with chronic obstructive pulmonary disease: a secondary analysis of data from two parallel randomised controlled trials. The Lancet.Respiratory medicine 2015;3(6):435-442 Journal

28. Siddiqui SH, Guasconi A., Vestbo J., Jones P., Agusti A., Paggiaro P., et al. : Blood Eosinophils: A Biomarker of Response to Extrafine Beclomethasone/Formoterol in Chronic Obstructive Pulmonary Disease. American journal of respiratory and critical care medicine 2015;192(4):523-525 Journal

29. Watz H., Tetzlaff K., Wouters EF, Kirsten A., Magnussen H., Rodriguez-Roisin R., et al. : Blood eosinophil count and exacerbations in severe chronic obstructive pulmonary disease after withdrawal of inhaled corticosteroids: a post-hoc analysis of the WISDOM trial. The Lancet.Respiratory medicine 2016;4(5):390-398 Journal

30. Pascoe S, Barnes N, Brusselle G, Compton C, Criner GJ, Dransfield MT, et al. : Blood eosinophils and treatment response with triple and dual combination therapy in chronic obstructive pulmonary disease: analysis of the IMPACT trial. The Lancet. Respiratory medicine 2019;7(9):745-756 Pubmed Journal

31. Hanania NA, Papi A, Anzueto A, Martinez FJ, Rossman KA, Cappelletti CS, et al. : Efficacy and safety of two doses of budesonide/formoterol fumarate metered dose inhaler in COPD. Erj Open Research 2020;6(2):

32. Goyal P., Rossi A., Bader G., Pablo A. : Comparison of effect of indacaterol with salmeterol/fluticasone fixed dose combination on COPD exacerbations based on baseline blood eosinophil counts: post-hoc analysis from the instead study. Respirology (Carlton, Vic.) 2015;20 43 Journal Link

33. Rabe KF, Martinez FJ, Ferguson GT, Wang C., Singh D., Wedzicha JA, et al. : Triple Inhaled Therapy at Two Glucocorticoid Doses in Moderate-to-Very-Severe COPD. The New England journal of medicine 2020;383(1):35-48 Journal

36. NKR Pulje ICS til KOL patienter PICO 1-3 (EOS-grupper).

3.3 - Patienter med KOL i behandling med ICS og med EOS over 300 celler/µL

PICO 3

Bør patienter med kronisk obstruktiv lungesygdom (KOL) og blod-eosinofilocytter (EOS) over 300 celler/µL fortsat behandles med inhalationssteroid (ICS)?

Svag anbefaling

Overvej at fortsætte behandling med inhalationssteroid (ICS) til patienter med kronisk obstruktiv lungesygdom (KOL) og blod-eosinofilocytter (EOS) over 300 celler/µL.

Patienter med KOL og EOS over 300 celler/µL forventes at have effekt af fortsat behandling med ICS. Effekt er vist på forbedring af livskvalitet, lungefunktion og nedsat risiko for moderat til svær exacerbation.

Studierne viser en reduktion af risiko for moderat til svær exacerbation på 43 % (konfidensinterval 34-51 %). Hvis patienten fx har en moderat til svær exacerbation om året vil dette svare til 4 exacerbation mindre i løbet af 10 år. Patienten vil stadig rammes af de 6 andre exacerbationer.

Studierne viser en forbedring i lungefunktion (FEV1) på 60 mL (konfidensinterval 40-90 mL). Denne forbedring vil forventeligt betyde mere for patienter med svært nedsat lungefunktion, og mindre for patienter med let nedsat lungefunktion.

Studierne viser en klinisk relevant forbedring i livskvalitet på ca. 4 point (konfidensinterval 1-7 point) på SGRQ-skalaen fra 0-100.

Arbejdsgruppen anbefaler på baggrund af klinisk erfaring at reducere dosis af ICS til mindst mulige effektive dosis. Fx reducér og fortsæt med halv dosis. Se i øvrigt DLS' KOL vejledning.

Nøgleinformationer

Gavnlige og skadelige virkninger

Overvejende fordele ved det anbefalede alternativ

Fortsat behandling med ICS nedsætter sandsynligvis risiko for *moderat til svær exacerbation* (rate ratio: 0,57 (95 % CI: 0,49 - 0,66)). Resultatet er klinisk relevant, idet arbejdsgruppen forud for litteraturgennemgangen har sat mindste kliniske relevante forskel til at være 25 %. Tiltroen til estimatet er moderat pga. risiko for bias.

Fortsat behandling med ICS nedsætter muligvis *frafald af alle årsager* i studierne (risk ratio: 0,78 (95 % CI: 0,73 - 0,84)). Tiltroen til estimatet er lav pga. risiko for bias og manglende overførbarhed. Ingen studier opgjorde *frafald af alle årsager* ud fra EOS og estimatet gælder for alle deltagere i studierne uanset EOS.

Fortsat behandling med ICS påvirker sandsynligvis ikke *antal patienter, der får alvorlige bivirkninger* (risk ratio: 0,96 (95 % CI: 0,9 - 1,03)). Tiltroen til estimatet er moderat pga. risiko for bias.

Fortsat behandling med ICS medfører sandsynligvis forbedring af *lungefunktion* (FEV1 mean difference: 0,06 L (95 % CI: 0,02 - 0,08 L)). Tiltro til estimatet er moderat pga. risiko for bias. Der er ikke fundet studier, som opgjorde *antal patienter med en væsentlig forbedring i lungefunktion* og det er arbejdsgruppens vurdering at fortsat behandling med ICS øger *antal patienter med en væsentlig forbedring i lungefunktion*.

Fortsat behandling med ICS medfører muligvis forbedring af *livskvalitet* (SGRQ-skala mean difference: 3,98 (95 % CI: 6,96 - 1,01)). Resultatet er klinisk relevant, idet den mindste kliniske relevante forskel er sat til 4 *[70]*. Tiltroen til estimatet er lav pga. risiko for bias og manglende overførbarhed.

Fortsat behandling med ICS øger sandsynligvis risiko for *pneumoni* (risk ratio: 1,39 (95 % CI: 1,19 - 1,63)). Tiltroen til estimatet er moderat pga. risiko for bias.

Vi er usikre på, om fortsat behandling med ICS mindsker *dyspnø* (TDI-skala mean difference: 0,30 (95 % CI: -0,37 - 0,97). Resultatet er ikke klinisk relevant, iden den mindste kliniske relevante forskel er sat til 1 *[71]*. Tiltroen til estimatet er meget lav pga. risiko for bias og upræcist effektestimat.

Kvaliteten af evidensen Lav

Der er moderat tiltro til evidensen for det kritiske outcome *moderate til svære exacerbationer*. Tiltroen til evidensen er nedgraderet pga. alvorlig risiko for bias som følge af utilstrækkeligt skjult randomisering, selektiv rapportering af outcome (næsten alle studier har subgruppeanalyseret på EOS som en post-hoc analyse), samt gennemgående indblanding fra medicinalindustrien i forsøgsdesign og rapportering af resultater.

Der er ligeledes moderat tiltro til evidensen for det kritiske outcome *alvorlige bivirkninger*. Tiltroen til evidensen er trukket ned fra høj til moderat pga. alvorlig risiko for bias som følge af utilstrækkeligt skjult randomisering samt gennemgående indblanding fra medicinalindustrien i forsøgsdesign og rapportering af resultater. Alvorlige bivirkninger er ikke opgjort i EOS subgrupper, men her vurderes det, at EOS ikke har indflydelse.

Der er lav tiltro til evidensen for det kritiske outcome *frafald af alle årsager*. Tiltroen til evidensen er nedgraderet pga. alvorlig risiko for bias og alvorlig manglende overførbarhed. Risiko for bias skyldes utilstrækkeligt skjult randomisering, samt gennemgående indblanding fra medicinalindustrien i forsøgsdesign og rapportering af resultater. Frafald af alle årsager er ikke opgjort i EOS subgrupper, hvilket resulterer i manglende overførbarhed mellem den undersøgte population og målpopulationen for PICO. Særligt ved frafald vurderedes det, at EOS har indflydelse. Frafald pga. manglende effekt, vil forventes at være større ved høj EOS og lavere ved lav EOS.

For de vigtige outcomes er kvaliteten moderat til meget lav (se evidensprofilen for information på de enkelte outcomes). Tiltroen til evidensen er nedgraderet pga. alvorlig risiko for bias, alvorlige inkonsistente resultater med uforklarlig varians, få studier og brede konfidensintervaller. Risiko for bias skyldes utilstrækkeligt skjult randomisering, selektiv rapportering af outcome (næsten alle studier har subgruppeanalyseret på EOS som en post-hoc analyse), samt risiko for bias pga. gennemgående indblanding i forsøgsdesign og rapportering af resultater fra medicinalindustrien.

Samlet set er kvaliteten af evidensen lav.

Patientpræferencer Ingen betydelig variation forventet

Det er arbejdsgruppens vurdering at de fleste patienter ud fra en samlet vurdering af fordele og ulemper i samråd med læge vil være interesserede i at fortsætte behandlingen med ICS.

Rationale

Der blev i formuleringen af anbefalingen lagt vægt på at fortsat behandling med ICS har en effekt på risiko for moderat til svær exacerbation, lungefunktion og livskvalitet. ICS påvirker sandsynligvis ikke antallet af patienter som oplever alvorlige bivirkninger i betydelig grad. Dette sammenholdes med, at seponering af ICS ikke forværrer dyspnø og at seponering af ICS mindsker risiko for pneumoni. Der forventes ingen betydelig variation i patientpræferencer.

Fokuseret Spørgsmål

Population: Patienter på 40 år eller derover med KOL, som har EOS over 300 celler/µL, ikke er nuværende astmapatienter og som har været i behandling med ICS mindst 3 måneder inden og/eller under forsøget.

Intervention: Behandling med ICS dagligt i mindst 3 måneder (gælder både nyopstartet eller fortsat behandling med ICS) – enten som monoterapi eller i tilgift til behovsmedicin eller anden inhalationsbehandling (ikke ICS).

Sammenligning: Ingen ICS-behandling (men kan have fået ICS-behandlingen forud for forsøgsopstart). Patienter kan være i behandling med behovsmedicin eller få anden inhalationsbehandling (ikke ICS).

Sammenfatning

Se sammenfatningen under PICO 1

**Outcome** Tidsramme	**Resultater og målinger**	**Effektestimater** Seponering ICS	**Tiltro til estimaterne** (at de afspejler den sande effekt i populationen)	**Sammendrag**
**Alvorlige bivirkninger, antal patienter, alle EOS (serious adverse events, number of patients, all EOS)** Længst mulige follow-up (min. 3 mdr. fra intervention start) 9 Kritisk	Relative risiko 0.96 (CI 95% 0.9 - 1.03) Baseret på data fra 29,654 patienter i 11 studier. [1] (Randomiserede studier) Opfølgningstid: 12-52 uger.	Seponering: 178 per 1.000; ICS: 171 per 1.000. Forskel: **7 færre** per 1.000 (CI 95% 18 færre - 5 flere)	**Moderat** på grund af alvorlig risiko for bias [2]	Fortsat behandling med ICS påvirker sandsynligvis ikke antallet af patienter som oplever alvorlige bivirkninger
**Frafald af alle årsager, alle EOS (All-cause drop-out, all EOS)** Længst mulige follow-up (min. 3 mdr. fra intervention start) 9 Kritisk	Relative risiko 0.78 (CI 95% 0.73 - 0.84) Baseret på data fra 29,723 patienter i 11 studier. [3] Opfølgningstid: 12-52 uger.	Seponering: 233 per 1.000; ICS: 182 per 1.000. Forskel: **51 færre** per 1.000 (CI 95% 63 færre - 37 færre)	**Lav** på grund af alvorlig risiko for bias, på grund af alvorlig manglende overførbarhed [4]	Fortsat behandling med ICS nedsætter muligvis frafald af alle årsager
**Antal patienter med en væsentlig forbedring i lungefunktion (FEV1) (number of patients with improvement in lung function)** Længst mulige follow-up (min. 3 mdr. fra intervention start) 6 Vigtig	Baseret på data fra patienter i 0 studier.			Vi fandt ingen studier der opgjorde antal patienter med forbedring i lungefunktion. Det er arbejdsgruppens kliniske vurdering at fortsat behandling med ICS sandsynligvis øger antallet af patienter med en væsentlig forbedring i lungefunktion
**Pneumoni, antal patienter, alle EOS (Pneumonia, number of patients, all EOS)** Længst mulige follow-up (min. 3 mdr. fra intervention start) 6 Vigtig	Relative risiko 1.39 (CI 95% 1.19 - 1.63) Baseret på data fra 29,654 patienter i 11 studier. [5] (Randomiserede studier) Opfølgningstid: 12-52 uger.	Seponering: 32 per 1.000; ICS: 44 per 1.000. Forskel: **12 flere** per 1.000 (CI 95% 6 flere - 20 flere)	**Moderat** på grund af alvorlig risiko for bias [6]	Fortsat behandling med ICS øger sandsynligvis risiko for pneumoni

**Outcome** Tidsramme	**Resultater og målinger**	**Effektestimater** Seponering ICS	**Tiltro til estimaterne** (at de afspejler den sande effekt i populationen)	**Sammendrag**
**Pneumoni rate (pneumonia rate)** Længst mulige follow-up (min. 3 mdr. fra intervention start)<br>6 Vigtig	Lavere bedre Baseret på data fra: 1,505 patienter i 2 studier. [7] (Randomiserede studier) Opfølgningstid: 48-52 uger.	Forskel: **MD 0.06 højere** (CI 95% 0.01 højere - 0.12 højere)	**Moderat** på grund af alvorlig risiko for bias [8]	Fortsat behandling med ICS øger sandsynligvis risikoen for pneumoni
**Lungefunktion (lung function)** Længst mulige follow-up (min. 3 mdr. fra intervention start)<br>6 Vigtig	Målt med: FEV1 Højere bedre Baseret på data fra: 2,150 patienter i 4 studier. [9] Opfølgningstid: 26-52 uger.	**0.02** (gennemsnit) **0.08** (gennemsnit)<br>Forskel: **MD 0.06 højere** (CI 95% 0.04 højere - 0.09 højere)	**Moderat** på grund af alvorlig risiko for bias [10]	Fortsat behandling med ICS medfører sandsynligvis forbedring af lungefunktion
**Dyspnø (Dyspnea)** Længst mulige follow-up (min. 3 mdr. fra intervention start)<br>6 Vigtig	Målt med: TDI Skala: -9-9 Højere bedre Baseret på data fra: 1,256 patienter i 1 studier. [11] Opfølgningstid: 52 uger.	Forskel: **MD 0.3 højere** (CI 95% 0.37 lavere - 0.97 højere)	**Meget lav** på grund af alvorlig risiko for bias, på grund af meget alvorlig upræcist effektestimat [12]	Vi er usikre på, om fortsat behandling med ICS mindsker dyspnø
**Livskvalitet (quality of life)** Længst mulige follow-up (min. 3 mdr. fra intervention start)<br>6 Vigtig	Målt med: SGRQ Skala: 0-100 Lavere bedre Baseret på data fra: 1,505 patienter i 2 studier. [13] (Randomiserede studier) Opfølgningstid: 48-52 uger.	Forskel: **MD 3.98 lavere** (CI 95% 6.96 lavere - 1.01 lavere)	**Lav** på grund af alvorlig risiko for bias, på grund af alvorlig upræcist effektestimat [14]	Fortsat behandling med ICS medfører muligvis forbedring af livskvalitet

**Outcome** Tidsramme	**Resultater og målinger**	**Effektestimater** Seponering ICS	**Tiltro til estimaterne** (at de afspejler den sande effekt i populationen)	**Sammendrag**
**Moderat til svær exacerbation (exacerbat ons)** 15<br>Længst mulige follow-up (min. 3 mdr. fra intervention start)<br>9 Kritisk	Baseret på data fra: patienter i 6 studier. Opfølgningstid: 26-52 uger.	Rate ratio: 0.57 (CI 95% 0.49 - 0.66)	**Moderat** på grund af alvorlig risiko for bias [16]	Fortsat behandling med ICS nedsætter sandsynligvis risikoen for exacerbationer væsentligt

1. Systematisk oversigtsartikel [37] med inkluderede studier: [31], [30], [16], [24], [25], [26], [27], [29], [28], [33], [32]. **Baselinerisiko/komparator:** Kontrolarm i reference brugt til interventionen.
2. **Risiko for bias: Alvorligt.** Utilstrækkeligt skjult randomisering, Selektiv rapportering af outcome, risk of bias på grund af gennemgående indblanding i forsøgsdesign og rapportering af resultater fra medicinalindustrien.. **Inkonsistente resultater: Ingen betydelig. Manglende overførbarhed: Ingen betydelig.** Ikke alle patienter havde ICS inden randomisering, men størstedelen havde og der ser ikke ud til at være en stor forskel mellem de studier hvor alle fik ICS inden intervention og dem hvor de fleste fik ICS inden intervention. Dette outcome er ikke opgjort på baggrund af blodeosinofiltallet, men for hele populationen i interventions- og comparisongruppen.. **Upræcist effektestimat: Ingen betydelig. Publikationsbias: Ingen betydelig.**
3. Systematisk oversigtsartikel [37] med inkluderede studier: [26], [25], [30], [29], [24], [16], [32], [28], [27], [31], [33]. **Baselinerisiko/komparator:** Kontrolarm i reference brugt til interventionen.
4. **Risiko for bias: Alvorligt.** Utilstrækkeligt skjult randomisering og risk of bias på grund af gennemgående indblanding i forsøgsdesign og rapportering af resultater fra medicinalindustrien.. **Inkonsistente resultater: Ingen betydelig.** Den statistiske heterogenicitet er høj (56%), men da de alle favoriserer ICS er der ikke trukket ned.. **Manglende overførbarhed: Alvorligt.** Forskelle mellem målpopulation og studiepopulation, da det ikke er subgruppeanalyseret på EOS. **Upræcist effektestimat: Ingen betydelig. Publikationsbias: Ingen betydelig.**
5. Systematisk oversigtsartikel [37] med inkluderede studier: [26], [31], [25], [16], [32], [27], [30], [33], [28], [24], [29]. **Baselinerisiko/komparator:** Kontrolarm i reference brugt til interventionen.
6. **Risiko for bias: Alvorligt.** Utilstrækkeligt skjult randomisering, risk of bias på grund af gennemgående indblanding i forsøgsdesign og rapportering af resultater fra medicinalindustrien.. **Inkonsistente resultater: Ingen betydelig.** Den statistiske heterogenicitet er høj. **Manglende overførbarhed: Ingen betydelig.** Ikke alle patienter havde ICS inden randomisering, men størstedelen havde og der ser ikke ud til at være en stor forskel mellem de studier hvor alle fik ICS inden intervention og dem hvor de fleste fik ICS inden intervention. Dette outcome er ikke opgjort på baggrund af blodeosinofiltallet, men for hele populationen i interventions- og comparisongruppen. 2 studier opgjorde det for patienter med et blodeosinofiltal under 150 og her var samme tendens om end tydeligere. Studierne er lavet på forskellige typer af ICS, som muligvis har forskellig risiko for at give pneumoni.. **Upræcist effektestimat: Ingen betydelig.** Brede konfidensintervaller. **Publikationsbias: Ingen betydelig.**
7. Systematisk oversigtsartikel [37] med inkluderede studier: [28], [30]. **Baselinerisiko/komparator:** Kontrolarm i reference brugt til interventionen.
8. **Risiko for bias: Alvorligt.** Utilstrækkeligt skjult randomisering, Selektiv rapportering af outcome, risk of bias på grund af gennemgående indblanding i forsøgsdesign og rapportering af resultater fra medicinalindustrien.. **Inkonsistente resultater: Ingen betydelig.** Den statistiske heterogenicitet er høj, men da de alle favoriserer seponering er der ikke trukket ned.. **Manglende overførbarhed: Ingen betydelig. Upræcist effektestimat: Ingen betydelig. Publikationsbias: Ingen betydelig.**

9. Systematisk oversigtsartikel [37] med inkluderede studier: [29], [30], [28], [24]. **Baselinerisiko/komparator:** Kontrolarm i reference brugt til interventionen.
10. **Risiko for bias: Alvorligt.** Utilstrækkeligt skjult randomisering, Selektiv rapportering af outcome, risk of bias på grund af gennemgående indblanding i forsøgsdesign og rapportering af resultater fra medicinalindustrien.. **Inkonsistente resultater: Ingen betydelig. Manglende overførbarhed: Ingen betydelig. Upræcist effektestimat: Ingen betydelig. Publikationsbias: Ingen betydelig.**
11. Systematisk oversigtsartikel [37] med inkluderede studier: [30]. **Baselinerisiko/komparator:** Kontrolarm i reference brugt til interventionen.
12. **Risiko for bias: Alvorligt.** Utilstrækkeligt skjult randomisering, Selektiv rapportering af outcome, risk of bias på grund af gennemgående indblanding i forsøgsdesign og rapportering af resultater fra medicinalindustrien.. **Inkonsistente resultater: Ingen betydelig. Manglende overførbarhed: Ingen betydelig. Upræcist effektestimat: Meget alvorligt.** Kun data fra ét studie, Brede konfidensintervaller. **Publikationsbias: Ingen betydelig.**
13. Systematisk oversigtsartikel [37] med inkluderede studier: [28], [30]. **Baselinerisiko/komparator:** Kontrolarm i reference brugt til interventionen.
14. **Risiko for bias: Alvorligt.** Utilstrækkeligt skjult randomisering, Selektiv rapportering af outcome, risk of bias på grund af gennemgående indblanding i forsøgsdesign og rapportering af resultater fra medicinalindustrien.. **Inkonsistente resultater: Ingen betydelig.** Den statistiske heterogenicitet er høj, men da de alle favoriserer seponering er der ikke trukket ned.. **Manglende overførbarhed: Ingen betydelig. Upræcist effektestimat: Alvorligt.** Brede konfidensintervaller. **Publikationsbias: Ingen betydelig.**
15. Baseret på data fra 6 studier. Opfølgningstid: 26-52 uger.
16. **Risiko for bias: Alvorligt.** Utilstrækkeligt skjult randomisering, Selektiv rapportering af outcome, risk of bias på grund af gennemgående indblanding i forsøgsdesign og rapportering af resultater fra medicinalindustrien.. **Inkonsistente resultater: Ingen betydelig. Manglende overførbarhed: Ingen betydelig.** Ikke alle patienter havde ICS inden randomisering, men størstedelen havde og der ser ikke ud til at være en stor forskel mellem de studier hvor alle fik ICS inden intervention og dem hvor de fleste fik ICS inden intervention. Dette outcome er ikke opgjort på baggrund af blodeosinofiltallet, men for hele populationen i interventions- og comparisongruppen.. **Upræcist effektestimat: Ingen betydelig. Publikationsbias: Ingen betydelig.**

Referencer

2. Price D., Yawn B., Brusselle G., Rossi A. : Risk-to-benefit ratio of inhaled corticosteroids in patients with COPD. Primary care respiratory journal : journal of the General Practice Airways Group 2013;22(1):92-100 Journal

16. Bafadhel M., Peterson S., De Blas MA, Calverley PM, Rennard SI, Richter K., et al. : Predictors of exacerbation risk and response to budesonide in patients with chronic obstructive pulmonary disease: a post-hoc analysis of three randomised trials. The Lancet.Respiratory medicine 2018;6(2):117-126 Journal

24. Chapman KR, Hurst JR, Frent S-M, Larbig M, Fogel R, Guerin T, et al. : Long-Term Triple Therapy De-escalation to Indacaterol/Glycopyrronium in Patients with Chronic Obstructive Pulmonary Disease (SUNSET): A Randomized, Double-Blind, Triple-Dummy Clinical Trial. American Journal of Respiratory & Critical Care Medicine 2018;198(3):329-339

25. Ferguson GT, Rabe KF, Martinez FJ, Fabbri LM, Wang C., Ichinose M., et al. : Triple therapy with budesonide/glycopyrrolate/formoterol fumarate with co-suspension delivery technology versus dual therapies in chronic obstructive pulmonary disease (KRONOS): a double-blind, parallel-group, multicentre, phase 3 randomised controlled trial. The Lancet.Respiratory medicine 2018;6(10):747-758 Journal

26. Papi A., Vestbo J., Fabbri L., Corradi M., Prunier H., Cohuet G., et al. : Extrafine inhaled triple therapy versus dual bronchodilator therapy in chronic obstructive pulmonary disease (TRIBUTE): a double-blind, parallel group, randomised controlled trial. Lancet (London, England) 2018;391(10125):1076-1084 Journal

27. Pascoe S., Locantore N., Dransfield MT, Barnes NC, Pavord ID : Blood eosinophil counts, exacerbations, and response to the addition of inhaled fluticasone furoate to vilanterol in patients with chronic obstructive pulmonary disease: a secondary analysis of data from two parallel randomised controlled trials. The Lancet.Respiratory medicine 2015;3(6):435-442 Journal

28. Siddiqui SH, Guasconi A., Vestbo J., Jones P., Agusti A., Paggiaro P., et al. : Blood Eosinophils: A Biomarker of Response to Extrafine Beclomethasone/Formoterol in Chronic Obstructive Pulmonary Disease. American journal of respiratory and critical care medicine 2015;192(4):523-525 Journal

29. Watz H., Tetzlaff K., Wouters EF, Kirsten A., Magnussen H., Rodriguez-Roisin R., et al. : Blood eosinophil count and exacerbations in severe chronic obstructive pulmonary disease after withdrawal of inhaled corticosteroids: a post-hoc analysis of the WISDOM trial. The Lancet.Respiratory medicine 2016;4(5):390-398 Journal

30. Pascoe S, Barnes N, Brusselle G, Compton C, Criner GJ, Dransfield MT, et al. : Blood eosinophils and treatment response with triple and dual combination therapy in chronic obstructive pulmonary disease: analysis of the IMPACT trial. The Lancet. Respiratory medicine 2019;7(9):745-756 Pubmed Journal

31. Hanania NA, Papi A, Anzueto A, Martinez FJ, Rossman KA, Cappelletti CS, et al. : Efficacy and safety of two doses of budesonide/formoterol fumarate metered dose inhaler in COPD. Erj Open Research 2020;6(2):

32. Goyal P., Rossi A., Bader G., Pablo A. : Comparison of effect of indacaterol with salmeterol/fluticasone fixed dose combination on COPD exacerbations based on baseline blood eosinophil counts: post-hoc analysis from the instead study. Respirology (Carlton, Vic.) 2015;20 43 Journal Link

33. Rabe KF, Martinez FJ, Ferguson GT, Wang C., Singh D., Wedzicha JA, et al. : Triple Inhaled Therapy at Two Glucocorticoid Doses in Moderate-to-Very-Severe COPD. The New England journal of medicine 2020;383(1):35-48 Journal

37. NKR Pulje ICS til KOL patienter PICO 1-3 (EOS-grupper).

4 - Patienter med KOL i behandling med ICS og uden moderat til svær exacerbation det seneste år

PICO 4
Bør patienter med kronisk obstruktiv lungesygdom (KOL) fortsat behandles med inhalationssteroid (ICS) efter mindst et år uden moderat til svær exacerbation?

Baggrund for valg af spørgsmålet
ICS kan reducere risiko for exacerbation hos patienter med KOL *[38][39]* og behandling med ICS anbefales derfor til patienter med hyppige exacerbationer *[1]*. Det er derfor nærliggende at undersøge effekten af fortsat behandling med ICS hos de patienter med KOL, som har lav risiko for exacerbation. Det er svært at forudse, hvilke patienter, der har en særlig lav risiko for at få fremtidige exacerbationer, og for nu er den bedste indikator for fremtidig exacerbationer, hvorvidt patienten har haft tidligere exacerbationer *[40]*. Patienter med KOL og uden hyppige exacerbationer formodes at have stabil KOL og en lavere risiko for fremtidige exacerbationer. Det vurderes derfor relevant at undersøge, om denne gruppe af patienter med stabil KOL fortsat vil have gavn af behandling med ICS. Arbejdsgruppen har valgt at definere patienter med stabil KOL, som patienter med KOL ud en moderat til svær exacerbation i mindst ét år, da dette må betragtes som tegn på stabil sygdom. Omkring halvdelen af patienter med KOL i Danmark har ikke haft en exacerbation det sidste år *[4]*.

Svag anbefaling

Overvej at fortsætte behandling med inhalations steorid (ICS) til patienter med kronisk obstruktiv lungesygdom (KOL) der har været uden moderat til svær exacerbation det sidste år.

Patienter med KOL uden moderat til svær exacerbation det seneste år forventes af have effekt af fortsat behandling med ICS. Effekten er vist ved en mulig nedsat risiko for moderat til svær exacerbation og mindre frafald i studierne.

Arbejdsgruppen anbefaler på baggrund af klinisk erfaring at reducere dosis af ICS til mindst mulige effektive dosis. Fx reducér og fortsæt med halv dosis. *Se i øvrigt DLS' KOL vejledning.*

Nøgleinformationer

Gavnlige og skadelige virkninger

Lille netto gevinst eller små forskelle mellem alternativerne

Fortsat behandling med ICS reducerer muligvis risiko for *moderat til svær exacerbation* (rate ratio: 0,67 (95 % CI: 0,41 - 1,10)). Resultatet er klinisk relevant, idet arbejdsgruppen forud for litteraturgennemgangen sat mindste kliniske relevante forskel til at være 25 %. Tiltroen til estimatet er lav pga. inkonsistente resultater og brede konfidensintervaller.

Fortsat behandling med ICS reducerer sandsynligvis *frafald af alle årsager* i studierne (risk ratio 0,83 (95 % CI: 0,68 - 1,02)). Tiltroen til estimatet er moderat pga. brede konfidensintervaller.

Fortsat behandling med ICS øger muligvis *antal patienter, der får alvorlige bivirkninger* (risk ratio: 1,63 (95 % CI: 0,44 - 6,05)). Tiltroen til estimatet er lav pga. inkonsistente resultater med uforklarlig varians og brede konfidensintervaller.

Fortsat behandling med ICS øger sandsynligvis risiko for *pneumoni* (risk ratio: 1,22 (95 % CI: 0,44 - 3,33)). Tiltroen til estimatet er moderat pga. brede konfidensintervaller.

Fortsat behandling med ICS påvirker sandsynligvis ikke *lungefunktion* (FEV1 mean difference: 0,01 L (95 % CI: -0,01 - 0,04)). Tiltroen til estimatet er moderat pga. brede konfidensintervaller. Der er ikke fundet studier, som opgjorde *antal patienter med en væsentlig forbedring i lungefunktion* og det er arbejdsgruppens vurdering at fortsat behandling med ICS har ingen eller ubetydelig påvirkning af dette outcome.

Fortsat behandling med ICS medfører ikke forbedring i livskvalitet (SGRQ-skala mean difference: 1,17 (95 % CI: 0,67 - 1,67)). Resultatet er ikke klinisk relevant, idet den mindste kliniske relevante forskel er sat til 4 *[70]*. Tiltroen til estimatet er høj.

Fortsat behandling med ICS påvirker muligvis ikke dyspnø (TDI-skala mean difference: 0,12 (95 % CI: -0,71 - 0,47)). Resultatet er ikke klinisk relevant, idet den mindst klinisk relevante forskel er sat til 1 *[71]*. Tiltroen til estimatet er lav pga. manglende overførbarhed og upræcist effektestimat.

Kvaliteten af evidensen

Lav

Der er lav tiltro til evidensen for det kritiske outcome *moderate og svære exacerbationer*. Tiltroen til evidensen er nedgraderet pga. brede konfidensintervaller og alvorlig inkonsistente resultater med uforklarlig varians (I^2: 77 %).

Der er ligeledes lav tiltro til evidensen for det kritiske outcome *antal patienter med alvorlige bivirkninger*. Tiltroen til evidensen er nedgraderet pga. brede konfidensintervaller, inkonsistente resultater med uforklarlig varians og at retningen af effekten er ikke konsistent imellem de inkluderede studier.

Der er moderat tiltro til evidensen for det kritiske outcome *frafald af alle årsager*. Tiltroen til evidensen er nedgraderet pga. brede konfidensintervaller.

For de vigtige outcomes er kvaliteten høj til lav pga. brede konfidensintervaller, forskelle mellem ønsket intervention/komparator og data fra ét studie.

Samlet set er kvaliteten af evidensen lav.

Patientpræferencer

Ingen betydelig variation forventet

Det er arbejdsgruppens vurdering at de fleste patienter ud fra en samlet vurdering af fordele og ulemper i samråd med læge vil være interesserede i at fortsætte behandlingen med ICS.

Rationale

Der blev i formuleringen af anbefalingen lagt vægt på at fortsat behandling med ICS har en begrænset effekt på risiko for moderat til svær exacerbation, og at der ikke er flere patienter med alvorlige bivirkninger ved fortsat behandling med ICS. Dette sammenholdes med, at seponering af ICS ikke forværrer dyspnø eller lungefunktionen. Der forventes ingen betydelig variation i patientpræferencer.

Fokuseret Spørgsmål

Population: Patienter på 40 år eller derover med KOL som ikke er nuværende astmapatienter, har været stabile (uden moderate eller alvorlige exacerbationer) i mindst et år og som har været i behandling med ICS mindst 3 måneder inden og/eller under forsøget.
Intervention: Behandling med ICS dagligt i mindst 3 måneder (gælder både nyopstartet eller fortsat behandling med ICS) – enten som monoterapi eller i tilgift til behovsmedicin eller anden inhalationsbehandling (ikke ICS).
Sammenligning: Ingen ICS-behandling (men kan have fået ICS-behandlingen forud for forsøgsopstart). Patienter kan være i behandling med behovsmedicin eller få anden inhalationsbehandling (ikke ICS).

Sammenfatning

Litteratur
Størstedelen af evidensgrundlaget for PICO 4 er et Cochrane review *[41]*, hvorfra der blev fundet 3 randomiserede forsøg *[42][43][44]*. Cochrane reviewet omhandlede kun ICS + langtidsvirkende β2-agonister (LABA) vs. LABA, hvorfor en supplerende søgning blev foretaget og yderligere 2 randomiserede forsøg *[45][24]* blev inkluderet. Evidensgrundlaget er således 5 randomiserede forsøg *[24][42][43][44][45]*.

For at undersøge robustheden af overstående studier, blev der foretaget en sensitivitetsanalyse på følgende outcomes: *risiko for moderat til svær exacerbation*, *pneumoni* og *frafald af alle årsager*. Til sensitivitetsanalyse blev der via litteraturgennemgang fundet 19 randomiserede forsøg *[16][24][25][26][28][29][30][31][33][54][56][57][59][47][62][64][65][67][68]*. Disse forsøg havde ikke analyseret på patienter uden exacerbation i et år, men overholdt i øvrigt vores inklusionskriterier.
Flow charts findes her.

Gennemgang af evidensen
De inkluderede 4 studier var randomiserede kontrollerede forsøg med i alt 3.982 patienter uden exacerbation det sidste år. Interventionerne bestod af ICS i kombinationer med LABA eller både LABA og LAMA. Varigheden af interventionerne varierede mellem 24 til 52 uger. Sammenligningsgrupper var enten LABA alene eller i kombination med LAMA. På tværs af studierne var patienterne primært mænd, med en aldersspredning på 59,6 til 66,8 år, BMI mellem 26,1 til 28,2 kg/m2, og lige under halvdelen var nuværende rygere. I 3 af studierne havde ingen mild KOL bedømt ud fra lungefunktionsnedsættelsen målt ved FEV1 (GOLD-klassificering) mens mellem 50 og 99 % havde moderat KOL, og de resterende havde svær eller meget svær KOL *[24][44][45]*. De sidste to studier *[42][43]* havde ikke opgiver sværhedsgraden af lungefunktionsnedsættelsen. Ét studie *[44]* havde som inklusionkriterie, at patienterne skulle være uden exacerbation i det foregående år. I to studier *[45][24]* havde størstedelen af patienterne (hhv. 74 % og 66 %) været uden exacerbation det sidste år. De to studier havde subgruppeanalyseret lungefunktionen hos de patienter som ikke havde exacerbationer året før de indgik i studiet. Det ene studie *[45]* havde yderligere subgruppeanalyseret risikoen for moderate og svære exacerbationer hos de patienter som ikke havde exacerbationer året før de indgik i studiet. To studier *[42][43]* havde ikke udgivet data, der var subgruppeanalyseret på patienter uden exacerbationer i året før, men Cochrane havde fået disse upublicerede data.

Pneumoni/exacerbation
Alle definitioner af exacerbation og pneumoni fra studierne er inkluderet.

Resultater
På det kritiske outcome *moderat til svær exacerbation*, var der en tendens til nedsat risiko ved fortsat behandling med ICS, både når exacarbationer var opgjort som rate ratio (0,67; 95 % CI: 0,41 - 1,10), som antal personer med moderate til svære exacarbationer (risk ratio: 0,90; 95 % CI: 0,45 - 1,80), eller som antal personer med svære exacarbationer (risk ratio: 0,73; 95 % CI: 0,27 - 1,95). Resultatet blev bekræftet i subgruppeanalysen, hvor vi sammenlignede resultatet med resultatet fra studier, hvor patienterne ikke var selekteret på baggrund af exacabationshistorik (rate ratio: 0,77; 95 % CI: 0,72 - 0,82; test for subgruppeforskelle p=0,59). Tiltroen til effektestimatet var lav, pga. alvorligt upræcist effektestimat, da der er uforklarlig variabilitet, og pga. alvorlige inkonsistente resultater, da der er brede konfidensintervaller.

Fortsat behandling med ICS øger muligvis *antallet af patienter der får alvorlige bivirkninger*, som ligeledes er et kritisk outcome (risk ratio: 1,63; 95 % CI: 0,44 - 6,05). Tiltroen til effektestimatet var lav, pga. alvorlig upræcist effektestimat, da der er uforklarlig variabilitet, og pga. alvorlig inkonsistente resultater, da der er brede konfidensintervaller.

På det kritiske outcome *frafald af alle årsager*, vil fortsat behandling med ICS sandsynligvis nedsætte risikoen (risk ratio: 0,83; 95 % CI: 0,68 - 1,02), hvilket også blev bekræftet i subgruppeanalysen hvor vi sammenlignede resultatet med resultatet fra studier, hvor patienterne ikke var selekteret på baggrund af exacabationshistorik (risk ratio: 0,78; 95 % CI: 0,73 - 0,82; test for subgruppeforskelle p=0,52). Tiltroen til effektestimatet var moderat pga. alvorlig upræcist effektestimat, da der er brede konfidensintervaller.

Fortsat behandling med ICS øger sandsynligvis *risiko for pneumoni*, som er et vigtigt outcome (risk ratio: 1,22; 95 % CI: 0,44 - 3,33), hvilket også blev bekræftet i subgruppeanalysen, hvor vi sammenlignede resultatet med resultatet fra studier, hvor patienterne ikke var selekteret på baggrund af exacabationshistorik (risk ratio: 1,36; 95 % CI: 1,13 - 1,62; test for subgruppeforskelle p=0,83).Tiltroen til effektestimatet er moderat pga. alvorlig upræcist effektestimat, da der er brede konfidensintervaller.

Fortsat behandling med ICS påvirker sandsynligvis ikke det vigtige outcome *lungefunktion* (FEV1 mean difference: 0,01; 95 % CI: -0,01 - 0,04). Tiltroen til effektestimatet er moderat pga. alvorlig upræcist effektestimat, da der er brede konfidensintervaller. Vi fandt ingen studier der opgjorde *antal patienter med forbedring i lungefunktion*. Det er arbejdsgruppens kliniske vurdering at fortsat behandling med ICS sandsynligvis har ingen eller ubetydelig forskel.

Det vigtige outcome *dyspnø* vil muligvis heller ikke blive påvirket af fortsat behandling med ICS (TDI-skala mean difference: -0,12; 95 % CI: -0,71 - 0,47). Tiltroen til effektestimatet er lav pga. alvorlig upræcist effektestimat, da der er brede konfidensintervaller og kun data fra ét studie (INSTEAD), og pga. alvorlig manglende overførbarhed, da der var forskelle mellem ønsket intervention/komparator (ICS+LABA vs. samme LABA) og de i studiet anvendte (ICS + 12 timers virkende LABA vs. anden 24 timers virkende LABA).

I forhold til det vigtige outcome *livskvalitet* var der ikke klinisk relevante forskelle mellem patienter, der fortsatte behandling med ICS sammenlignet med patienter, der fik seponeret ICS (SGRQ-skala mean difference: 1,17; 95 % CI: 1,67 - 0,67). Tiltroen til effektestimatet var høj.

For både de kritiske og vigtige outcomes, kan der på tværs af studierne være risiko for bias pga. gennemgående indblanding i forsøgsdesign og rapportering af resultater fra medicinalindustrien. Der er ikke blevet nedgraderet for dette i nærværende PICO, da dette er gennemgående for alle studier på KOL området og der i øvrigt ikke var andre risiko for bias i studierne.

**Outcome** Tidsramme	**Resultater og målinger**	**Effektestimater** Seponering / ICS	**Tiltro til estimaterne** (at de afspejler den sande effekt i populationen)	**Sammendrag**
**Alvorlige bivirkninger, antal patienter (serious adverse events, number of patients)** Længst mulige follow-up (min. 3 mdr. fra intervention start)<br>9 Kritisk	Relative risiko 1.63 (CI 95% 0.44 - 6.05) Baseret på data fra 1,921 patienter i 3 studier. [1] Opfølgningstid: 26 uger.	**79** per 1.000 / **96** per 1.000<br>Forskel: **17 flere** per 1.000	**Lav** på grund af alvorlig upræcist effektestimat, på grund af alvorlig inkonsistente resultater [2]	Fortsat behandling med ICS øger muligvis antallet af patienter der får alvorlige bivirkninger
**Frafald af alle årsager (All-cause drop-out)** Længst mulige follow-up (min. 3 mdr. fra intervention start)<br>9 Kritisk	Relative risiko 0.83 (CI 95% 0.68 - 1.02) Baseret på data fra 1,896 patienter i 3 studier. [3] Opfølgningstid: 26 uger.	**186** per 1.000 / **160** per 1.000<br>Forskel: **26 færre** per 1.000	**Moderat** på grund af alvorlig upræcist effektestimat [4]	Fortsat behandling med ICS nedsætter sandsynligvis risikoen for frafald af alle årsager
**Pneumoni, antal patienter (Pneumonia, number of patients)** Længst mulige follow-up (min. 3 mdr. fra intervention start)<br>6 Vigtig	Relative risiko 1.22 (CI 95% 0.44 - 3.33) Baseret på data fra 1,921 patienter i 3 studier. [5] Opfølgningstid: 26 uger.	**7** per 1.000 / **10** per 1.000<br>Forskel: **3 flere** per 1.000	**Moderat** på grund af alvorlig upræcist effektestimat [6]	Fortsat behandling med ICS øger sandsynligvis risiko for pneumoni
**Antal patienter med en væsentlig forbedring i lungefunktion (FEV1) (number of patients with improvement in lung function)** Længst mulige follow-up (min. 3 mdr. fra intervention start)<br>6 Vigtig	Baseret på data fra patienter i 0 studier.			Vi fandt ingen studier der opgjorde antal patienter med forbedring i lungefunktion. Det er arbejdsgruppens kliniske vurdering at fortsat behandling med ICS sandsynligvis har ingen eller ubetydelig forskel.

**Outcome** Tidsramme	**Resultater og målinger**	**Effektestimater** Seponering ICS	**Tiltro til estimaterne** (at de afspejler den sande effekt i populationen)	**Sammendrag**
**Lungefunktion (lung function)** Længst mulige follow-up (min. 3 mdr. fra intervention start) 6 Vigtig	Målt med: FEV1 Højere bedre Baseret på data fra: 3,813 patienter i 5 studier. [7] Opfølgningstid: 24-52 uger.	**0.01** (gennemsnit) **0.02** (gennemsnit) Forskel: **MD 0.01 højere** (CI 95% 0.01 lavere - 0.04 højere)	**Moderat** på grund af alvorlig upræcist effektestimat [8]	Fortsat behandling med ICS påvirker sandsynligvis ikke lungefunktion
**Livskvalitet (quality of life)** Længst mulige follow-up (min. 3 mdr. fra intervention start) 6 Vigtig	Målt med: SGRQ Skala: 0-100 Lavere bedre Baseret på data fra: 1,820 patienter i 3 studier. [9] Opfølgningstid: 12-52 uger.	Forskel: **MD 1.17 lavere** (CI 95% 1.67 lavere - 0.67 lavere)	**Høj** [10]	Fortsat behandling med ICS medfører ingen betydelig forbedring i livskvalitet
**Dyspnø (Dyspnea)** Længst mulige follow-up (min. 3 mdr. fra intervention start) 6 Vigtig	Målt med: TDI Skala: -9-9 Højere bedre Baseret på data fra: 468 patienter i 1 studier. [11] Opfølgningstid: 24-26 uger.	Forskel: **MD 0.12 højere** (CI 95% 0.71 lavere - 0.47 højere)	**Lav** på grund af alvorlig upræcist effektestimat, på grund af alvorlig manglende overførbarhed [12]	Fortsat behandling med ICS påvirker muligvis ikke dyspnø
**Moderat til svær exacerbation (exacerbations)** [13] Længst mulige follow-up (min. 3 mdr. fra intervention start) 9 Kritisk	Baseret på data fra: patienter i 2 studier. Opfølgningstid: 24-26 uger.	Rate ratio: 0.67 (CI 95% 0.41 - 1.10)	**Lav** på grund af alvorlig inkonsistente resultater, på grund af alvorlig upræcist effektestimat [14]	Fortsat behandling med ICS nedsætter muligvis risiko for exacerbationer

1. Systematisk oversigtsartikel [46] med inkluderede studier: [42], [44], [43]. rossie, tashkin og doherty. **Baselinerisiko/komparator:** Kontrolarm i reference brugt til interventionen.
2. **Risiko for bias: Ingen betydelig.** risk of bias på grund af gennemgående indblanding i forsøgsdesign og rapportering af resultater fra medicinalindustrien.. **Inkonsistente resultater: Alvorligt.** Den statistiske heterogenicitet er høj, Retningen af effekten er ikke konsistent imellem de inkluderede studier. **Manglende overførbarhed: Ingen betydelig. Upræcist effektest mat: Alvorligt.** Brede konfidensintervaller.
3. Systematisk oversigtsartikel [46] med inkluderede studier: [42], [44], [43]. instead/goyal/rossie, tashkin og doherty. **Baselinerisiko/komparator:** Kontrolarm i reference brugt til interventionen.
4. **Risiko for bias: Ingen betydelig.** risk of bias på grund af gennemgående indblanding i forsøgsdesign og rapportering af resultater fra medicinalindustrien.. **Inkonsistente resultater: Ingen betydelig. Manglende overførbarhed: Ingen betydelig. Upræcist effektest mat: Alvorligt.** Brede konfidensintervaller.
5. Systematisk oversigtsartikel [46] med inkluderede studier: [44], [43], [42]. tashkin, doherty og rossi. **Baselinerisiko/komparator:** Kontrolarm i reference brugt til interventionen.
6. **Risiko for bias: Ingen betydelig.** risk of bias på grund af gennemgående indblanding i forsøgsdesign og rapportering af resultater fra medicinalindustrien.. **Inkonsistente resultater: Ingen betydelig. Manglende overførbarhed: Ingen betydelig. Upræcist effektest mat: Alvorligt.** Brede konfidensintervaller.
7. Systematisk oversigtsartikel [46] med inkluderede studier: [43], [44], [24], [42], [45]. alle 5. **Baselinerisiko/komparator:** Kontrolarm i reference brugt til interventionen.
8. **Risiko for bias: Ingen betydelig.** risk of bias på grund af gennemgående indblanding i forsøgsdesign og rapportering af resultater fra medicinalindustrien.. **Inkonsistente resultater: Ingen betydelig.** Den statistiske heterogenicitet er moderat (I2=51). **Manglende overførbarhed: Ingen betydelig. Upræcist effektest mat: Alvorligt.** Brede konfidensintervaller.
9. Systematisk oversigtsartikel [46] med inkluderede studier: [43], [44], [42]. Rossie, tashkin og doherty. **Baselinerisiko/komparator:** Kontrolarm i reference brugt til interventionen.
10. **Risiko for bias: Ingen betydelig.** risk of bias på grund af gennemgående indblanding i forsøgsdesign og rapportering af resultater fra medicinalindustrien.. **Inkonsistente resultater: Ingen betydelig. Manglende overførbarhed: Ingen betydelig. Upræcist effektest mat: Ingen betydelig.**
11. Systematisk oversigtsartikel [46] med inkluderede studier: [44]. Rossie. **Baselinerisiko/komparator:** Kontrolarm i reference brugt til interventionen.
12. **Risiko for bias: Ingen betydelig.** risk of bias på grund af gennemgående indblanding i forsøgsdesign og rapportering af resultater fra medicinalindustrien.. **Inkonsistente resultater: Ingen betydelig. Manglende overførbarhed: Alvorligt.** Forskelle mellem ønsket intervention/komparator og de i studiet anvendte. **Upræcist effektest mat: Alvorligt.** Kun data fra ét studie, Brede konfidensintervaller.
13. Baseret på data fra 2 studier. Opfølgningstid: 24-26 uger.
14. **Risiko for bias: Ingen betydelig.** risk of bias på grund af gennemgående indblanding i forsøgsdesign og rapportering af resultater fra medicinalindustrien.. **Inkonsistente resultater: Alvorligt.** Den statistiske heterogenicitet er høj. **Upræcist effektest mat: Alvorligt.** Brede konfidensintervaller.

Referencer

24. Chapman KR, Hurst JR, Frent S-M, Larbig M, Fogel R, Guerin T, et al. : Long-Term Triple Therapy De-escalation to Indacaterol/Glycopyrronium in Patients with Chronic Obstructive Pulmonary Disease (SUNSET): A Randomized, Double-Blind, Triple-Dummy Clinical Trial. American Journal of Respiratory & Critical Care Medicine 2018;198(3):329-339

42. Doherty DE, Tashkin DP, Kerwin E, Knorr BA, Shekar T, Banerjee S, et al. : Effects of mometasone furoate/formoterol fumarate fixed-dose combination formulation on chronic obstructive pulmonary disease (COPD): results from a 52-week Phase III trial in subjects with moderate-to-very severe COPD. International journal of chronic obstructive pulmonary disease 2012;7 57-71 Pubmed Journal

43. Tashkin DP, Doherty DE, Kerwin E., Matiz-Bueno CE, Knorr B., Shekar T., et al. : Efficacy and safety characteristics of mometasone furoate/formoterol fumarate fixed-dose combination in subjects with moderate to very severe COPD: findings from pooled analysis of two randomized, 52-week placebo-controlled trials. International journal of chronic obstructive pulmonary disease 2012;7 73-86 Journal

44. Rossi A, van der Molen T, del Olmo R, Papi A, Wehbe L, Quinn M, et al. : INSTEAD: a randomised switch trial of indacaterol versus salmeterol/fluticasone in moderate COPD. The European respiratory journal 2014;44(6):1548-56 Journal Link

45. Martinez F., Ferguson G., Bourne E., Ballal S., Darken P., DeAngelis K., et al. : Budesonide/glycopyrrolate/formoterol fumarate metered dose inhaler, formulated using co-suspension delivery technology, improves lung function and exacerbation outcomes in patients with COPD without a recent history of exacerbations: subgroup analysis of KRONOS study. Chest 2019;156(4):A2276-A2277 Journal Link

46. NKR Pulje PICO 4: ICS til patienter med KOL efter mindst et Ã¥r uden moderate/alvorlige exacerbationer.

5 - Patienter med KOL i behandling med ICS og med hyppige, tilbagevendende pneumonier

PICO 5
Bør patienter med kronisk obstruktiv lungesygdom (KOL) og hyppige tilbagevendende pneumonier fortsat behandles med inhalationssteroid (ICS)?

Baggrund for valg af spørgsmålet
Nogle patienter med KOL har en øget risiko for at udvikle pneumoni *[49]*. Pneumoni hos patienter med KOL er forbundet med en øget risiko for exacerbationer, fald i FEV1 og progression af sygdommen *[1]*. Dette sammenholdt med, at studier har vist, at behandling med ICS øger risiko for pneumoni *[50][51]*, gør det derfor relevant at undersøge om patienter med KOL og tendens til pneumoni, fortsat vil have gavn af behandling med ICS. Arbejdsgruppen har valgt at patienter med KOL og mindst to pneumonier det seneste år, må betragtes som havende tendens til hyppige pneumonier, og dermed øget pneumonirisiko.

Svag anbefaling mod

Fortsæt kun behandlingen med inhalationssteroid (ICS) til patienter med kronisk obstruktiv lungesygdom (KOL) og hyppige, tilbagevendende pneumonier, efter nøje overvejelser.

Patienter med KOL og hyppige, tilbagevendende pneumonier forventes kun at have sparsom effekt af fortsat behandling med ICS. Det er arbejdsgruppens vurdering, at risikoen for pneumoni må opvejes mod risiko for exacerbationer hos den enkelte.

Ingen studier omfattede risiko for moderat til svær exacerbation, frafald eller bivirkninger for denne gruppe patienter.

Et enkelt studie viser en øget risiko for pneumoni på 146 % (konfidensinterval 47-312 %).

Arbejdsgruppen anbefaler på baggrund af klinisk erfaring at seponere ICS gradvist i samarbejde med patienten. Fx reducér til halv dosis og fortsæt i 6-8 uger efterfulgt af seponering. Kan patienten ikke undvære ICS, anbefaler arbejdsgruppen, at behandlingen genoptages i lavest mulige effektive dosis. Se i øvrigt DLS' KOL vejledning.

Nøgleinformationer

Gavnlige og skadelige virkninger — Lille netto gevinst eller små forskelle mellem alternativerne

Det er usikkert om fortsat behandling med ICS øger risikoen for pneumoni, men øget sandsyndligheden i et enkelt studie (HR: 2.46 (95 % CI: 1.47 - 4.12)). Tiltroen til estimatet er lav pga. manglende overførbarhed og upræcist effektestimat.

Der var ingen studier som opgjorde de øvrige outcomes.

Kvaliteten af evidensen — Meget lav

Der er meget lav tiltro til evidensen for det kritiske outcome *pneumoni*. Tiltroen til evidensen er nedgraderet pga. at der kun var ét studie, brede konfidensintervaller, mulige forskelle mellem målpopulation og studiepopulation og pga. manglende definition af pneumonihistorie det forudgående år. I studiet bestod populationen af patienter, som havde oplevet mindst én pneumoni det seneste år, mens den forud definerede population i dette PICO var patienter, som havde oplevet mindst to pneumonier det seneste år.

Der er ikke fundet evidens for de øvrige outcomes.

Patientpræferencer — Betydelig variation er forventet eller usikker

Det er arbejdsgruppens vurdering at der vil være betydelig variation.

Rationale
Der blev i formuleringen af anbefalingen lagt vægt på at fortsat behandling med ICS øger risiko for pneumoni. Der forventes betydelig variation i patientpræferencer.

Fokuseret Spørgsmål

Population: Patienter på 40 år eller derover med KOL, som ikke er nuværende astmapatienter, har haft minimum 2 pneumonier indenfor det seneste år og som har været i behandling med ICS mindst 3 måneder inden og/eller under forsøget.

Intervention: Behandling med ICS dagligt i mindst 3 måneder (gælder både nyopstartet eller fortsat behandling med ICS) – enten som monoterapi eller i tilgift til behovsmedicin eller anden inhalationsbehandling (ikke ICS).

Sammenligning: Ingen ICS-behandling (men kan have fået ICS-behandlingen forud for forsøgsopstart). Patienter kan være i behandling med behovsmedicin eller få anden inhalationsbehandling (ikke ICS).

Sammenfatning

Litteratur
Evidensgrundlaget for PICO 5 er et randomiseret forsøg *[50]*.

For at undersøge robustheden af overstående studie og få en indikation på nogle outcomes uden evidens, blev der foretaget en sensitivitetsanalyse på følgende outcomes: risiko for moderat til svær exacerbation, pneumoni, lungefunktion og frafald af alle årsager. Til sensitivitetsanalyse blev der via litteraturgennemgang fundet 22 randomiserede forsøg *[16][24][25][26][27][28][29][30][31][32][33][43][54][56][57][59][47][62][64][65][67][68]*. Disse forsøg have ikke analyseret patienter med hyppige tilbagevendende pneumonier, men overholdt i øvrigt vores inklusionskriterier.

Flow charts findes her.

Gennemgang af evidensen
Det inkluderede studie var et randomiseret kontrolleret forsøg. I studiet deltog 3.255 patienter, der blev randomiseret til enten en af interventionsgrupperne, der blev behandlet med vilanterol (VI) 25 mg kombineret med hhv. 50, 100, eller 200 mg fluticasone furoate (FF) eller sammenligningsgruppen, der kun fik VI 25 mg. Interventionen varede i 52 uger. Patienterne var primært mænd, og havde en gennemsnitsalder på 63,7 (SD: 9,2) år, et gennemsnitligt BMI på 26,9 (SD: 5,9) kg/m2, og lige under halvdelen var nuværende rygere. Ved inklusion var FEV1 i gennemsnit ca. 45 % af forventet. Det er uklart hvor mange pneumonier patienterne havde haft indenfor det seneste år.

Pneumoni/exacerbation
Alle definitioner af exacerbation og pneumoni fra studierne er inkluderet.

Resultater
Studiet afrapporterede kun på et enkelt at vores prædefinerede outcomes, og der er dermed kun data på det kritiske outcome pneumoni. Resultatet viste en øget risiko for *pneumoni* ved fortsat behandling med ICS (hazard ratio: 2,46; 95 % CI: 1,47 - 4,12). Tiltroen til effektestimatet er meget lav pga. meget alvorlig upræcist effektestimat, da der var brede konfidensintervaller og kun data fra ét studie, og pga. meget alvorlig manglende overførbarhed, da der muligvis er forskelle mellem målpopulation og studiepopulation (studiet har ikke nærmere defineret pneumonihistorie det forudgående år og målpopulationen er patienter med mindst to pneumonier det forudgående år). For dette outcome kan der være risiko for bias pga. gennemgående indblanding i forsøgsdesign og rapportering af resultater fra medicinalindustrien. Der er ikke blevet nedgraderet for dette i nærværende PICO, da dette er gennemgående for alle studier på KOL-området og der i øvrigt ikke var andre risiko for bias i studierne.

Sensitivitetsanalysen viste en nedsat *risiko for moderat til svær exacerbation* ved forsat behandling med ICS (rate ratio: 0,76; 95 % CI: 0,71 - 0,82). Tiltroen til effektestimatet er dog lav pga. alvorlig risiko for bias og alvorlig manglende overførbarhed, da der er forskelle mellem målpopulation og studiepopulation (studiepopulationen er ikke analyseret på baggrund af pneumonihistorik).

Ligeledes viste sensitivitetsanalyse et mindre *frafald af alle årsager* ved forsat behandling med ICS (risk ratio: 0,78; 95 % CI: 0,74 - 0,83). Tiltroen til effektestimatet er dog lav pga. alvorlig risiko for bias og alvorlig manglende overførbarhed, da der er forskelle mellem målpopulation og studiepopulation (studiepopulationen er ikke analyseret på baggrund af pneumonihistorik).

I forhold til *lungefunktion*, viste sensitivitetsanalysen ingen klinisk relevant forskel ved fortsat behandling med ICS (FEV1 mean difference: 0,03 L; 95 % CI: 0,01 - 0,05 L). Tiltroen til effektestimatet er dog meget lav pga. alvorlig risiko for bias, alvorligt upræcist effektestimat og alvorlig manglende overførbarhed, da der er forskelle mellem målpopulation og studiepopulation (studiepopulationen er ikke analyseret på baggrund af pneumonihistorik).

**Outcome** Tidsramme	**Resultater og målinger**	**Effektestimater** Seponering	ICS	**Tiltro til estimaterne** (at de afspejler den sande effekt i populationen)	**Sammendrag**
**Pneumoni (Pneumonia)** Længst mulige follow-up (min. 3 mdr. fra intervention start) 9 Kritisk	Hazard ratio 2.46 (CI 95% 1.47 - 4.12) Baseret på data fra 3,255 patienter i 1 studier. [1] Opfølgningstid: 52 uger.			**Meget lav** på grund af meget alvorlig upræcist effektestimat, på grund af meget alvorlig manglende overførbarhed [2]	Vi er usikre på, om fortsat behandling med ICS øger risikoen for pneumoni.
**Moderat til svær exacerbation (exacerbations)** Længst mulige follow-up (min. 3 mdr. fra intervention start) 9 Kritisk	Baseret på data fra patienter i 0 studier.				Vi fandt ingen studier der opgjorde risiko for exacerbationer. Det er arbejdsgruppens kliniske vurdering at fortsat behandling med ICS sandsynligvis vil reducere risiko for exacerbationer.
**Alvorlige bivirkninger, antal patienter (serious adverse events, number of patients)** Længst mulige follow-up (min. 3 mdr. fra intervention start) 9 Kritisk	Baseret på data fra patienter i 0 studier.				Vi fandt ingen studier der opgjorde antal patienter der får alvorlige bivirkninger. Det er arbejdsgruppens kliniske vurdering at fortsat behandling med ICS sandsynligvis ikke øger antal patienter der får alvorlige bivirkninger i betydelig grad.
**Frafald af alle årsager (All-cause drop-out)** Længst mulige follow-up (min. 3 mdr. fra intervention start) 6 Vigtig	Baseret på data fra patienter i 0 studier.				Vi fandt ingen studier der opgjorde frafald. Det er arbejdsgruppens kliniske vurdering at fortsat behandling med ICS sandsynligvis vil reducere frafald.

**Outcome** Tidsramme	**Resultater og målinger**	**Effektestimater** Seponering	ICS	**Tiltro til estimaterne** (at de afspejler den sande effekt i populationen)	**Sammendrag**
**Antal patienter med en væsentlig forbedring i lungefunktion (FEV1) (number of patients with improvement in lung function)** Længst mulige follow-up (min. 3 mdr. fra intervention start) 6 Vigtig	Baseret på data fra patienter i 0 studier.				Vi fandt ingen studier der opgjorde antal patienter med forbedring i lungefunktion. Arbejdsgruppen er usikre på om fortsat behandling med ICS øger antallet af patienter med en væsentlig forbedring i lungefunktion
**Lungefunktion (lung function)** Længst mulige follow-up (min. 3 mdr. fra intervention start) 6 Vigtig	Baseret på data fra: patienter i 0 studier.				Vi fandt ingen studier der opgjorde lungefunktionen. Arbejdsgruppen er usikre på om fortsat behandling med ICS forbedrer lungefunktion
**Dyspnø (Dyspnea)** Længst mulige follow-up (min. 3 mdr. fra intervention start) 6 Vigtig	Baseret på data fra: patienter i 0 studier.				Vi fandt ingen studier der opgjorde dyspnø. Arbejdsgruppen er usikre på om fortsat behandling med ICS ændre graden af dyspnø
**Livskvalitet (quality of life)** Længst mulige follow-up (min. 3 mdr. fra intervention start) 6 Vigtig	Baseret på data fra: patienter i 0 studier.				Vi fandt ingen studier der opgjorde livskvalitet. Arbejdsgruppen er usikre på om fortsat behandling med ICS påvirker livskvaliteten.

1. Systematisk oversigtsartikelmed inkluderede studier: [50]. **Baselinerisiko/komparator:** Kontrolarm i reference brugt til interventionen.
2. **Risiko for bias: Ingen betydelig.** risk of bias på grund af gennemgående indblanding i forsøgsdesign og rapportering af resultater fra medicinalindustrien.. **Inkonsistente resultater: Ingen betydelig. Manglende overførbarhed: Meget alvorligt.** Muligvis forskelle mellem målpopulation og studiepopulation, på grund af manglende definition af pneumonihistorie det forudgående år. **Upræcist effektestimat: Meget alvorligt.** Kun data fra ét studie, Brede konfidensintervaller..

Referencer

50. Crim C., Dransfield MT, Bourbeau J., Jones PW, Hanania NA, Mahler DA, et al. : Pneumonia risk with inhaled fluticasone furoate and vilanterol compared with vilanterol alone in patients with COPD. Annals of the American Thoracic Society 2015;12(1):27-34 Journal

Bilag 1: Implementering

Regionerne og regionernes sygehuse spiller en vigtig rolle i at understøtte implementeringen af den nationale kliniske retningslinje gennem formidling af retningslinjens indhold og ved at understøtte retningslinjens anvendelse i praksis. For at understøtte retningslinjens anvendelse lokalt er det hensigtsmæssigt, at den nationale kliniske retningslinje samstemmes med eller integreres i de forløbsbeskrivelser, instrukser og vejledninger, som allerede anvendes her. Regionerne bør således sikre, at de anbefalinger, som må være relevante for specialiserede afdelinger på sygehusniveau, indarbejdes i instrukser og vejledninger i den pågældende region.

For almen praksis indebærer det, at anbefalinger fra den nationale kliniske retningslinje indarbejdes i regionernes forløbsbeskrivelser for KOL og Basislisten for KOL. Således vil de evidensbaserede relevante anbefalinger indgå i de patientvejledninger, som alment praktiserende læger allerede anvender, og som forholder sig til organisering i øvrigt i den pågældende region. Forløbsbeskrivelserne kan med fordel indeholde et link til den fulde nationale kliniske retningslinje. Der opfordres til at man i samarbejde med patienten inddrager disse anbefalinger i udarbejdelsen af forløbsplaner for KOL.

Herudover kan der med fordel indsættes et link til den nationale kliniske retningslinje i Lægehåndbogen. Regionernes lægemiddelenheder og praksiskonsulenter kan desuden have en rolle i at tage stilling til den konkrete implementering. Derudover er det oplagt, at man arbejder med dette i de etablerede klyngesamarbejder. På baggrund af denne nationale kliniske retningslinje vil Medicinfunktionen udarbejde et materiale til almen praksis om seponering af ICS hos patienter med KOL. Materialet vil være frit tilgængelig på Medicinfunktionen.dk.

De faglige selskaber er en vigtig aktør i at udbrede kendskabet til retningslinjen. Der foreslås, at den nationale kliniske retningslinje omtales på de relevante faglige selskabers hjemmeside, evt. med orientering om, hvad den indebærer for det pågældende speciale og med et link til den fulde version af retningslinjen. Her tænkes særligt på Dansk Lungemedicinsk Selskab og Dansk Selskab for Almen Medicin. Der opfordres ligeledes til, at retningslinjen præsenteres på årsmøder i regi af de faglige selskaber og på lægedage. Information kan også formidles via medlemsblade og elektroniske nyhedsbreve.

Desuden foreslås, at retningslinjens indhold formidles til patienterne, og at relevante patientforeninger som Lungeforeningen kan spille en rolle heri.

Implementering af den nationale kliniske retningslinje for behandling med ICS hos patienter med KOL er som udgangspunkt et regionalt og kommunalt ansvar. Dog ønsker Sundhedsstyrelsen at understøtte implementeringen. I foråret 2014 publicerede Sundhedsstyrelsen således en værktøjskasse med konkrete redskaber til implementering. Den er tilgængelig som et elektronisk opslagsværk på www.sst.dk. Værktøjskassen bygger på evidensen for effekten af interventioner, og den er tænkt som en hjælp til lederen eller projektlederen, der lokalt skal arbejde med implementering af forandringer af et vist omfang.

Bilag 2: Monitorering

Patienter med kronisk obstruktiv lungesygdom (KOL; ICD10 J44) bliver registreret i Dansk Register for Kronisk Obstruktiv Lungesygdom (DrKOL), gennem kontakt med hospitalet og/eller registrering fra almen praksis (fra 2020). Det kan være en udfordring at identificere de subpopulationer, der er omtalt i denne nationale kliniske retningslinje. For at kunne monitorere proces- og effektindikatorer er det derfor nødvendigt at benytte flere databaser og kæde deres data sammen.

Proces- og effektindikatorer

Som procesindikatorer kan bruges følgende:

- Implementering af retningslinjen på regionernes basisliste
- Andel af patienter med KOL, der behandles med ICS
- Andel af patienter med KOL, som ikke har haft moderate til svære KOL exacerbationer det sidste år, der behandles med ICS
- Andel af patienter med KOL og stabile blod-eosinofilocytter fordelt på <150, 150-300 og >300, der behandles med ICS
- Andel af patienter med KOL, som har fået antibiotikabehandling svarende til pneumoni (J01CR02, J01CA04, J01CA02, J01CE02, J01FA01, J01FA06, J01FA09, J01FA10) mindst to gange det sidste år, der behandles med ICS

Som effektindikatorer kan bruges følgende:

- Andelen af patienter med KOL, som får lægemidler mod mundsvamp
- Andelen af patienter med KOL, som indlægges med pneumoni som hoveddiagnose
- Omsætningen pr. patient med KOL til lægemidler i ATC-gruppen R03

Datakilder

Arbejdsgruppen anbefaler at bruge data fra Landspatientregisteret, Dansk Register for Kronisk Obstruktiv Lungesygdom (DRKOL), Lægemiddelstatistikregisteret og Laboratoriedatabasen

Bilag 3: Opdatering og videre forskning

Opdatering

Det er forholdsvist nyt at anvende blod-eosinofilocytter (EOS) som hjælperedskab i vurderingen af, hvilke patienter med KOL, der vil have glæde af fortsat behandling med inhalationssteroid (ICS). Med denne retningslinje er den nuværende evidens blevet belyst og vurderet, men det står samtidig klart, at det meste af vores nuværende viden stammer fra retrospektive (post hoc) analyser. Der er, for nuværende, ganske få prospektive, randomiserede studier på området. Imidlertid vurderes det, at en del studier i de kommende år vil adressere problemstillinger, som vil være relevant for forståelsen af sammenspillet mellem EOS og ICS i forhold til KOL-exacerbationer. Derfor vil det være hensigtsmæssigt, om vejledningen blev genbesøgt og opdateret indenfor en femårig tidsperiode af en arbejdsgruppe under Dansk Lungemedicinsk Selskab (DLS) og Medicinfunktionen, Klinisk Farmakologisk Afdeling, Region Hovedstaden.

Videre forskning

Denne retningslinje har klarlagt, at den eksisterende videnskabelige litteratur på området er mangelfuld. Resultatet fra denne retningslinje tyder på, at effekten af ICS stiger med niveauet af EOS. Men der er mange ubesvarede spørgsmål. For eksempel i forhold til hvad den optimale steroiddosis er, hvornår EOS bedst måles og om målingerne bør gentages, og i så fald i hvilket interval. Arbejdet med denne retningslinje har også vist, at forskningsområdet indenfor KOL har en udfordring med indblanding fra medicinalindustrien. Dette gør på den ene side at der findes store omfattende undersøgelser, men på den anden side er indblandingen ikke kun i form af finansiering og planlægning af studiet, men også i fortolkning og publicering af resultaterne, hvilket øger risiko for bias.

- Har inhalationssteroid signifikant og klinisk relevant effekt på symptomer og/eller livskvalitet hos patienter med KOL, og er effekten afhængig af EOS?
- Hvilken dosis af inhalationssteroid er optimal for behandling af hhv. exacerbationer, åndenød og livskvalitet for patienter med KOL?
- Er der en nedre grænse for EOS, hvorunder den samlede effekt af steroidbehandling (inhaleret og/eller peroral) er negligerbar?
- Har patienter med KOL, som aldrig har haft moderat til svær exacerbation gavn af ICS?
- Er ændring i lungefunktion drevet af få individer, som har en stor ændring, eller mange patienter, som har en moderat effekt?
- Hvor stor øget risiko for pneumoni har patienter, som tidligere har haft pneumoni, ved fortsat behandling med ICS?
- Hvordan påvirker behandling med ICS patientens EOS?
- Hvordan påvirker tobak patienternes EOS?

Bilag 4: Beskrivelse af anvendt metode

Den anvendte graduering af evidensens kvalitet og anbefalingsstyrke baserer sig på GRADE (Grading of Recommendations Assessment, Development and Evaluation). Se også: http://www.gradeworkinggroup.org

Sundhedsstyrelsen har udarbejdet en metodehåndbog for nationale kliniske retningslinjer som kan tilgås via www.sst.dk. Metodehåndbogen indeholder en beskrivelse af den metodiske tilgang og processen for udarbejdelse af evidensbasererede nationale kliniske retningslinjer., som er blevet anvendt i nærværende kliniske retningslinje.

Bilag 5: PICO

PICO 1: Bør patienter med KOL og et blodeosinofiltal under 150 celler/ µL fortsat behandles med ICS?

Baggrund for valg af spørgsmål:

Inhalationssteroid (ICS) har antiinflammatorisk virkning, som formodes at være virkningsmekanismen ved behandling af patienter med kronisk obstruktiv lungesygdom (KOL) *[8]*. Studier har vist, at niveauet af eosinofilocytter i sputum kan korreleres til graden af inflammation i lungerne *[9]* og at niveauet af eosinofilocytter i sputum hos patienter med KOL kan være prædiktiv for effekten af systemisk steroid såvel som inhalationssteroid *[8][10][11][12][13]*. Målinger af eosinofilocytter i sputum er en omstændelig proces, der ikke vil være rutinemæssig mulig de fleste steder - herunder i almen praksis. Derfor bruges eosinofilocytter i blod (EOS) i denne retningslinje og i de fleste studier. EOS måles gennem en blodprøve og er derfor en let tilgængelig og billig måde at måle inflammation på.

Ved exacerbationer som følge af KOL har studier vist, at patienter med EOS over 2 % responderer bedre på behandling med systemisk steroid, end patienter med EOS under 2 % *[12][14]*. I Danmark, og de fleste andre steder, anvendes det absolutte antal EOS (celler/ µL), i stedet for det relative EOS. Når der bestilles leukocytter med differentialtælling er det således det absolutte antal EOS, der oplyses fra laboratoriet. Global Initiative for Chronic Obstructive Lung Disease (GOLD) har siden 2019 *[15]* anbefalet at bruge absolut EOS til at vurdere, om patienter med svær KOL og hyppige exacerbationer skal opstartes i behandling med ICS.

For patienter med KOL, som er i behandling med ICS, virker EOS derfor som en mulig relevant biomarkør at inddrage i beslutningsprocessen om behandling med ICS skal fortsættes eller seponeres. Dog er der endnu ikke konsensus om grænseværdier. Et studie peger på at patienter med en EOS på over 300 celler/uL har effekt af behandling med ICS, hvorimod patienter med EOS på under 100 celler/uL har beskeden effekt *[16]*. Et dansk studie på en repræsentativ befolkningsgruppe fra København (i forhold til køn og alder) fandt, at den gennemsnitlige EOS var 180 celler/µL for patienter med KOL *[17]*. Arbejdsgruppen har derfor valgt at undersøge effekten af forsat behandling af ICS til patienter med KOL og med EOS på hhv. under 150, mellem 150-300 og over 300 celler/µL.

Population (population

Patienter på 40 år eller derover med KOL, som har et blodeosinofiltal på under 150 celler/µL, ikke er nuværende astmapatienter og som har været i behandling med ICS mindst 3 måneder inden og/eller under forsøget.

Intervention

Behandling med ICS dagligt i mindst 3 måneder (gælder både nyopstartet eller fortsat behandling med ICS) – enten som monoterapi eller i tilgift til behovsmedicin eller anden inhalationsbehandling (ikke ICS).

Comparison (sammenligning

Ingen ICS-behandling (men kan have fået ICS-behandlingen forud for forsøgsopstart). Patienter kan være i behandling med behovsmedicin eller få anden inhalationsbehandling (ikke ICS).

Outcome	Tidsramme	Kritisk/ Vigtigt
Exacerbationer pr. år (moderat eller alvorlige)	Længst mulige follow-up (min. 3 mdr. fra intervention start)	Kritisk
Antal patienter med alvorlige bivirkninger pr. år (SAE)	Længst mulige follow-up (min. 3 mdr. fra intervention start)	Kritisk
Pneumoni pr. år	Længst mulige follow-up (min. 3 mdr. fra intervention start)	Vigtigt
Lungefunktion (FEV_1)	Længst mulige follow-up (min. 3 mdr. fra intervention start)	Vigtigt
Dyspnø (fx MRC)	Længst mulige follow-up (min. 3 mdr. fra intervention start)	Vigtigt

Livskvalitet (Fx SGRQ-C, CCQ eller anden relevant test)	Længst mulige follow-up (min. 3 mdr. fra intervention start)	Vigtigt
Frafald	Længst mulige follow-up (min. 3 mdr. fra intervention start)	Kritisk
Antal patienter med en væsentlig forbedring i lungefunktion (FEV_1)	Længst mulige follow-up (min. 3 mdr. fra intervention start)	Vigtigt

PICO 2: Bør patienter med KOL og et blodeosinofiltal mellem 150 og 300 celler/µL fortsat behandles med ICS?

Baggrund for valg af spørgsmål:

Inhalationssteroid (ICS) har antiinflammatorisk virkning, som formodes at være virkningsmekanismen ved behandling af patienter med kronisk obstruktiv lungesygdom (KOL) *[8]*. Studier har vist, at niveauet af eosinofilocytter i sputum kan korreleres til graden af inflammation i lungerne *[9]* og at niveauet af eosinofilocytter i sputum hos patienter med KOL kan være prædiktiv for effekten af systemisk steroid såvel som inhalationssteroid *[8][10][11][12][13]*. Målinger af eosinofilocytter i sputum er en omstændelig proces, der ikke vil være rutinemæssig mulig de fleste steder - herunder i almen praksis. Derfor bruges eosinofilocytter i blod (EOS) i denne retningslinje og i de fleste studier. EOS måles gennem en blodprøve og er derfor en let tilgængelig og billig måde at måle inflammation på.

Ved exacerbationer som følge af KOL har studier vist, at patienter med EOS over 2 % responderer bedre på behandling med systemisk steroid, end patienter med EOS under 2 % *[12][14]*. I Danmark, og de fleste andre steder, anvendes det absolutte antal EOS (celler/µL), i stedet for det relative EOS. Når der bestilles leukocytter med differentialtælling er det således det absolutte antal EOS, der oplyses fra laboratoriet. Global Initiative for Chronic Obstructive Lung Disease (GOLD) har siden 2019 *[15]* anbefalet at bruge absolut EOS til at vurdere, om patienter med svær KOL og hyppige exacerbationer skal opstartes i behandling med ICS.

For patienter med KOL, som er i behandling med ICS, virker EOS derfor som en mulig relevant biomarkør at inddrage i beslutningsprocessen om behandling med ICS skal fortsættes eller seponeres. Dog er der endnu ikke konsensus om grænseværdier. Et studie peger på at patienter med en EOS på over 300 celler/uL har effekt af behandling med ICS, hvorimod patienter med EOS på under 100 celler/uL har beskeden effekt *[16]*. Et dansk studie på en repræsentativ befolkningsgruppe fra København (i forhold til køn og alder) fandt, at den gennemsnitlige EOS var 180 celler/µL for patienter med KOL *[17]*.
Arbejdsgruppen har derfor valgt at undersøge effekten af forsat behandling af ICS til patienter med KOL og med EOS på hhv. under 150, mellem 150-300 og over 300 celler/µL.

Population (population

Patienter på 40 år eller derover med KOL, som har et blodeosinofiltal på mellem 150 og 300 celler/µL, ikke er nuværende astmapatienter og som har været i behandling med ICS mindst 3 måneder inden og/eller under forsøget.

Intervention

Behandling med ICS dagligt i mindst 3 måneder (gælder både nyopstartet eller fortsat behandling med ICS) – enten som monoterapi eller i tilgift til behovsmedicin eller anden inhalationsbehandling (ikke ICS).

Comparison (sammenligning

Ingen ICS-behandling (men kan have fået ICS-behandlingen forud for forsøgsopstart). Patienter kan være i behandling med behovsmedicin eller få anden inhalationsbehandling (ikke ICS).

**Outcome**	**Tidsramme**	**Kritisk/ Vigtigt**
Exacerbationer pr. år (moderat eller alvorlige)	Længst mulige follow-up (min. 3 mdr. fra intervention start)	Kritisk
Antal patienter med alvorlige bivirkninger pr. år (SAE)	Længst mulige follow-up (min. 3 mdr. fra intervention start)	Kritisk

Pneumoni pr. år	Længst mulige follow-up (min. 3 mdr. fra intervention start)	Vigtigt
Lungefunktion (FEV_1)	Længst mulige follow-up (min. 3 mdr. fra intervention start)	Vigtigt
Dyspnø (fx MRC)	Længst mulige follow-up (min. 3 mdr. fra intervention start)	Vigtigt
Livskvalitet (Fx SGRQ-C, CCQ eller anden relevant test)	Længst mulige follow-up (min. 3 mdr. fra intervention start)	Vigtigt
Frafald	Længst mulige follow-up (min. 3 mdr. fra intervention start)	Kritisk
Antal patienter med en væsentlig forbedring i lungefunktion (FEV1)	Længst mulige follow-up (min. 3 mdr. fra intervention start)	Vigtigt

PICO 3: Bør patienter med KOL og et blodeosinofiltal over 300 celler/µL fortsat behandles med ICS?

Baggrund for valg af spørgsmål:

Inhalationssteroid (ICS) har antiinflammatorisk virkning, som formodes at være virkningsmekanismen ved behandling af patienter med kronisk obstruktiv lungesygdom (KOL) *[8]*. Studier har vist, at niveauet af eosinofilocytter i sputum kan korreleres til graden af inflammation i lungerne *[9]* og at niveauet af eosinofilocytter i sputum hos patienter med KOL kan være prædiktiv for effekten af systemisk steroid såvel som inhalationssteroid *[8][10][11][12][13]*. Målinger af eosinofilocytter i sputum er en omstændelig proces, der ikke vil være rutinemæssig mulig de fleste steder - herunder i almen praksis. Derfor bruges eosinofilocytter i blod (EOS) i denne retningslinje og i de fleste studier. EOS måles gennem en blodprøve og er derfor en let tilgængelig og billig måde at måle inflammation på.

Ved exacerbationer som følge af KOL har studier vist, at patienter med EOS over 2 % responderer bedre på behandling med systemisk steroid, end patienter med EOS under 2 % *[12][14]*. I Danmark, og de fleste andre steder, anvendes det absolutte antal EOS (celler/µL), i stedet for det relative EOS. Når der bestilles leukocytter med differentialtælling er det således det absolutte antal EOS, der oplyses fra laboratoriet. Global Initiative for Chronic Obstructive Lung Disease (GOLD) har siden 2019 *[15]* anbefalet at bruge absolut EOS til at vurdere, om patienter med svær KOL og hyppige exacerbationer skal opstartes i behandling med ICS.

For patienter med KOL, som er i behandling med ICS, virker EOS derfor som en mulig relevant biomarkør at inddrage i beslutningsprocessen om behandling med ICS skal fortsættes eller seponeres. Dog er der endnu ikke konsensus om grænseværdier. Et studie peger på at patienter med en EOS på over 300 celler/uL har effekt af behandling med ICS, hvorimod patienter med EOS på under 100 celler/uL har beskeden effekt *[16]*. Et dansk studie på en repræsentativ befolkningsgruppe fra Købehavn (i forhold til køn og alder) fandt, at den gennemsnitlige EOS var 180 celler/µL for patienter med KOL *[17]*. Arbejdsgruppen har derfor valgt at undersøge effekten af forsat behandling af ICS til patienter med KOL og med EOS på hhv. under 150, mellem 150-300 og over 300 celler/µL.

Population (population)

Patienter på 40 år eller derover med KOL, som har et blodeosinofiltal på over 300 celler/µL, ikke er nuværende astmapatienter og som har været i behandling med ICS mindst 3 måneder inden og/eller under forsøget.

Intervention

Behandling med ICS dagligt i mindst 3 måneder (gælder både nyopstartet eller fortsat behandling med ICS) – enten som monoterapi eller i tilgift til behovsmedicin eller anden inhalationsbehandling (ikke ICS).

Comparison (sammenligning)

Ingen ICS-behandling (men kan have fået ICS-behandlingen forud for forsøgsopstart). Patienter kan være i behandling med behovsmedicin eller få anden inhalationsbehandling (ikke ICS).

Outcome	Tidsramme	Kritisk/ Vigtigt
Exacerbationer pr. år (moderat eller alvorlige)	Længst mulige follow-up (min. 3 mdr. fra intervention start)	Kritisk
Antal patienter med alvorlige bivirkninger pr. år (SAE)	Længst mulige follow-up (min. 3 mdr. fra intervention start)	Kritisk
Pneumoni pr. år	Længst mulige follow-up (min. 3 mdr. fra intervention start)	Vigtigt
Lungefunktion (FEV_1)	Længst mulige follow-up (min. 3 mdr. fra intervention start)	Vigtigt
Dyspnø (fx MRC)	Længst mulige follow-up (min. 3 mdr. fra intervention start)	Vigtigt
Livskvalitet (Fx SGRQ-C, CCQ eller anden relevant test)	Længst mulige follow-up (min. 3 mdr. fra intervention start)	Vigtigt
Frafald	Længst mulige follow-up (min. 3 mdr. fra intervention start)	Kritisk
Antal patienter med en væsentlig forbedring i lungefunktion (FEV1)	Længst mulige follow-up (min. 3 mdr. fra intervention start)	Vigtigt

PICO 4: Bør patienter med KOL efter mindst et år uden moderate/ alvorlige exacerbationer fortsat behandles med ICS?

Baggrund for valg af spørgsmål:

ICS kan reducere risiko for exacerbation hos patienter med KOL *[38][39]* og behandling med ICS anbefales derfor til patienter med hyppige exacerbationer *[1]*. Det er derfor nærliggende at undersøge effekten af fortsat behandling med ICS hos de patienter med KOL, som har lav risiko for exacerbation. Det er svært at forudse, hvilke patienter, der har en særlig lav risiko for at få fremtidige exacerbationer, og for nu er den bedste indikator for fremtidig exacerbationer, hvorvidt patienten har haft tidligere exacerbationer *[40]*. Patienter med KOL og uden hyppige exacerbationer formodes at have stabil KOL og en lavere risiko for fremtidige exacerbationer. Det vurderes derfor relevant at undersøge, om denne gruppe af patienter med stabil KOL fortsat vil have gavn af behandling med ICS. Arbejdsgruppen har valgt at definere patienter med stabil KOL, som patienter med KOL uden moderat til svær exacerbation i mindst ét år, da dette må betragtes som tegn på stabil sygdom.

Population (population)

Patienter på 40 år eller derover med KOL som ikke er nuværende astmapatienter, har været stabile (uden moderate eller alvorlige exacerbationer) i mindst et år og som har været i behandling med ICS mindst 3 måneder inden og/eller under forsøget.

Intervention

Behandling med ICS dagligt i mindst 3 måneder (gælder både nyopstartet eller fortsat behandling med ICS) – enten som monoterapi eller i tilgift til behovsmedicin eller anden inhalationsbehandling (ikke ICS).

Comparison (sammenligning)

Ingen ICS-behandling (men kan have fået ICS-behandlingen forud for forsøgsopstart). Patienter kan være i behandling med behovsmedicin eller få anden inhalationsbehandling (ikke ICS).

Outcome	Tidsramme	Kritisk/ Vigtigt
Exacerbationer pr. år (moderat eller alvorlige)	Længst mulige follow-up (min. 3 mdr. fra intervention start)	Kritisk
Antal patienter med alvorlige bivirkninger pr. år (SAE)	Længst mulige follow-up (min. 3 mdr. fra intervention start)	Kritisk
Pneumoni pr. år	Længst mulige follow-up (min. 3 mdr. fra intervention start)	Vigtigt
Lungefunktion (FEV_1)	Længst mulige follow-up (min. 3 mdr. fra intervention start)	Vigtigt
Dyspnø (fx MRC)	Længst mulige follow-up (min. 3 mdr. fra intervention start)	Vigtigt
Livskvalitet (Fx SGRQ-C, CCQ eller anden relevant test)	Længst mulige follow-up (min. 3 mdr. fra intervention start)	Vigtigt
Frafald	Længst mulige follow-up (min. 3 mdr. fra intervention start)	Kritisk
Antal patienter med en væsentlig forbedring i lungefunktion (FEV1)	Længst mulige follow-up (min. 3 mdr. fra intervention start)	Vigtigt

PICO 5: Bør patienter med KOL og hyppige, tilbagevendende pneumonier fortsat behandles med ICS?

Baggrund for valg af spørgsmål:

Nogle patienter med KOL har en øget risiko for at udvikle pneumoni *[49]*. Pneumoni hos patienter med KOL er forbundet med en øget risiko for exacerbationer, fald i FEV1 og progression af sygdommen *[1]*. Dette sammenholdt med, at studier har vist, at behandling med ICS øger risiko for pneumoni *[50][51]*, gør det derfor relevant at undersøge om patienter med KOL og tendens til pneumoni, fortsat vil have gavn af behandling med ICS. Arbejdsgruppen har valgt at patienter med KOL og mindst to pneumonier det seneste år, må betragtes som havende tendens til hyppige pneumonier, og dermed øget pneumonirisiko.

Population (population

Patienter på 40 år eller derover med KOL, som ikke er nuværende astmapatienter, har haft minimum 2 pneumonier indenfor det seneste år og som har været i behandling med ICS mindst 3 måneder inden og/eller under forsøget.

Intervention

Behandling med ICS dagligt i mindst 3 måneder (gælder både nyopstartet eller fortsat behandling med ICS) – enten som monoterapi eller i tilgift til behovsmedicin eller anden inhalationsbehandling (ikke ICS).

Comparison (sammenligning

Ingen ICS-behandling (men kan have fået ICS-behandlingen forud for forsøgsopstart). Patienter kan være i behandling med behovsmedicin eller få anden inhalationsbehandling (ikke ICS).

Outcome	Tidsramme	Kritisk/ Vigtigt
Exacerbationer pr. år (moderat eller alvorlige)	Længst mulige follow-up (min. 3 mdr. fra intervention start)	Kritisk
Antal patienter med alvorlige bivirkninger pr. år (SAE)	Længst mulige follow-up (min. 3 mdr. fra intervention start)	Kritisk
Pneumoni pr. år	Længst mulige follow-up (min. 3 mdr. fra intervention start)	Kritisk
Lungefunktion (FEV_1)	Længst mulige follow-up (min. 3 mdr. fra intervention start)	Vigtigt
Dyspnø (fx MRC)	Længst mulige follow-up (min. 3 mdr. fra intervention start)	Vigtigt
Livskvalitet (Fx SGRQ-C, CCQ eller anden relevant test)	Længst mulige follow-up (min. 3 mdr. fra intervention start)	Vigtigt
Frafald	Længst mulige follow-up (min. 3 mdr. fra intervention start)	Vigtig
Antal patienter med en væsentlig ændring i lungefunktion (FEV1)	Længst mulige follow-up (min. 3 mdr. fra intervention start)	Vigtigt

Mindste klinisk relevante forskel for kritiske outcomes

Outcome	Mindste klinisk relevante forskel
Exacerbationer pr. år (moderat eller alvorlige)	25 % reduktion i antallet af exacerbationer
Antal patienter med alvorlige bivirkninger pr. år (SAE)	10 % forskel i antallet af personer med alvorlige bivirkninger
Pneumoni pr. år	25 % reduktion i antallet af pneumonier
Frafald	10 % forskel i antallet af personer, der har forladt studiet

Bilag 6: Beskrivelse af anbefalingernes styrke og implikationer

Formulering af evidensbaserede anbefalinger:
En anbefaling kan enten være for eller imod en given intervention. En anbefaling kan enten være stærk eller svag/betinget. Ved evidens vælges en af følgende fire typer af anbefalinger

Stærk anbefaling for (Grøn)
Der gives en stærk anbefaling for, når der er pålidelig evidens, der viser, at de samlede fordele ved interventionen er klart større end ulemperne.
Det er klart, at fordelene opvejer ulemperne. Det betyder, at alle eller næsten alle, patienter vil ønske den anbefalede intervention

Ordlyd: *Giv/brug/anvend…*

Følgende vil trække i retning af en stærk anbefaling for:

Høj eller moderat tiltro til de estimerede effekter.

Stor gavnlig effekt og ingen eller få skadevirkninger.

Patienternes værdier og præferencer er velkendte og ensartede til fordel for interventionen.

Implikationer:
De fleste patienter vurderes at ønske interventionen.
Langt de fleste klinikere vil tilbyde interventionen.

Stærk anbefaling imod (Rød)
Der gives en stærk anbefaling imod, når der er høj tiltro til effektestimaterne, der viser, at de samlede ulemper er klart større end fordelene. Det samme gælder, hvis der er stor tiltro til, at en intervention er nytteløs.

Ordlyd: *Giv ikke/brug ikke/anvend ikke/undlad at…*

Følgende vil trække i retning af en stærk anbefaling imod:

Høj eller moderat tiltro til de estimerede effekter.

Der er stor tiltro til, at interventionen ikke gavner, eller at den gavnlige effekt er lille.

Der er stor tiltro til, at interventionen har betydelige skadevirkninger.

Patienternes værdier og præferencer er velkendte og ensartede imod interventionen.

Implikationer:
De fleste patienter vurderes ikke at ville ønske interventionen.
Klinikeren vil meget sjældent tilbyde interventionen.

Svag anbefaling for (Gul)
Der gives en svag anbefaling for interventionen, når det vurderes, at fordelene ved interventionen er marginalt større end ulemperne, eller den tilgængelige evidens ikke kan udelukke en væsentlig fordel ved en eksisterende praksis, samtidig med at skadevirkningerne er få eller fraværende. Der er større mulighed for variation i individuelle præferencer.

Ordlyd: *Overvej at…*

Følgende vil trække i retning af en svag anbefaling for:

Lav eller meget lav tiltro til de estimerede effekter.

Balancen mellem gavnlige og skadelige virkninger ikke er entydig.

Patienternes præferencer og værdier vurderes at variere væsentligt, eller de er ukendte.

Implikationer:
De fleste patienter vurderes at ønske interventionen, men nogen vil afstå.
Klinikeren vil skulle bistå patienten med at træffe en beslutning, der passer til patientens værdier og præferencer.

Svag anbefaling imod (Orange)
Der gives en svag anbefaling imod interventionen, når ulemperne ved interventionen vurderes at være større end fordelene, men hvor man ikke har høj tiltro til de estimerede effekter. Den svage anbefaling imod, anvendes også hvor der er stærk evidens for både gavnlige og skadelige virkninger, men hvor balancen mellem dem er vanskelig at afgøre.

Ordlyd: *Anvend kun … efter nøje overvejelse, da den gavnlige effekt er usikker og/eller lille, og der er dokumenterede skadevirkninger såsom…*

Følgende vil trække i retning af en svag anbefaling imod:

Lav eller meget lav tiltro til de estimerede effekter. Balancen mellem gavnlige og skadelige virkninger ikke er entydig.

Skadevirkningerne vurderes at være marginalt større end den gavnlige effekt.

Patienternes præferencer og værdier vurderes at variere væsentligt, eller de er ukendte.

Implikationer:
De fleste patienter vurderes at ville afstå fra interventionen, men nogen vil ønske den.
Klinikeren vil skulle bistå patienten med at træffe en beslutning, der passer til patientens værdier og præferencer.

Formulering af anbefaling ved mangel på evidens:
God praksis anbefaling (Grå)

God praksis anvendes, når der ikke foreligger relevant evidens, og bygger således udelukkende på faglig konsensus blandt medlemmerne af arbejdsgruppen. Anbefalingen kan være enten for eller imod interventionen. Da der udelukkende er tale om faglig konsensus, er denne type anbefaling svagere end de evidensbaserede anbefalinger, uanset om de evidensbaseret er stærke eller svage

De to typer af anbefalinger til god praksis anbefalinger

Ordlyd:
For:
Det er god praksis at overveje....

Imod:
Det er ikke god praksis rutinemæssigt at…

Bilag 7: Evidensvurderinger

Risiko for bias-vurderinger og analyser, samt beskrivelse af in- og ekskluderede studier, kan tilgås på Dansk Lungmedicinsk Selskabs, Medicinfunktionens og Sundhedsstyrelsens hjemmeside.

Analyser og Risiko for Bias-vurderinger

- RevMan-filer med risiko for bias-vurderinger og meta-analyser samt beskrivelse af in- og ekskluderede studier kan tilgås på Dansk Lungmedicinsk Selskabs, Medicinfunktionens og Sundhedsstyrelsens hjemmeside.
- Metaanalyser for:
  - PICO 1-3
  - PICO 4
  - PICO 5

Bilag 8: Arbejdsgruppen og referencegruppen

Arbejdsgruppen
Arbejdsgruppen for den nationale kliniske retningslinje for behandling med inhalationssteroid (ICS) hos patienter med kronisk obstruktiv lungesygdom (KOL) består af følgende personer:

- Anders Løkke, udpeget af Dansk Lungemedicinsk Selskab, specialeansvarlig overlæge, Vejle Sygehus
- Charlotte Jensen, speciallæge i almen medicin, Lægerne i Gothersgade
- Dagmar Abelone Dalin, projektleder, farmaceut, Medicinfunktionen, Klinisk Farmakologisk Afdeling, Region Hovedstaden
- Kirsten Birkefoss, søgespecialist
- Mina Nicole Händel, metodekonsulent, cand. scient, ph.d.
- Pernille Kristiansen, formand, 1. reservelæge i lungemedicin, Hillerød Hospital
- Torben Mogensen, udpeget af Lungeforeningen

Habilitetsforhold
Habilitetserklæringerne kan tilgås her.

Referencegruppe
Referencegruppen er udpeget af Dansk Lungemedicinsk Selskab og Medicinfunktionen og dens opgave har bestået i at kommentere på afgrænsningen af og det faglige indhold i retningslinjen.

Referencegruppen for den nationale kliniske retningslinje for behandling med ICS til patienter med KOL består af følgende personer:

- Charlotte Vermehren, formand, farmaceut, ph.d., leder af Medicinfunktionen, Klinisk Farmakologisk Afdeling, Region H
- Hanne Rolighed Christensen, speciallæge i lungemedicin og farmakologi, ledende overlæge, Klinisk Farmakologisk Afdeling, Region H
- Ole Hilberg, speciallæge i lungemedicin, leder af Dansk Lungemedicinsk Selskab
- Nina Skavlan Godtfredsen, speciallæge i lungemedicin, overlæge, Hvidovre Hospital

Peer review og høring
Den nationale kliniske retningslinje for behandling med ICS hos patienter med KOL har forud for udgivelsen været i høring blandt følgende høringsparter:

- Danske Regioner
- Sundheds- og Ældreministeriet
- Lungeforeningen
- Dansk Lungemedicinsk Selskab
- Dansk Selskab for Almen Medicin
- Lægeforeningen
- Danmarks Farmaceutiske Selskab
- Dansk Selskab for Klinisk Farmakologi
- Dansk Sygepleje Selskab
- Danske Patienter
- Dansk Selskab for Kvalitet i Sundhedssektoren

Retningslinjen er desuden i samme periode peer reviewet af:

- Peter Lange, overlæge, Afdeling for Medicinske Sygdomme Herlev og Gentofte hospital
- Charlotte Suppli Ulrik, overlæge, Chef for Lungemedicinsk Forskningsenhed, Hvidovre Hospital

Bilag 9: Begreber og forkortelser

Forkortelse	Forklaring
Absolut effekt	Effekten af en given intervention målt i absolutte tal. Hvis risikoen for at dø af en sygdom f.eks. er 3 per 1,000 og en behandling nedsætter denne risiko til 2 per 1,000, er den absolutte effekt 1 per 1,000. Den tilsvarende relative effekt er en 33% reduktion af dødeligheden, hvilket kan synes mere imponerende, men kan give et misvisende indtryk af den virkelige effekt. Derfor foretrækkes det normalt at præsentere absolutte frem for relative effekter. Bemærk, at den absolutte effekt vil variere med hyppigheden (prævalensen) af et givent udfald, selvom den relative effekt er konstant. Dette kan have betydning, f.eks. ved vurdering af behandlingers relevans i forskellige subgrupper af patienter.
Alvorlige bivirkninger	Bivirkninger (adverse events) defineres som uønskede hændelser inkluderer enhver hændelse, som opstår hos en patient eller en forsøgsperson i et klinisk forsøg, uden at der nødvendigvis er en sammenhæng mellem behandling og hændelse. Alvorlig bivirkning (serious adverse event (SAE)) er en bivirkning, der opfylder én af nedenstående:<br>• resulterer i død<br>• er livstruende<br>• resulterer i indlæggelse eller forlængelse af indlæggelse<br>• kræver handling for at undgå permanent skade<br>• resulterer i vedvarende funktionsnedsættelse
AMSTAR	A Measurement Tool to Assess Systematic Reviews. Værktøj til kvalitetsvurdering af systematiske oversigtsartikler.
Baseline risiko	Ved dikotome udfald (”enten-eller” udfald) betegner det risikoen for et givet udfald ved forsøgets begyndelse. Ved kontinuerte udfald (”udfald målt på en skala”) betegner det en gennemsnitlig målt værdi ved forsøgets begyndelse. Et relateret begreb, assumed risk, findes i Summary of Findings tabellen, hvor det betegner risikoen for et givet udfald i forsøgets kontrolgruppe, eller en risiko i en kontrolgruppe hentet fra f.eks. befolkningsstatistik eller observationelle studier.
Bias	Bias er systematiske fejl i et studie, der fører til over- eller underestimering af effekten
Blinding	Blinding indebærer, at det under forsøget er ukendt hvilken gruppe deltagerne er fordelt til. Flere parter kan være blindet, f.eks. deltageren, behandleren, den der vurderer udfald, og den der analyserer data.
BMI	Body mass index
95%CI	95% Konfidensinterval
Confounding	En confounder er et fænomen, som kan mudre et forskningsresultat således, at man drager ukorrekte konklusioner om sammenhænge mellem årsag og virkning.
DLS	Dansk Lungemedicinsk Selskab
EOS	Blod-eosinofilocytter. Synonymer: blod eosinofile leukocytter, blod eosinofile granulocytter.

	150 celler/μL = 0,15 x 10^9/L<br>300 celler/μL = 0,3 x 10^9/L
FEV1	Lungefunktionen målt som rumfanget af luft pustet ud det første sekund, ved hård udpustning
GOLD	Global Initiative for Chronic Obstructive Lung Disease
GRADE	Grading of Recommendations Assessment, Development and Evaluation
Hazard ratio (HR)	Betegner en ratio mellem to rater af udfald. Hvis raten af dødsfald i en interventionsgruppe for eksempel er 10 per 100, mens den er 20 per 100 i kontrolgruppen, er hazard ratio 2. Angives normalt med et sikkerhedsinterval (konfidensinterval).
Heterogenitet	Inkonsistens imellem resultater fra forskellige studier. Måles f.eks ved I2, se nedenfor
I^2	Angiver den procentdel af variansen i en meta-analyse som skyldes heterogenitet. Heterogenitet udtrykker forskelle i resultaterne i de inkluderede studier. Hvis I2 er høj (>50%) kan det være et udtryk for de inkluderede studier viser forskellige resultater
ICS	Inhalationssteroid
Incidens	Ny begivenhed eller hændelse. Forstås som optrædende for første gang
Intervention	Den behandling/indsats man vil komme med en anbefaling vedrørende.
Klinisk relevant	Et resultat er klinisk relevant, hvis størrelsen af estimatet er relevant for patienterne.
KOL	Kroniske Obstruktiv Lungesygdom
LABA	Langtidsvirkende β2-agonister
LAMA	Langtidsvirkende muskarin antagonist
Mean Difference (MD)	Den gennemsnitlige forskel mellem to grupper.
Metaanalyse	En statistisk metode til at sammenfatte resultaterne af individuelle videnskabelige forsøg, til et overordnet estimat af størrelsen på behandlingseffekter. Behandlingseffektens samlede størrelse kan angives på forskellig måde, for eksempel som en relativ risiko, en odds ratio, eller en standardiseret, gennemsnitlig forskel mellem grupperne (SMD).
Outcome	Udfald. Forhold, man ønsker at måle en effekt på f.eks. smerte, livskvalitet eller død. Outcome vurderes som enten kritiske eller vigtige for at kunne give en anbefaling. De kritiske outcome er styrende for anbefalingen.
PICO	De fokuserede spørgsmål udgør grundlaget for den efterfølgende udarbejdelse af litteratursøgningsstrategi, inklusions- og eksklusionskriterier, fokus ved læsning af

	litteratur, samt formulering af anbefalinger. De fokuserede spørgsmål specificerer patientgruppen, interventionen og effekter. Dette afspejles i akronymet PICO (Population, Intervention, Comparison and Outcomes). For yderligere beskrivelse henvises til metodehåndbogen for nationale kliniske retningslinjer.
Population	Målgruppen for anbefalingen.
Prævalens	Andelen af en population med en bestemt tilstand
Randomiserede forsøg	Studier hvor forsøgsdeltagere fordeles tilfældigt mellem to eller flere grupper, der får forskellig (eller ingen) behandling. Den tilfældige fordeling skal sikre, at de to studiegrupper bliver så ens, at den eneste variation mellem grupperne udgøres af, hvilken behandling personerne tilbydes
RCT	Forkortelse for randomiseret forsøg, kommer af randomised controlled trial
Relativ effekt	Se under Absolut effekt.
Relativ risiko (RR)	Betegner en ratio mellem to rater af udfald. Hvis raten af dødsfald i en interventionsgruppe fx er 10 pr. 100, mens den er 20 pr. 100 i kontrolgruppen, er den relative risiko 2. Angives normal med et sikkerhedsinterval.
SAE	Serious Adverse Events. Se Alvorlige bivirkninger
SGRQ	St. George's Respiratory Questionnaire. Skala til at måle livskvalitet hos lungepatienter. Skalaen går fra 0 til 100, og en højere score betyder flere begrænsninger og lavere livskvalitet. Den mindst klinisk relevante forskel er 4.<br>*[70]*
Statistisk signifikans	Statistisk signifikans betegner, om estimatet af en behandlingseffekt er tilstrækkeligt præcist til, at man vil anse det for usandsynligt, at resultatet er fremkommet ved en tilfældighed. Oftest benytter man et konfidensinterval på 95 % omkring effektestimatet, hvilket vil sige, at det sande effektestimat vil være inkluderet i konfidensintervallet i 95 af 100 forsøg udført på samme måde. Denne grænse er valgt ud fra en konvention, ikke en naturlov, og der er derfor ikke tale om et egentligt videnskabeligt bevis for en effekt, hvis et resultat er statistisk signifikant – der er tale om en sandsynliggørelse.
Standard Deviation (SD)	Standardafvigelse
Standard Error (SE)	Standardfejl
TDI	Transition Dyspnoea Index. Skala til at måle dyspnø. Skalaen går fra minus 9 til plus 9 og en lavere score betyder højere grad af dyspnø. Den mindste klinisk relevante forskel er 1. *[71]*
Moderat exacerbation	Moderate exacerbationer er defineret som behov for oral steroid og/eller antibiotika. .
Svær exacerbation	Svære exacerbationer er defineret som skadestuebesøg og/eller indlæggelse

Bilag 10: Søgebeskrivelse

Søgebeskrivelse
Litteratursøgningen til denne kliniske retningslinje er foretaget i henhold til metodehåndbogen for udarbejdelse af nationale kliniske retningslinjer. Databaserne er udvalgt til søgning efter nationale kliniske retningslinjer som defineret i metodehåndbogen.

Der er foretaget tre systematiske søgninger:
1) En søgning efter kliniske retningslinjer og guidelines (guidelines-søgningen) fra 2010 til april 2020;
2) en opfølgende søgning efter sekundærlitteratur (systematiske reviews og metaanalyser) fra 2010 til juli 2020;
3) en søgning efter supplerende primærlitteratur i hhv. perioderne 2010 til juli 2020 (PICO 1-3) og 2000 til august 2020 (PICO 4).
For PICO 5 er der ikke fundet sekundær litteratur, og der er udelukkende søgt primærstudier fra 2000 til august 2020.

Søgningerne er fortaget ved søgespecialist Kirsten Birkefoss i samarbejde med fagkonsulent Dagmar A. Dalin og Pernille Kristiansen.

Søgeprotokollerne med søgestrategier for de enkelte databaser er tilgængelige nedenfor *[Indsæt link]*.

Generelle søgetermer
Engelske: Chronic obstructive lung disease (COLD), chronic obstructive pulmonary disease (COPD), chronic obstructive lung disorder, chronic obstructive airways disease (COAD), pulmonary emphysema, lung emphysema, chronic bronchitis, obstructive bronchitis, chronic airflow obstruction, chronic airways obstruction, obstructive pulmonary disease
Danske: Kronisk obstruktiv lungesygdom (KOL), kronisk obstruktiv lungelidelse, lungeemfysem, kronisk bronkitis, obstruktiv bronkitis, obstruktiv lungesygdom, kronisk inflammation i luftvejene, kronisk obstruktion af luftvejene
Norske:Kronisk obstruktiv lungesykdom (KOLS, KOL), lungeemfysem, kronisk bronkitt, obstruktiv bronkitt, bronkobstruksjon, obstruktiv lungsjukdom
Svenske: Kronisk lungsjukdom (KOL), kroniskt obstruktiv lungsjukdom, lungeemfysem, kronisk bronkit, obstruktiv bronkit, obstruktiv lungsjukdom

Guidelines-søgningen
Den systematiske søgning efter kliniske retningslinjer, guidelines og MTV'er blev foretaget 13.-14. april 2020 i følgende informationskilder: Guidelines International Network (G-I-N), NICE (UK), Trip Database, Guideline Central, Scottish Intercollegiate Guidelines Network (SIGN), CRD/HTA database, Sundhedsstyrelsen (Danmark), SBU (Sverige), Socialstyrelsen (Sverige), Helsedirektoratet (Norge), Folkehelseinstituttet (Norge), Netpunkt, Helsebiblioteket (Norge), Center for Kliniske Retningslinjer (Danmark), NHMRC (Australien), Canada Medical Association (Canada), CADTH (Canada), WHO, GOLD, Dansk Lungemedicinsk Selskab, Dansk Selskab for Almen Medicin, European Respiratory Society, American Thoracic Society, The Thoracic Society of Australia and New Zealand samt Medline, Embase og Cinahl.

Generelle søgekriterier
Publikations år: 2000-2020
Sprog: Engelsk, dansk, norsk og svensk
Dokumentyper: Guidelines, clinical guidelines, practical guidelines, MTV, HTA, systematiske reviews, metaanalyser, RCT.

Søgning efter systematiske reviews og metaanalyser
Den opfølgende søgning efter systematiske reviews og metaanalyser blev foretaget 1. juli 2020. I søgningen indgik søgestrategier for alle fPICO i databaserne Medline, Embase, Cochrane Library og Cinahl. Alle PICO blev samsøgt. For detaljer, se søgeprotokol og flowcharts.

Søgning efter primære studier
Søgningen blev foretaget 22. juli-12. august 2020 i databaserne Medline, Embase, Cochrane Central og Cinahl. Hhv. PICO 1-3 og PICO 4-5 blev samsøgt. For detaljer, se søgeprotokol og flowcharts.

Søgeprotokoller
Søgeprotokoller for alle PICO kan tilgås herunder:

- Guidelines
- Sekundærlitteratur
- Primærlitteratur

Flowcharts
Flowcharts for alle PICO kan tilgås her:

- PICO 1 (<150 EOS)
- PICO 2 (150-300 EOS)
- PICO 3 (>300 EOS)
- PICO 4 (uden exacerbation i ét år)
- PICO 5 (pneumoni historik)

Referencer

1. Global Initiative for Chronic Obstructive Lung Disease (GOLD) : Global strategy for prevention, diagnosis and management of COPD (2020 report). Global Initiative for Chronic Obstructive Lung Disease (GOLD) 2020;

2. Price D., Yawn B., Brusselle G., Rossi A. : Risk-to-benefit ratio of inhaled corticosteroids in patients with COPD. Primary care respiratory journal : journal of the General Practice Airways Group 2013;22(1):92-100 Journal

3. Borgere med KOL - kontaktforbrug i sundhedsvæsnet og medicinforbrug. Sundhedsdatastyrelsen - Afdeling for sundhedsanalyser, Lægemiddelstatistik og Sundhedsdataprogrammet 2016; Link

4. Savran O, Godtfredsen N, Sørensen T, Jensen C, Ulrik CS : COPD patients prescribed inhaled corticosteroid in general practice: Based on disease characteristics according to guidelines?. Chronic respiratory disease 16 1479973119867949 Pubmed Journal

5. EMA completes review of inhaled corticosteroids for chronic obstructive pulmonary disease. EMA 2016; Link

6. Ulrik CS, Hansen EF, Jensen MS, Rasmussen FV, Dollerup J, Hansen G, et al. : Management of COPD in general practice in Denmark--participating in an educational program substantially improves adherence to guidelines. International journal of chronic obstructive pulmonary disease 2010;5 73-9 Pubmed

7. Drivenes E, Ostrem A, Melbye H : Predictors of ICS/LABA prescribing in COPD patients: a study from general practice. BMC family practice 2014;15 42 Pubmed Journal

8. Brightling CE, McKenna S., Hargadon B., Birring S., Green R., Siva R., et al. : Sputum eosinophilia and the short term response to inhaled mometasone in chronic obstructive pulmonary disease. Thorax 2005;60(3):193-198 Journal

9. Tashkin DP, Wechsler ME : Role of eosinophils in airway inflammation of chronic obstructive pulmonary disease. International journal of chronic obstructive pulmonary disease 2018;13 335-349 Journal

10. Brightling CE, Monteiro W., Ward R., Parker D., Morgan MD, Wardlaw AJ, et al. : Sputum eosinophilia and short-term response to prednisolone in chronic obstructive pulmonary disease: a randomised controlled trial. Lancet (London, England) 2000;356(9240):1480-1485 Journal

11. Pizzichini E., Pizzichini MM, Gibson P., Parameswaran K., Gleich GJ, Berman L., et al. : Sputum eosinophilia predicts benefit from prednisone in smokers with chronic obstructive bronchitis. American journal of respiratory and critical care medicine 1998;158(5 Pt 1):1511-1517 Journal

12. Bafadhel M., McKenna S., Terry S., Mistry V., Pancholi M., Venge P., et al. : Blood eosinophils to direct corticosteroid treatment of exacerbations of chronic obstructive pulmonary disease: a randomized placebo-controlled trial. American journal of respiratory and critical care medicine 2012;186(1):48-55 Journal

13. Leigh R., Pizzichini MM, Morris MM, Maltais F., Hargreave FE, Pizzichini E. : Stable COPD: predicting benefit from high-dose inhaled corticosteroid treatment. The European respiratory journal 2006;27(5):964-971 Journal

14. Bafadhel M., Davies L., Calverley PM, Aaron SD, Brightling CE, Pavord ID : Blood eosinophil guided prednisolone therapy for exacerbations of COPD: a further analysis. The European respiratory journal 2014;44(3):789-791 Journal

15. Global Initiative for Chronic Obstructive Lung Disease (GOLD) : Global strategy for precention, diagnosis and the management of COPD (2019 report). Global Initiative for Chronic Obstructive Lung Disease (GOLD):

16. Bafadhel M., Peterson S., De Blas MA, Calverley PM, Rennard SI, Richter K., et al. : Predictors of exacerbation risk and response to budesonide in patients with chronic obstructive pulmonary disease: a post-hoc analysis of three randomised trials. The Lancet.Respiratory medicine 2018;6(2):117-126 Journal

17. Vedel-Krogh S., Nielsen SF, Lange P., Vestbo J., Nordestgaard BG : Blood Eosinophils and Exacerbations in Chronic Obstructive Pulmonary Disease. The Copenhagen General Population Study. American journal of respiratory and critical care medicine 2016;193(9):965-974 Journal

18. Singh D, Kolsum U, Brightling CE, Locantore N, Agusti A, Tal-Singer R, et al. : Eosinophilic inflammation in COPD: prevalence and clinical characteristics. The European respiratory journal 2014;44(6):1697-700 Pubmed Journal

19. Oshagbemi OA, Burden AM, Braeken DCW, Henskens Y, Wouters EFM, Driessen JHM, et al. : Stability of Blood Eosinophils in Patients with Chronic Obstructive Pulmonary Disease and in Control Subjects, and the Impact of Sex, Age, Smoking, and Baseline Counts. American journal of respiratory and critical care medicine 2017;195(10):1402-1404 Pubmed Journal

20. Schumann DM, Tamm M, Kostikas K, Stolz D : Stability of the Blood Eosinophilic Phenotype in Stable and Exacerbated COPD. Chest 2019;156(3):456-465 Pubmed Journal

21. Long GH, Southworth T, Kolsum U, Donaldson GC, Wedzicha JA, Brightling CE, et al. : The stability of blood Eosinophils in chronic obstructive pulmonary disease. Respiratory research 2020;21(1):15 Pubmed Journal

22. Sennels HP, Jørgensen HL, Hansen A-LS, Goetze JP, Fahrenkrug J : Diurnal variation of hematology parameters in healthy young males: the Bispebjerg study of diurnal variations. Scandinavian journal of clinical and laboratory investigation 2011;71(7):532-41 Pubmed Journal

23. Harries TH, Rowland V, Corrigan CJ, Marshall IJ, McDonnell L, Prasad V, et al. : Blood eosinophil count, a marker of inhaled corticosteroid effectiveness in preventing COPD exacerbations in post-hoc RCT and observational studies: systematic review and meta-analysis. Respiratory Research 2020;21(1):3

24. Chapman KR, Hurst JR, Frent S-M, Larbig M, Fogel R, Guerin T, et al. : Long-Term Triple Therapy De-escalation to Indacaterol/Glycopyrronium in Patients with Chronic Obstructive Pulmonary Disease (SUNSET): A Randomized, Double-Blind, Triple-Dummy Clinical Trial. American Journal of Respiratory & Critical Care Medicine 2018;198(3):329-339

25. Ferguson GT, Rabe KF, Martinez FJ, Fabbri LM, Wang C., Ichinose M., et al. : Triple therapy with budesonide/glycopyrrolate/formoterol fumarate with co-suspension delivery technology versus dual therapies in chronic obstructive pulmonary disease (KRONOS): a double-blind, parallel-group, multicentre, phase 3 randomised controlled trial. The Lancet.Respiratory medicine 2018;6(10):747-758 Journal

26. Papi A., Vestbo J., Fabbri L., Corradi M., Prunier H., Cohuet G., et al. : Extrafine inhaled triple therapy versus dual bronchodilator therapy in chronic obstructive pulmonary disease (TRIBUTE): a double-blind, parallel group, randomised controlled trial. Lancet (London, England) 2018;391(10125):1076-1084 Journal

27. Pascoe S., Locantore N., Dransfield MT, Barnes NC, Pavord ID : Blood eosinophil counts, exacerbations, and response to the addition of inhaled fluticasone furoate to vilanterol in patients with chronic obstructive pulmonary disease: a secondary analysis of data from two parallel randomised controlled trials. The Lancet.Respiratory medicine 2015;3(6):435-442 Journal

28. Siddiqui SH, Guasconi A., Vestbo J., Jones P., Agusti A., Paggiaro P., et al. : Blood Eosinophils: A Biomarker of Response to Extrafine Beclomethasone/Formoterol in Chronic Obstructive Pulmonary Disease. American journal of respiratory and critical care medicine 2015;192(4):523-525 Journal

29. Watz H., Tetzlaff K., Wouters EF, Kirsten A., Magnussen H., Rodriguez-Roisin R., et al. : Blood eosinophil count and exacerbations in severe chronic obstructive pulmonary disease after withdrawal of inhaled corticosteroids: a post-hoc analysis of the WISDOM trial. The Lancet.Respiratory medicine 2016;4(5):390-398 Journal

30. Pascoe S, Barnes N, Brusselle G, Compton C, Criner GJ, Dransfield MT, et al. : Blood eosinophils and treatment response with triple and dual combination therapy in chronic obstructive pulmonary disease: analysis of the IMPACT trial. The Lancet. Respiratory medicine 2019;7(9):745-756 Pubmed Journal

31. Hanania NA, Papi A, Anzueto A, Martinez FJ, Rossman KA, Cappelletti CS, et al. : Efficacy and safety of two doses of budesonide/formoterol fumarate metered dose inhaler in COPD. Erj Open Research 2020;6(2):

32. Goyal P., Rossi A., Bader G., Pablo A. : Comparison of effect of indacaterol with salmeterol/fluticasone fixed dose combination on COPD exacerbations based on baseline blood eosinophil counts: post-hoc analysis from the instead study. Respirology (Carlton, Vic.) 2015;20 43 Journal Link

33. Rabe KF, Martinez FJ, Ferguson GT, Wang C., Singh D., Wedzicha JA, et al. : Triple Inhaled Therapy at Two Glucocorticoid Doses in Moderate-to-Very-Severe COPD. The New England journal of medicine 2020;383(1):35-48 Journal

34. NKR Pulje ICS til KOL patienter PICO 1-3 (EOS-grupper).

35. Metaanalyse for PICO 1-3. Link

36. NKR Pulje ICS til KOL patienter PICO 1-3 (EOS-grupper).

37. NKR Pulje ICS til KOL patienter PICO 1-3 (EOS-grupper).

38. Nannini LJ, Lasserson TJ, Poole P. : Combined corticosteroid and long-acting beta(2)-agonist in one inhaler versus long-acting beta(2)-agonists for chronic obstructive pulmonary disease. The Cochrane database of systematic reviews 2012;2012(9):CD006829 Journal

39. Nannini LJ, Poole P., Milan SJ, Kesterton A. : Combined corticosteroid and long-acting beta(2)-agonist in one inhaler versus inhaled corticosteroids alone for chronic obstructive pulmonary disease. The Cochrane database of systematic reviews 2013;2013(8):CD006826 Journal

40. Hurst JR, Vestbo J., Anzueto A., Locantore N., Müllerova H., Tal-Singer R., et al. : Susceptibility to exacerbation in chronic obstructive pulmonary disease. The New England journal of medicine 2010;363(12):1128-1138 Journal

41. Oba Y, Keeney E, Ghatehorde N, Dias S : Dual combination therapy versus long-acting bronchodilators alone for chronic obstructive pulmonary disease (COPD): a systematic review and network meta-analysis. Cochrane Database of Systematic Reviews 2018;12 012620

42. Doherty DE, Tashkin DP, Kerwin E, Knorr BA, Shekar T, Banerjee S, et al. : Effects of mometasone furoate/formoterol fumarate fixed-dose combination formulation on chronic obstructive pulmonary disease (COPD): results from a 52-week Phase III trial in subjects with moderate-to-very severe COPD. International journal of chronic obstructive pulmonary disease 2012;7 57-71 Pubmed Journal

43. Tashkin DP, Doherty DE, Kerwin E., Matiz-Bueno CE, Knorr B., Shekar T., et al. : Efficacy and safety characteristics of mometasone furoate/formoterol fumarate fixed-dose combination in subjects with moderate to very severe COPD: findings from pooled analysis of two randomized, 52-week placebo-controlled trials. International journal of chronic obstructive pulmonary disease 2012;7 73-86 Journal

44. Rossi A, van der Molen T, del Olmo R, Papi A, Wehbe L, Quinn M, et al. : INSTEAD: a randomised switch trial of indacaterol versus salmeterol/fluticasone in moderate COPD. The European respiratory journal 2014;44(6):1548-56 Journal Link

45. Martinez F., Ferguson G., Bourne E., Ballal S., Darken P., DeAngelis K., et al. : Budesonide/glycopyrrolate/formoterol fumarate metered dose inhaler, formulated using co-suspension delivery technology, improves lung function and exacerbation outcomes in patients with COPD without a recent history of exacerbations: subgroup analysis of KRONOS study. Chest 2019;156(4):A2276-A2277 Journal Link

46. NKR Pulje PICO 4: ICS til patienter med KOL efter mindst et Ã¥r uden moderate/alvorlige exacerbationer.

47. Ferguson GT, Anzueto A, Fei R, Emmett A, Knobil K, Kalberg C : Effect of fluticasone propionate/salmeterol (250/50 microg) or salmeterol (50 microg) on COPD exacerbations. Respiratory medicine 2008;102(8):1099-108 Pubmed Journal

48. Metaanalyse for PICO 4 inkl. sensitivitetsanalyse. Link

49. Müllerova H., Chigbo C., Hagan GW, Woodhead MA, Miravitlles M., Davis KJ, et al. : The natural history of community-acquired pneumonia in COPD patients: a population database analysis. Respiratory medicine 2012;106(8):1124-1133 Journal

50. Crim C., Dransfield MT, Bourbeau J., Jones PW, Hanania NA, Mahler DA, et al. : Pneumonia risk with inhaled fluticasone furoate and vilanterol compared with vilanterol alone in patients with COPD. Annals of the American Thoracic Society 2015;12(1):27-34 Journal

51. Yang IA, Clarke MS, Sim EH, Fong KM : Inhaled corticosteroids for stable chronic obstructive pulmonary disease. The Cochrane database of systematic reviews 2012;(7):CD002991. doi(7):CD002991 Journal

52. Metaanalyse for PICO 5 inkl. sensitivitetsanalyse. Link

53. Fokuserede spørgsmål 1-5.

54. Aaron SD, Vandemheen KL, Fergusson D., Maltais F., Bourbeau J., Goldstein R., et al. : Tiotropium in combination with placebo, salmeterol, or fluticasone-salmeterol for treatment of chronic obstructive pulmonary disease: a randomized trial. Annals of Internal Medicine 2007;146(8):545-555 Journal

55. Mahler DA, Witek TJ : The MCID of the transition dyspnea index is a total score of one unit. Copd 2005;2(1):99-103 Journal

56. Anzueto A., Ferguson GT, Feldman G., Chinsky K., Seibert A., Emmett A., et al. : Effect of fluticasone propionate/salmeterol (250/50) on COPD exacerbations and impact on patient outcomes. Copd 2009;6(5):320-329 Journal

57. Calverley P., Pauwels R., Vestbo J., Jones P., Pride N., Gulsvik A., et al. : Combined salmeterol and fluticasone in the treatment of chronic obstructive pulmonary disease: a randomised controlled trial. Lancet (London, England) 2003;361(9356):449-456 Journal

58. Calverley PMA, Anderson JA, Brook RD, Crim C., Gallot N., Kilbride S., et al. : Fluticasone Furoate, Vilanterol, and Lung Function Decline in Patients with Moderate Chronic Obstructive Pulmonary Disease and Heightened Cardiovascular Risk. American journal of respiratory and critical care medicine 2018;197(1):47-55 Journal

59. Choudhury AB, Dawson CM, Kilvington HE, Eldridge S., James WY, Wedzicha JA, et al. : Withdrawal of inhaled corticosteroids in people with COPD in primary care: a randomised controlled trial. Respiratory research 2007;8(1):93 Journal

60. Dransfield MT, Bourbeau J., Jones PW, Hanania NA, Mahler DA, Vestbo J., et al. : Once-daily inhaled fluticasone furoate and vilanterol versus vilanterol only for prevention of exacerbations of COPD: two replicate double-blind, parallel-group, randomised controlled trials. The Lancet.Respiratory medicine 2013;1(3):210-223 Journal

61. Dransfield MT, Feldman G., Korenblat P., LaForce CF, Locantore N., Pistolesi M., et al. : Efficacy and safety of once-daily fluticasone furoate/vilanterol (100/25 mcg) versus twice-daily fluticasone propionate/salmeterol (250/50 mcg) in COPD patients. Respiratory medicine 2014;108(8):1171-1179 Journal

62. Ferguson GT, Tashkin DP, Skärby T., Jorup C., Sandin K., Greenwood M., et al. : Effect of budesonide/formoterol pressurized metered-dose inhaler on exacerbations versus formoterol in chronic obstructive pulmonary disease: The 6-month, randomized RISE (Revealing the Impact of Symbicort in reducing Exacerbations in COPD) study. Respiratory medicine 2017;132 31-41 Journal

63. Lipson DA, Barnhart F., Brealey N., Brooks J., Criner GJ, Day NC, et al. : Once-Daily Single-Inhaler Triple versus Dual Therapy in Patients with COPD. The New England journal of medicine 2018;378(18):1671-1680 Journal

64. Ohar JA, Crater GD, Emmett A., Ferro TJ, Morris AN, Raphiou I., et al. : Fluticasone propionate/salmeterol 250/50 µg versus salmeterol 50 µg after chronic obstructive pulmonary disease exacerbation. Respiratory research 2014;15(1):105-014-0105-2 Journal

65. Papi A., Dokic D., Tzimas W., Mészáros I., Olech-Cudzik A., Koroknai Z., et al. : Fluticasone propionate/formoterol for COPD management: a randomized controlled trial. International journal of chronic obstructive pulmonary disease 2017;12 1961-1971 Journal

66. Siddiqui SH, Pavord ID, Barnes NC, Guasconi A., Lettis S., Pascoe S., et al. : Blood eosinophils: a biomarker of COPD exacerbation reduction with inhaled corticosteroids. International journal of chronic obstructive pulmonary disease 2018;13 3669-3676 Journal

67. van der Valk P., Monninkhof E., van der Palen J., Zielhuis G., van Herwaarden C. : Effect of discontinuation of inhaled corticosteroids in patients with chronic obstructive pulmonary disease: the COPE study. American journal of respiratory and critical care medicine 2002;166(10):1358-1363 Journal

68. Wouters EF, Postma DS, Fokkens B., Hop WC, Prins J., Kuipers AF, et al. : Withdrawal of fluticasone propionate from combined salmeterol/fluticasone treatment in patients with COPD causes immediate and sustained disease deterioration: a randomised controlled trial. Thorax 2005;60(6):480-487 Journal

69. Habilitetserklæringer for arbejdsgruppen.

70. Welling JBA, Hartman JE, Ten Hacken NHT, Klooster K, Slebos D-J : The minimal important difference for the St George's Respiratory Questionnaire in patients with severe COPD. The European respiratory journal 2015;46(6):1598-604 Pubmed Journal

71. Witek TJ, Mahler DA : Minimal important difference of the transition dyspnoea index in a multinational clinical trial. The European respiratory journal 2003;21(2):267-72 Pubmed

72. Litteraturgennemgangsflowchart PICO 1. Link

73. Litteraturgennemgangsflowchart PICO 2. Link

74. Litteraturgennemgangsflowchart PICO 3. Link

75. Litteraturgennemgangsflowchart PICO 4. Link

76. Litteraturgennemgangsflowchart PICO 5. Link

77. Dansk Lungemedicinsk Selskab : Søgeprotokol for NKR om ICS til patienter med KOL - primærlitteratur. 2021; Link

78. Dansk Lungemedicinsk Selskab : Søgeprotokol for NKR om ICS til patienter med KOL - guidelinesøgning. 2021; Link

79. Dansk Lungemedicinsk Selskab : Søgeprotokol for NKR om ICS til patienter med KOL - reviewsøgning. 2021; Link
